# Cost of Dengue Illness in Indonesia across Hospital, Ambulatory, and not Medically Attended Settings

**DOI:** 10.4269/ajtmh.19-0855

**Published:** 2020-09-08

**Authors:** Nandyan N. Wilastonegoro, Dinar D. Kharisma, Ida S. Laksono, Yara A. Halasa-Rappel, Oliver J. Brady, Donald S. Shepard

**Affiliations:** 1 Faculty of Medicine, Public Health and Nursing, Gadjah Mada University, Yogyakarta, Indonesia;; 2 Heller School for Social Management and Policy, Brandeis University, Waltham, Massachusetts;; 3 Pediatrics Department, Faculty of Medicine, Public Health and Nursing, Dr. Sardjito General Hospital, Gadjah Mada University, Yogyakarta, Indonesia;; 4 Centre for Mathematical Modelling of Infectious Diseases, London School of Hygiene & Tropical Medicine, London, United Kingdom;; 5 Department of Infectious Disease Epidemiology, Faculty of Epidemiology and Public Health, London School of Hygiene & Tropical Medicine, London, United Kingdom

## Abstract

Informed decisions concerning emerging technologies against dengue require knowledge about the disease’s economic cost and each stakeholder’s potential benefits from better control. To generate such data for Indonesia, we reviewed recent literature, analyzed expenditure and utilization data from two hospitals and two primary care facilities in Yogyakarta city, and interviewed 67 dengue patients from hospital, ambulatory, and not medically attended settings. We derived the cost of a dengue episode by outcome, setting, and the breakdown by payer. We then calculated aggregate Yogyakarta and national costs and 95% uncertainty intervals (95% UIs). Dengue costs per nonfatal case in hospital, ambulatory, not medically attended, and overall average settings were US$316.24 (95% UI: $242.30–$390.18), US$22.45 (95% UI: $14.12–$30.77), US$7.48 (95% UI: $2.36–$12.60), and US$50.41 (95% UI: $35.75–$65.07), respectively. Costs of nonfatal episodes were borne by the patient’s household (37%), social contributors (relatives and friends, 20%), national health insurance (25%), and other sources (government, charity, and private insurance, 18%). After including fatal cases, the average cost per episode became $90.41 (95% UI: $72.79–$112.35). Indonesia had an estimated 7.535 (95% UI: 1.319–16.513) million dengue episodes in 2017, giving national aggregate costs of $681.26 (95% UI: $232.28–$2,371.56) million. Unlike most previous research that examined only the formal medical sector, this study included the estimated 63% of national dengue episodes that were not medically attended. Also, this study used actual costs, rather than charges, which generally understate dengue’s economic burden in public facilities. Overall, this study found that Indonesia’s aggregate cost of dengue was 73% higher than previously estimated, strengthening the need for effective control.

## INTRODUCTION

Dengue is a common cause of hospitalization in endemic areas of tropical countries. Moreover, dengue cases have grown dramatically, with recent annual aggregate estimates of 58 million,^1^ 100 million,^2^ and 105 million cases globally.^3^ Similarly, the worldwide disability-adjusted life years (DALYs) due to dengue grew from 822,800 in 1990 to 2,920,000 in 2017.^4^ As this rise runs counter to the global shift in disease burden from communicable to noncommunicable diseases,^5^ it highlights the need for better understanding and improved tools.

Many dengue infections are asymptomatic. Dengue episodes (symptomatic infections) range in severity from undifferentiated fever to severe and occasionally fatal cases. During the acute phase, sick individuals often experience high fever lasting for more than 24 hours, and other symptoms may also be present, for example, retro-orbital pain, nausea, vomiting, rash, aches, and pain. In severe dengue, the warning signs might appear as persistent vomiting, clinical fluid accumulation, mucosal bleeding, lethargy, and concurrent increasing of hematocrit with rapid decreasing of platelet count. This severe form leads to severe plasma leakage or shock, severe bleeding, and severe organ impairment.^6^ These symptoms impose a range of direct costs (i.e., costs for medical care) and indirect costs (i.e., the economic value of lost productivity) on affected families, with cost increasing with disease severity. Some episodes persist for months.^7^ Since the first case found in Surabaya city in 1968,^8^ Indonesia has become one of the countries with the highest burden of dengue.^9^

Several promising tools have recently been announced or are in the pipeline to control dengue, for example, vaccines, insecticide-treated materials, lethal ovitraps, spatial repellents, genetically modified mosquitoes, and *Wolbachia*-infected *Aedes aegypti*.^10^ In 2016, the government of Indonesia scaled up community-based vector control to sensitize households to monitor and eliminate mosquitoes.^11^ Furthermore, Indonesia was one of the 10 countries in the phase three trials leading to the first licensed dengue vaccine.^12^ With its combination of high dengue burden, scientific capacity, support from a national foundation, and leadership from the region’s governor, Yogyakarta was the site of the first cluster-randomized trial for testing wMel *Wolbachia*-infected *Ae. aegypti* as a replacement for wild mosquitoes to inhibit dengue transmission.^13^

However, the continued development and, if warranted, deployment of each of these strategies would need funding. Here, we measure the economic impact of dengue from the health system and societal perspectives in Indonesia. Estimating dengue economic burden is important to inform policy-makers in setting policy priorities, implementing novel technologies, and estimating the trade-offs. In this study, we recruited and interviewed representative, and in some cases randomly chosen, patients from both the formal healthcare system and outside the medical sector. Thus, our approach sought to overcome the challenges that few dengue episodes are hospitalized (the setting with the best data) and many are managed outside of the medical sector (the setting with the least data), and that accuracy required empirical data rather than assumptions and expert opinion.

To interpret our findings and guide policy-makers, we synthesized previous work as a basis for comparison. Hence, this study refines cost estimates for dengue illness, incorporating the direct and indirect costs of treatment across three treatment settings (hospitalized, ambulatory, and not medically attended) and breaking down funding among payers (household, government, social insurance, household contributions, and health facility cross-subsidies). The results indicate how much each payer would save if it were possible to eliminate dengue.

## MATERIALS AND METHODS

### Data collection.

We focused on Yogyakarta, Indonesia, a global center on dengue research, and selected the recruitment period of January through June 2018 for a prospective study. The city’s medium size (400,000 residents) and strong surveillance system suggested that it could provide representative and reasonably accurate data. Working from the existing surveillance system, the Yogyakarta City Health Office provided study investigators with numbers of reported dengue cases in Yogyakarta city before and during the study period. The records indicated the first half of 2018 had fewer cases than the corresponding period in 2017.

**Figure 1.**
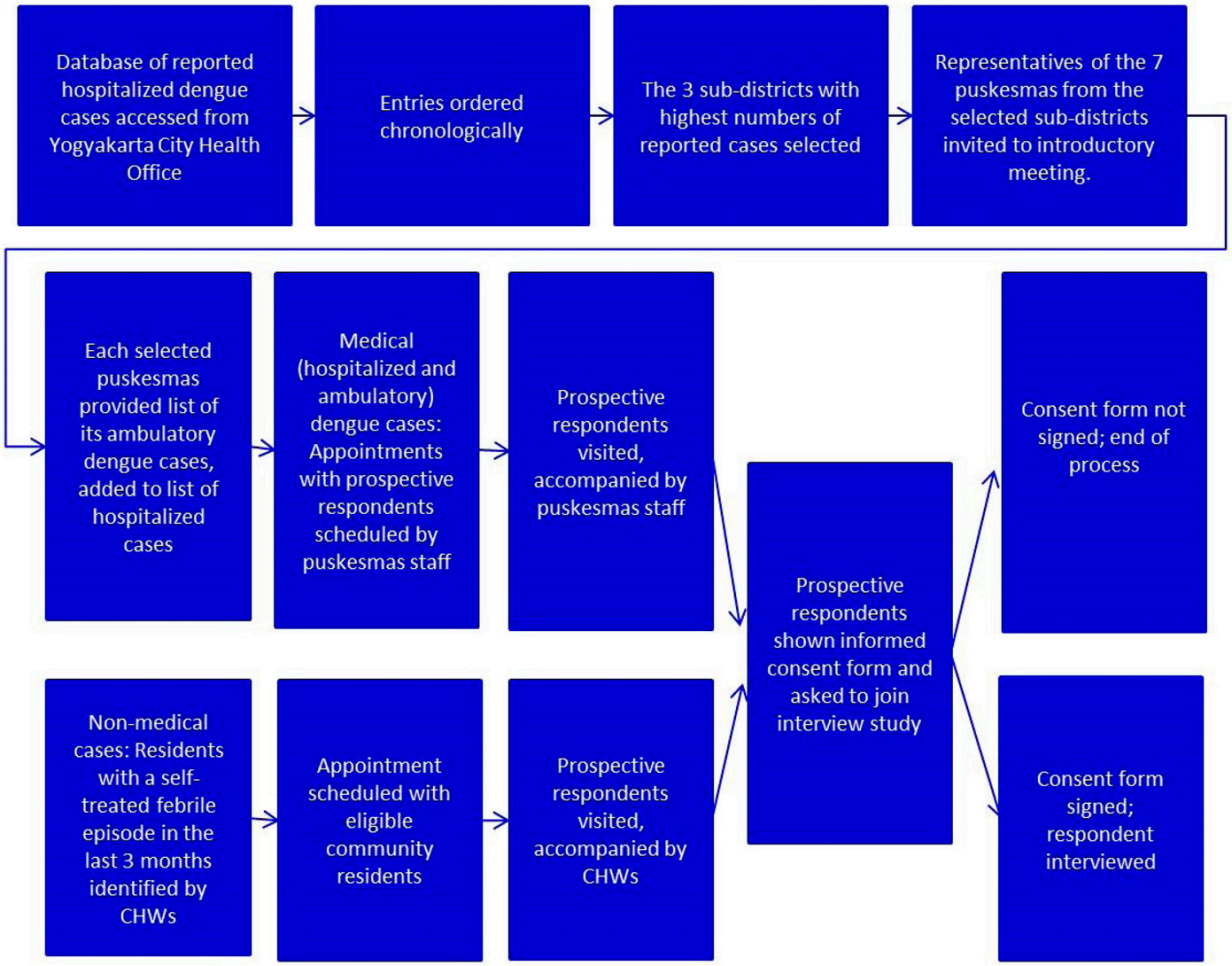
Flowchart of process for identifying and interviewing respondents. CHW = community health worker. This figure appears in color at www.ajtmh.org.

Figure 1 describes the process of selecting and interviewing study participants during the prospective period. Among 14 subdistricts, we selected the three subdistricts with the highest number of dengue cases, that is, Kotagede, Umbulharjo, and Mantrijeron. We compiled a list of both suspected and hospitalized dengue cases during the prospective period from the catchment area of seven puskesmas (public health centers) within the three subdistricts in Yogyakarta city. Then, we invited the clinical coordinator and surveillance chief from each of the seven puskesmas to a meeting for a study briefing and data needs.

We identified hospitalized cases through reports that the puskesmas received from hospitals concerning patients from their catchment areas who were discharged with a final dengue diagnosis (based on clinical patterns, hematology tests, and, if available, confirmatory tests such as a positive nonstructural protein 1 (NS1) test or positive serological test). We identified ambulatory cases at these same puskesmas of suspected dengue cases on their dengue registries. The puskesmas conducted NS1 confirmation when supplies allowed (provided through the World Mosquito Program^14^ in Indonesia or the Yogyakarta municipal health department). We collated all hospitalization and ambulatory cases from January to June 2018. We used the random function in Excel to randomly select patients for an interview. Then, puskesmas staff invited the affected families to participate.

Our sample-size calculations were based on results from the national social–economic survey which found that among individuals aged 15 years or older, 6.8% experienced fever in the previous month.^15^ Because fever is the most common symptom of a mild dengue infection,^16^ we assumed that fever cases in the community could sometimes be dengue episodes, often not reported. We identified suspected not medically attended dengue cases through community meetings by finding participants with a recent history of fever who self-treated or were treated at a nonformal setting (such as a nurse’s home). We collated names of attendees at all community meetings undertaken from April to June 2018, and from those lists used Excel to randomly select individuals to be invited for interview who had fever during the past 1–2 months but did not access formal medical care. Community health workers (CHWs) then set up appointments with the selected individuals, and we performed the interviews.

For participants at all levels of disease severity, we arranged appointments for systematic interviews; obtained informed consent (received for 99% of cases); administered standardized questionnaires to capture the out-of-pocket expenditures, direct nonmedical costs (i.e., costs incurred during medical care, such as additional meals and transport costs), and indirect costs (i.e., the economic value of lost productivity); and provided refreshments as a benefit of participating to the study. Our survey instrument measured the patients’ quality of life, duration of illness, use of health services, the impact on schooling or work productivity and leisure time, out-of-pocket payments, and income lost, as well as the time and income loss of caregivers due to the patients’ illness. The Supplemental Information contains the questionnaires (S1: Adult questionnaire in English; S2: Adult questionnaire in Bahasa Indonesia; S3: Pediatric questionnaire [patients under age 18] in English; S4: Pediatric questionnaire [patients under age 18] in Bahasa Indonesia). The final sample consisted of 67 cases, among which 20 were confirmed hospitalized dengue cases, 24 were clinically diagnosed as dengue by the physician at the health center, and 23 were fever cases treated outside the professional healthcare system.

### Calculating dengue cost.

The total cost per case was calculated by adding three components: direct medical (i.e., costs for medical procedures), direct nonmedical, and indirect costs. Direct medical costs for an episode include the treatment facility’s unit cost, plus household’s purchase of over-the-counter medicines. To calculate the unit cost of each hospital service, we applied the macro-costing approach, also known as the relative cost approach, to estimate the cost of a bed day and a typical outpatient service.^17^ Indonesia has four types of hospitals based on the Ministry of Health’s categorization.^18^ Hospitals of types A and B are advanced hospitals. Type A hospitals (national referral hospitals), which are few in number, have the most specialists and subspecialists and an intensive care unit. Most of Indonesia’s advanced hospitals are type B, which have four types of basic specialists, 12 other specialists, two subspecialists, and an intensive care unit. Type C hospitals have at least six additional types of specialists and an intensive care unit. Type D, the most basic type of hospital, has at least two types of basic specialists. Hospitals of each type can be either public or private (generally not for profit). We collected the hospital’s operating cost data, occupancy rates, and number of registered beds through interviews with one type B and one type D hospitals, both of which happened to be private.

We divided the annual operating cost by the annual number of total bed days to obtain per night hospitalization unit cost. We then calculated hospital outpatient cost as 32% of per night inpatient care cost.^17^ We calculated a clinic’s cost per visit (which typically includes consultation, prescribed medicines, and laboratory test cost) using an ingredients approach. We interviewed puskesmas staff and family physicians to obtain salaries and asked administrators for non-personnel costs (e.g., medicines, supplies, electricity, rent, and utilities).

The direct nonmedical cost consists of transport and additional meals for the patient and care-givers. The indirect cost accounts for the income lost by the sick individual or other household members because of the treatment or care requirements. This study relies on respondents’ reports of actual income lost. We surveyed families after the illness episode so that data record the full episode, including the period after discharge for hospitalized patients. To aid recall, the interviewer showed the respondent a calendar to identify the dates of onset of fever, and hospital admission and discharge where applicable. Because most of our cases were children, this component also includes the adults’ income lost when uncompensated time off from work was required.

However, many adults lost time but little or no income. For instance, some adults were civil servants with paid leave. They lost an attendance-based allowance, but not their basic salary. Unemployed pensioners experienced no actual income loss. For example, retired civil servants and private-sector retirees with pensions continued to receive their monthly allowances. Family members providing assistance outside working hours also experienced no actual income loss. Self-employed family members (e.g., owners of food stalls) provided care when their business was not open or adjusted their purchasing of ingredients and employee activities, so they also generally avoided income loss.

Similarly, daily workers, who would have lost income had they missed a day of work, endeavored to minimize their income loss by asking other family members to attend to the patient. Our questionnaire also elicited descriptive reports about loss of productive time or school days that did not result in income loss. We used the most recent economic data from each source, which was either calendar year 2017 or early 2018. Indonesia’s 2018 inflation was 3.2%,^19^ so in the first quarter year, it averaged 0.8%. As we had the most data for 2017, we considered all costs as occurring in that year. We converted Indonesian rupiah (Rp) to United States dollars (US$) at the weighted average exchange rate over the study period of December 2017 through July 2018.^20^ The weighting combined the exchange rates at the end of each of 8 months according to the number of days in each month. We then adjusted the amount to 2017 US$ using the change in U.S. per capita Gross Domestic Product (GDP).^21^

To calculate the 95% uncertainty interval (UI) of sample data on utilization and costs per episode, we fit a normal distribution to each item based on the observed mean and SDs (standard deviations, the simplest distribution for our descriptive objective). If the lower bound gave a negative number, then we truncated it at 0.00 to avoid artifacts from the normal distribution.

For fatal cases, this study applied the cost estimation conducted in a global study by Shepard et al.,^1^ which used a human capital approach to estimate the indirect cost of fatal cases in Indonesia as $100,000 and $65,000 per case for children and adults, respectively, in 2013. That study used the discounted remaining life expectancy at the average age of death for child and adult deaths and valued each year at Indonesia’s per capita gross national income (GNI). This calculation is consistent with Indonesian culture and observations in our study, where many adults are outside the formal sector. Those older than 65 years often continue in economically productive activities in agriculture, business, or caring for grandchildren. To account for the economic changes since 2013, we adjusted the costs using Indonesia’s 2017 GNI per capita^19^ growth during those 4 years in current US$ at market exchange rates. This adjustment incorporates both inflation and growth in real income, which made the economic loss of each fatality greater. This study then proportionally applied the 95% UI of global fatal cost reported in the Shepard study to estimate the lower and upper bounds per case for dengue death cost in Indonesia. We calculated estimates of $106,247 (95% UI: $98,374–$125,622) and $69,061 (95% UI: $63,943–$81,654) for children and adults, respectively, per case fatal cost in 2017.

### Estimating coverage of alternative payment sources.

We collected additional information to understand how and by whom the cost of a dengue episode is covered. A question was included on the survey regarding friends’ and family’s contribution to see whether households received cash or in-kind support during the illness episode. This study also asked respondents about their household’s out-of-pocket payments and whether their medical costs were fully or partially covered by Indonesia’s national health insurance program, Jaminan Kesehatan Nasional (JKN). Jaminan Kesehatan Nasional is arguably the world’s largest single-payer comprehensive insurance program, with coverage as of May 31, 2019 of 222,002,996 people, 84% of Indonesia’s population.^22^

We used the latest JKN reimbursement tariff, issued and regulated by the Indonesian Ministry of Health in 2016, to estimate the amount of each dengue episode that would be reimbursed by the program.^23^ For inpatient care, the tariff varies by hospital status, type, regional location, and disease severity level. Recent JKN’s regulations limited dengue cases with mild severity normally to be referred only to type C or D hospitals.^24^ Based on our discussions with hospital officials, we estimated that 20% of hospitalized dengue patients were treated in type A or B hospitals because they had medium- or severe-level dengue or lower hospital types were filled, whereas the remaining 80% had mild-level dengue and were admitted to type C and D hospitals. To estimate the amount reimbursed to the hospital by JKN for each episode, we used the rate applied in Yogyakarta city area and the preceding severity levels.

The residual gap between the total cost and all accounted payments for dengue cases (out-of-pocket expenditure, friends’ and family’s contribution, and JKN coverage) was considered coverage by other programs. This other coverage included other government subsidies, nongovernment organization/foundation subsidies, or payment from private insurance.

### Estimating numbers of dengue cases.

We used the results from O’Reilly et al.^25^ to determine the number of dengue cases in Indonesia and Yogyakarta city on four severity levels: ambulatory, hospitalized, self-treated, and fatal cases. That study applied a model ensemble approach to estimate the number of dengue cases in Indonesia. It applied multiple previously published modeling approaches to Indonesian data to give long-term average case estimates for the target year of 2015. As this is a long-term (5+ years) average estimate, we assumed these predictions were representative of dengue burden in our target year of 2017. That study then used health-seeking behavior patterns of the population aged 5+ years with an experience of fever in the last month, from the Indonesia National Socioeconomic Survey (SUSENAS) data of 2014, to estimate the proportion of dengue cases that were not treated at formal healthcare settings.^15^ Furthermore, the control arm of the Sanofi-Pasteur vaccine trial was used to estimate the hospitalization rate among dengue patients who used formal medical care.^26^ O’Reilly et al.^25^ estimated that 35.9% (95% UI: 35.4–36.3) of dengue cases in Indonesia were treated in formal health facilities, and of those seeking formal treatment, 35.2% (95% UI: 33.3–37.2) were hospitalized.

Using O’Reilly et al.^25^ findings, we then conducted a further exercise to estimate the nonfatal cases breakdown specific for Yogyakarta city and the distribution of fatal cases between adults and children. Yogyakarta city has a considerably higher healthcare utilization rate than the national average. Also, using SUSENAS 2014, we estimated that 52% of dengue cases in Yogyakarta were treated in a formal medical care setting.^15^ We applied Shepard’s distribution of adults and children dengue fatal cases to estimate the 2017 adults–children fatal cases distribution nationally and in Yogyakarta city.^1^ The Shepard study created a dengue death model based on data of fatal dengue cases, healthcare access, and GDP per capita of 52 countries, and estimated that of all fatal dengue cases in Indonesia, about 36% were children. The 95% UI for Yogyakarta city estimation and distribution of fatal cases were then estimated proportionally using the 95% UI of the national and total death case estimates.

### Calculating aggregate burden.

We calculated the aggregate national and Yogyakarta city burden of dengue by multiplying the number of cases by the per-case cost by treatment setting. As estimates of numbers of dengue cases were skewed right, we fitted a logarithmic distribution to the numbers of cases. For consistency in calculating aggregate costs, we also fitted a logarithmic distribution to the total cost per case by setting. We then exponentiated the result to determine the actual means and UIs by setting. We estimated total costs across settings by summing the corresponding means. We assumed that the distributions were highly correlated across settings (as overall incidence was a major common factor) and estimated the upper and lower 95% UIs by summing the corresponding UI bounds for each setting. We then applied the per-case distribution of sources of payment in every setting on the aggregated data and obtained the aggregated burden and share of each financing source nationally and in Yogyakarta city.

### Literature review.

To provide context for our empirical analysis, we conducted a preliminary literature review using two databases: the U.S. National Library of Medicine (PubMed) and Google Scholar. The search terms used were “dengue” AND “cost” OR “dengue” AND “economic” combined with English language. We reviewed the titles of the resulting 1,100 citations, retaining those which had full text available and possibly discussed dengue costs in a specific country or countries, region, or worldwide. We reviewed the abstract and full text of the resulting 26 citations to examine whether article 1) reported cost per episode in a country, selected countries, region, or worldwide; and 2) provided detail cost by treatment setting that is hospitalized and/or ambulatory case, finding 15 articles for complete analysis. To standardize costs across the various countries and years, we used GNI per capita.^19^

Then, we calculated the ratio of Indonesia’s 2017 GNI per capita with the GNI per capita of each country, region, and worldwide within the eligible studies. For those 15 eligible articles, we adjusted costs to 2017 US$ as this study’s benchmark. Thus, we were able to generate information on cost per dengue episode in each country measured and compare these with values for Indonesia in 2017 (see Supplemental Tables S5 and S6).

### Ethical approval.

This study was approved by both the Gadjah Mada University and Brandeis University ethics boards.

## RESULTS

### Sample of dengue cases.

Most hospitalized and ambulatory cases were in children. About 90% of hospitalized cases and 92% of ambulatory cases were aged 18 years or younger when they experienced dengue. For those seeking formal health care, the average number of outpatient care visits per episode was 1.71 (95% UI: 1.29–2.13) for ambulatory and 2.35 (95% UI: 1.74–2.96) for hospitalized settings. The length of stay averaged 4.05 (95% UI: 3.47–4.63) days for hospitalized cases. For about 80% of ambulatory care respondents and 88% of hospitalized respondents, JKN funded all or most of their care. This pattern is consistent with the higher utilization of puskesmas and type C/D hospitals. The not medically attended cases were mostly managed with over-the-counter or herbal medicines (see Table 1).

**Table 1:** Description and healthcare utilization of sampled nonfatal dengue cases

Item	Total	Hospitalized	Ambulatory	Not medically attended
Sample size	67	20	24	23
Demographics				
Average age (years)	19.54	12.67	7.31	38.57
95% UI	14.95–24.12	8.97–16.36	4.69–9.94	29.84–47.29
Age, < 18 years (%)	67	90	92	22
Gender, male (%)	44	43	67	22
Number of respondents using formal health care				
Treated in primary care	32	15	23	0
Puskesmas	30	7	23	0
Private clinic	11	8	3	0
Treated in hospital, including outpatient department, including emergency department	27	20	7	0
Type A/B hospital	11	8	3	0
Type C/D hospital	14	12	2	0
Hospital type not classified	2	0	2	0
Average number of ambulatory care	1.33	2.35	1.71	0.04
Visits per episode, 95% UI	1.00–1.66	1.74–2.96	1.29–2.13	−0.05–0.13
Average number of hospitalization	1.21	4.05	0.00	0.00
Days per episode, 95% UI	1.04–1.38	3.47–4.63	0.00–0.00	0.00–0.00
Used JKN to cover any services (%)	55	80	88	0

JKN = Jaminan Kesehatan Nasional; UI = uncertainty interval. Jaminan Kesehatan Nasional is Indonesia’s national health insurance system. Some respondents used more than one type of facility, so components may not sum to the totals.

### Cost per dengue episode.

This study found that the total costs of dengue per case in ambulatory care and hospitalized care settings were $22.45 (95% UI: $14.12–$30.77) and $316.24 (95% UI: $242.30–$390.18), respectively. About 66% (ambulatory setting) and 82% (hospitalized setting) of these costs were direct medical expenses, covering medicines and medical services in the facilities. For the ambulatory care setting, the direct medical cost of hospital outpatient cases was $34.19 (95% UI: $15.68–$52.71), or about five times the cost of puskesmas cases. The average cost of hospitalized cases in type A/B hospitals, $372.99 (95% UI: $236.69–$509.30), was about twice the average cost of hospitalized cases in type C/D hospitals. The average cost of not medically attended cases was $7.48 (95% UI: $2.36–$12.60). This cost consisted mostly of direct nonmedical expenses, such as additional meals and income lost. Only about 12% of these costs were spent on over-the-counter medicines or not medically attended (e.g., visits to traditional healers, see Table 2). Although the actual distribution of costs per episode tended to be skewed to the right, the normal distribution was simpler and provided a reasonable approximation of costs of hospitalized cases. The detailed distribution of each component of the cost of hospitalized cases is shown in Supplemental Figures S8–S11.

**Table 2:** Estimated cost per nonfatal dengue episode by component and setting (2017 US$)

	Component				
Treatment setting	Direct medical	Direct not medical	Indirect	% Positive*	Total cost
Ambulatory (all)	14.89 (7.96–21.81)	5.31 (2.82–7.81)	2.24 (0–4.54)	29 (10–49)	22.45 (14.12–30.77)
Puskesmas	6.94 (5.67–8.20)	3.46 (1.67–5.24)	2.66 (0–5.85)	29 (5–54)	13.05 (8.83–17.27)
Hospital outpatient	34.19 (15.68–52.71)	9.83 (2.20–17.46)	1.24 (0–3.98)	29 (0–74)	45.26 (25.57–64.95)
Hospitalized (all)	260.62 (195.27–325.97)	33.95 (23.61–44.30)	21.67 (8.81–34.54)	65 (42–88)	316.24 (242.30–390.18)
Type A/B hospital	372.99 (236.69–509.30)	39.05 (19.17–58.94)	35.18 (2.78–67.57)	75 (36–100)	447.22 (298.81–595.63)
Type C/D hospital	185.70 (145.21–226.20)	30.55 (19.04–42.06)	12.67 (5.01–20.33)	58 (26–90)	228.92 (190.01–267.83)
Not medically attended	0.93 (0.40–1.45)	3.36 (1.83–4.88)	3.19(0.00–7.05)	13 (0–28)	7.48 (2.36–12.60)
All (weighted average)†	37.35 (27.07–47.63)	7.72 (4.84–10.59)	5.34 (1.13–9.99)	23 (8–40)	50.41(35.75–65.07)

* % Positive indicates the proportion of respondents in each setting who lost income, that is, with positive (nonzero) indirect costs.

† The central estimates of the number of nonfatal cases by setting from Table 3 are used as the weights for combining settings here and in Table 3. The lower and upper bound estimates are the weighted averages of the lower and upper bounds of cost per episode in each setting, a procedure which provides the widest possible range. The range in parentheses below each entry is its 95% uncertainty interval.

### Aggregate dengue costs.

Based on O’Reilly et al.,^25^ we estimate that there were about 7.5 million dengue cases nationally in 2017, with 3,658 fatalities. In Yogyakarta city alone, the estimated cases were about 21,000 with about 12 fatal cases. Based on SUSENAS 2014,^15^ we estimate that 63% (nationally) and 48% (Yogyakarta city) of all dengue cases were not medically attended cases. The aggregate economic burden of dengue in 2017 was $681.26 (95% UI: $232.28–$2,371.56) million nationally and $2.46 (95% UI: $0.58–$36.35) million in Yogyakarta city alone. About 44% of the national economic burden came from the fatal cases (see Table 3).

**Table 3:** Estimated number of dengue cases, aggregate economic burden, and cost per episode in Indonesia by outcome and setting in 2017

	Nonfatal cases			Fatal cases		
Location	Hospitalized	Ambulatory	Not medically attended	Children	Adults	Total
Estimated number of cases						
National	963,894 (182,172–2,034,019)	1,720,254 (26,229–3,949,950)	4,847,450 (1,108,670–10,520,849)	1,317 (572–2,966)	2,340 (1,016–5,270)	7,535,256 (1,318,658–16,513,055)
Yogyakarta city	3,908 (686–12,886)	7,195 (0–27,319)	10,332 (2,186–36,220)	4 (1–14)	8 (2–25)	21,447 (3,416–76,005)
Estimated aggregate economic burden of dengue (US$ millions)						
National	304.82 (89.12–1,042.56)	38.61 (3.05–488.32)	36.25 (8.91–147.44)	139.95 (60.88–321.71)	161.63 (70.31–371.53)	681.26 (232.28–2,371.56)
Yogyakarta city	1.24 (0.28–5.46)	0.16 (0.00–27.09)	0.08 (0.02–0.40)	0.46 (0.13–1.58)	0.53 (0.15–1.82)	2.46 (0.58–36.35)
Estimated cost per episode (US$)						
National	316.24 (242.30–390.18)	22.45 (14.12–30.77)	7.48 (2.36–12.60)	106,247.41 (98,374.05–125,622.30)	69,060.81 (63,943.13–81,654.49)	90.41 (72.79–112.35)

Costs of nonfatal cases were derived as explained in notes to Table 2. Costs per episode of fatal cases were adjusted from those reported by Shepard et al.^1^ using the change in Indonesia’s per capita gross national income. Numbers in parentheses are 95% uncertainty intervals.

**Figure 2.**
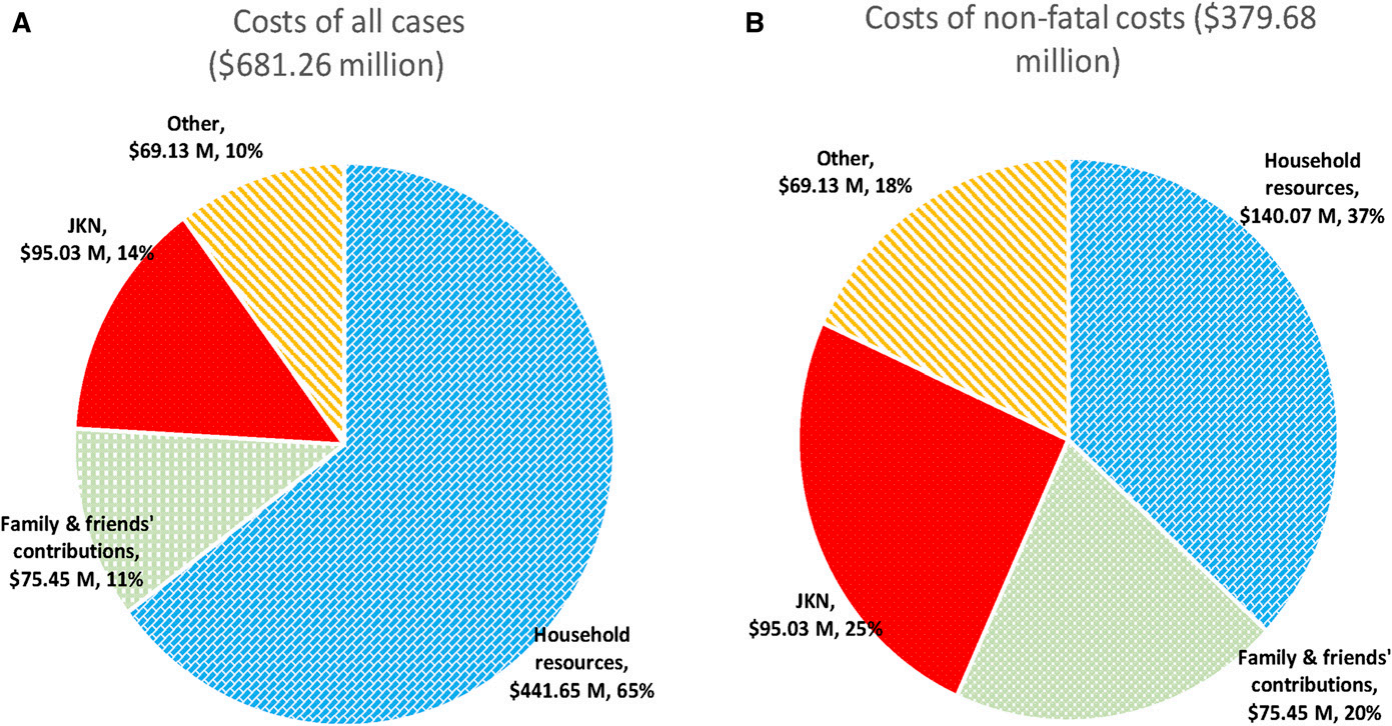
Distribution of financing of costs of all and nonfatal dengue cases in 2017 US$. JKN = Jaminan Kesehatan Nasional. This figure appears in color at www.ajtmh.org.

Among the nonfatal cases, slightly more than half of the overall cost was covered by households. However, about one-third of household expenses for the dengue episode were offset by contributions or gifts from family and friends. These contributions represented 47% of household expenses for hospitalized cases, 6% for ambulatory, and 9% for self-treated cases. The other half of the dengue cost was covered by JKN, subsidies from governments and nonprofit organizations, other resources in healthcare facilities, and private insurance (see Figure 2). These government subsidies include national health operational subsidies (the *Bantuan Operasional Kesehatan* [BOK]) program,^11^ disease-specific medicines and health equipment, and salaries for the civil servants (doctors, nurses, and other medical and administrative workers; see Supplemental Table S7).

More specifically, this study found that JKN covered about one-fourth of the economic burden of nonfatal dengue (25% nationally and 26% for Yogyakarta city). About 18% (nationally) and 19% (Yogyakarta city) of the costs were covered through other subsidies, including other government programs and private donations. This means that the percentage of dengue cost funded outside of the household reached 43% nationally and 45% in Yogyakarta city. Jaminan Kesehatan Nasional funded a greater share for hospitalized cases (30% of total economic burden and 60% of non-household payments) than for ambulatory episodes (11% of total economic burden and 37% of non-household payments). In total, JKN paid about $95.03 (95% UI: $26.84–$365.98) million nationally and $0.386 (95% UI: $0.083–$4.738) million in Yogyakarta city to cover dengue costs in 2017.

**Figure 3.**
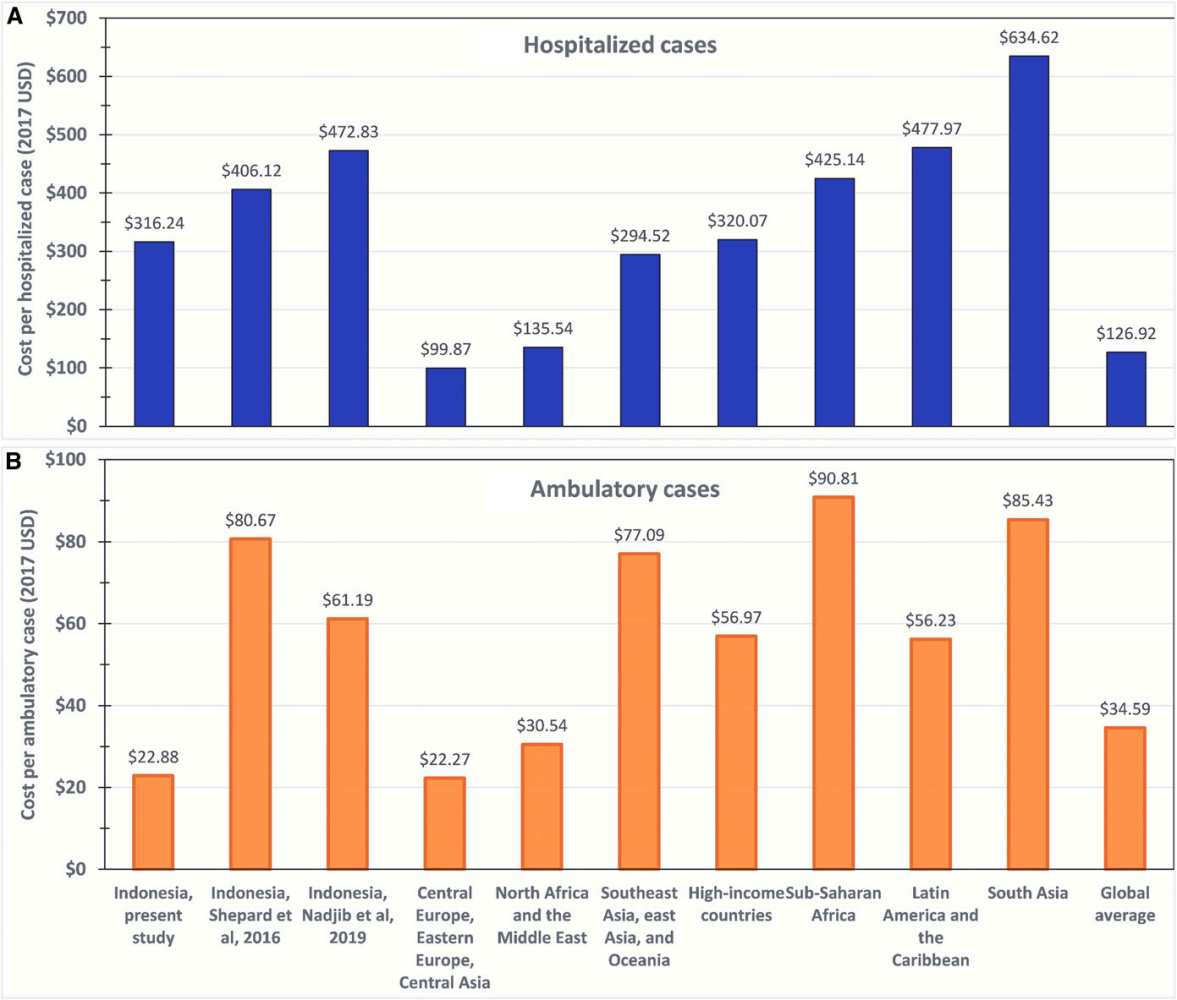
Comparative adjusted dengue cost per episode (in 2017 US$) by region, and data source in hospitalized (**A**) and ambulatory (**B**) settings. Note: Originally published costs were adjusted based on the gross national income (GNI) per capita for the original study setting and year, compared with the GNI per capita for Indonesia for 2017, in US$ at market prices. For details, see Supplemental Appendix 1. Sources: Breakdown by region^1^ and Indonesia (this study and Nadjib et al.^27^). Averages for Nadjib et al.^27^ were derived as arithmetic averages of the site-specific values reported by the original authors. This figure appears in color at www.ajtmh.org.

Figure 3A shows that our estimate of the cost per hospitalized case (US$316.24) was lower than the other two published estimates for Indonesia, those by Shepard^1^ and Nadjib et al.,^27^ shown in the next two bars. Similarly, Figure 3B shows that our estimate of the cost per ambulatory case ($22.45) was also lower than the other two estimates for Indonesia. In fact, our estimate was only about a quarter of that from Shepard^1^ (see Supplemental Table S6). Their value of $17.54 for hospital ambulatory direct cost is the arithmetic average of their values for ambulatory cases in Yogyakarta’s public and private hospitals of $7.57 and $27.51, respectively.

Figure 3A also shows that compared with various regional averages, the cost of a hospitalized episode in this study falls toward the middle of the range from other regions adjusted for economic conditions (the next seven bars). Three regions are lower, whereas four regions are higher. The value for Indonesia in this study is quite close to the average for the region of Southeast Asia, East Asia, and Oceania (US$294.52). However, when this study’s estimate of the cost of an ambulatory episode is compared with that from other regions, Indonesia is nearly the lowest. Even after our adjustment for economic conditions, the variability among regions is substantial. Compared with the global average, Indonesia is higher for hospitalized but lower for ambulatory episodes.

## DISCUSSION

This study found that the per-case cost for a nonfatal dengue case averaged $22.45 (95% UI: $14.12–$30.77), $316.24 (95% UI: $242.30–$390.18), $7.48 (95% UI: $2.36–$12.60), and $50.41 (95% UI: $35.75–$65.07), respectively, for ambulatory, hospitalized, not medically attended, and overall settings. Including fatal cases, the overall cost per case average became $90.41 (95% UI: $79.27–$112.35). Using 2017 regional and national estimated cases, this study then found that the aggregate economic burden of dengue was $681.26 million (95% UI: $232.28–$2,371.56 million) nationally and $2.46 million (95% UI: $0.58–$36.35 million) in Yogyakarta city. About half of this burden was paid by households (including friends and relatives of the patient), about one-fourth by the JKN program, and the remainder from other public and philanthropic subsidies.

We believe this study has extended the procedures for calculating the economic cost of dengue generally, and in Indonesia specifically, in several ways. First, it is the first in Asia and only the second globally (after research in Morelos, Mexico^28^) to include not medically attended cases based on empirical estimates. In our study, the share of such cases was based on a large, national household survey conducted by SUSENAS.^15^ The unit cost was based on interviews with a validated questionnaire of patients across several communities in Yogyakarta city.

Second, the study obtained empirical cost data from representative institutions at diverse levels of the healthcare system. Indonesia categorizes hospitals by type, from type A (with the widest range of services) to type D (with the narrowest range). The major distinction is between the advanced hospitals (types A and B), for which referral is generally required for admission under JKN, and the regular hospitals (types C and D), which transfer out their most severe dengue cases. Empirical studies on the burden of dengue, which generally involve interviewing patients, are commonly conducted by researchers at type A or B hospitals and enroll patients from these types. The resulting data tend to reflect illness at the upper end of severity and sophistication. As our study identified hospitalized patients through the surveillance system, it identified and studied patients in both the higher and lower categories of hospitals and should not be subject to the selection effects of much of the existing research.

Third, our estimates of direct medical costs were based on actual operational costs of the health facilities, rather than bills to patients or insurers or charges. Because many public and voluntary health facilities receive subsidies and charitable donations, they do not need to try to recover all of their costs from fees, so patients’ bills tend to underestimate the economic costs. We checked that the consistency of our estimated costs per episode was reasonable by comparing their relationship with the relative reimbursements from JKN (which was based on national costs). The ratio of this cost in our type D hospital to that in our type B facility was 0.47. For the relevant diagnosis category “other viral and nonbacterial infection” for medium severity in a class II bed in a government hospital, the relative reimbursement from JKN was 0.54.^23^ The similarity of these ratios supports the generalizability of our findings to dengue hospitalizations overall.

Fourth, unrecognized and unreported apparent dengue virus infections make it difficult to estimate the true extent of dengue illness.^1^ Responding to this challenge, our estimates of numbers of symptomatic dengue cases were based on a comprehensive, recently published, mathematical model calibrated with surveillance data from Indonesia.^25^

Fifth, our estimates of indirect costs were based on interviews with patients and their families estimating their actual losses. We valued part of the economic costs of informal caregivers, including family members and relatives, by asking their income loss during the patient’s episode of the illness. The estimates incorporated the household’s report of sick leave and other adjustments, rather than an assumed value. The percentage of respondents reporting income loss increased steadily with the intensity of the setting, rising from 13% in not medically attended episodes to 75% in type A/B hospitals. These results suggest that the responses were plausible.

Sixth, this research offers a detailed breakdown by source of financing. To our knowledge, this is the first study of financing of dengue illness in a low- or middle-income country with a national health insurance system, and only the second study of dengue financing, after one in Puerto Rico, anywhere.^29^ This breakdown was informed both by interviews with patients and their household members (to obtain household expenditures), as well as systematic analysis of the finances of selected health facilities. By triangulating between facility and household data, this study improved the accuracy of financing breakdowns. By including semi-structured interviews with household members, this study also identified subtleties in household contributions, such as gifts to a household from family and/or friends, particularly in connection with a hospitalization.

This study’s financing breakdown helps inform multiple policy-makers on the potential economic benefits of implementing more effective programs of dengue prevention. Overall, this study discovered that about 25% of dengue nonfatal burden has been covered by JKN and nearly 20% by other subsidies that mostly came from the government (which supports the many public healthcare facilities). These national aggregates were about $95.03 million for JKN and $69.13 million for other subsidies in 2017. The cost to JKN alone was about 13% of 2017 JKN’s deficit of $728.65 million. Thus, a successful dengue prevention strategy, averting many hospitalizations, would help to address the national insurer’s deficit.

In view of these refinements, it is instructive to compare this study with previous research. The most thorough previous study was by Nadjib et al.,^27^ for which adjusted values were included in Figure 3. Because of differences in methods and data sources, Nadjib et al.^27^ estimated higher costs per case for both ambulatory and hospitalized patients. However, we estimated a substantially higher number of cases, included fatal cases, and found higher annual aggregate costs.

The first major methodological difference between this study and that of Nadjib et al.^27^ concerned the method for estimating indirect cost. Whereas Nadjib et al.^27^ used imputed values, our study relied on respondents’ answers about their potential income loss related to the dengue episode. In our study, some formal-sector respondents received paid sick leave, experiencing at most a loss of attendance or lunch allowances equal to about a quarter of the take-home pay. Many informal-sector workers experienced no financial loss, as coworkers or extended family members filled in for their absence. Other respondents noted that they did not experience any income loss because they were pensioners or no longer in the labor force. By contrast, Nadjib et al.^27^ imputed a loss of income to all adult respondents, either as a patient or caregiver. For most adults, they valued this approach at the regional minimum wage. In Yogyakarta city, for example, this was Rp 1,572,200 (US$117.50) per month in 2017.^30^ At about 22 working days per month, this was Rp 71,463 (US$5.34) per day. As informal-sector workers were not subject to the minimum wage, their losses, even where they occurred, would likely have been less than the minimum wage. All of these factors led to Nadjib et al.’s^27^ higher indirect cost per case compared with our study’s estimates.

The second major methodological difference was their assumption that all ambulatory cases received care in the formal medical system, thus incurring that system’s direct costs. The third methodological difference was that Nadjib et al.^27^ addressed only nonfatal cases, whereas this study also included fatal cases.

Nadjib et al.’s^27^ estimates of numbers of cases were based on the surveillance system, whereas ours were based on the model by O’Reilly et al.^25^ and empirical allocations among sectors. For hospitalized cases, our study estimated 963,894 compared with 898,475 for Nadjib et al.^27^ For formal ambulatory cases, the corresponding estimates were 1,720,254 and 596,391, respectively. Finally, our study estimated 4,847,450 not medically attended and 3,657 fatal cases, whereas Nadjib et al.^27^ excluded both of these groups. Overall, this study found aggregate costs ($681.26 million) were 73.2% higher than that in the most recent previous study ($393.38 million).^27^

The next relevant comparison concerns the Indonesia-specific findings in a global study.^1,27^ Incorporating our adjustment to 2017, the present study found lower costs per episode in all three settings than the 2016 study.^1^ Specifically, the ambulatory direct costs (medical and nonmedical) per case for this study and Shepard^1^ are $20.20 and $50.30, and $294.57 and $311.29 for hospitalized, and $4.29 and $9.49 for not medically attended cases, respectively. These differences reflect the refinement of having actual, country-specific data in the present data. Finally, aggregate costs in the present study ($681.26 million) were less than a third of the estimate from Shepard^1^ ($2,195 million in 2013 prices).

The similarity in cost per hospitalized episode between this study and those concerning the broader region of Southeast Asia, East Asia, and Oceania suggests that Indonesia’s management and duration of hospitalized episodes are likely comparable to those of its regional neighbors. Regular international professional exchanges, such as the annual Dengue Summit (with the 2019 summit in Jakarta), help exchange best practices across the region. A number of factors might explain Indonesia’s lower cost per ambulatory episode: its well-developed network of puskesmas and evening access to ambulatory care (most individual practitioners and private clinics operate to around 10 pm) reduce indirect costs, whereas providers’ tendency to hospitalize many cases leads to a low severity for those cases remaining in the ambulatory sector.

Several limitations should be acknowledged, although our analysis suggests that none created a major bias. First, our original data came from only one city, Yogyakarta. However, unit costs of medical services are guided substantially by national government policies on operational subsidies to health facilities (the BOK program) and JKN reimbursement rates. Our study further minimized this potential problem by studying several institutions spanning multiple levels (A through D) and sectors (public and private), as also performed by Nadjib et al.^27^ Differences between Yogyakarta and national average likely had offsetting effects. On the one hand, Yogyarkarta’s share of febrile illness that was medically attended (52%) exceeded the national average (37%),^15^ thereby raising Yogyakarta’s direct cost per case greater than the national average. On the other hand, whereas Yogyakarta’s 2017 GDP per capita was only 0.45 times the national average, dengue cases tend to be concentrated in major Indonesian cities. Their GDPs per capita all exceed the national averages, with Jakarta, Surabaya, and Medan having multiples of 3.14, 2.33, and 1.23 times, respectively. Indirect cost per case is roughly proportional to per capita GDP. Thus, Yogyakarta’s indirect cost per case was less than the national average. Therefore, overall dengue cost information from Yogyakarta should be nationally representative.

Second, our sample of hospitals was only two hospitals. Although they came from different types of hospitals (B and D), they still covered only two of the four types. Although this small sample opens the possibility for random variation, it does not create bias. The generalizability of our results is further strengthened by our inclusion of private not-for-profit hospitals.

Third, our estimates of the cost of a not medically attended case were based on interviews with patients experiencing febrile illness consistent with dengue, but without a means for laboratory confirmation. Prospective studies, however, have confirmed that dengue is responsible for a notable share of febrile illness in Indonesia,^26^ and households treat any febrile illness, especially in children, with the possibility that it could be dengue. In addition, our recruitment of not medically attended cases through community meetings could not ascertain whether there were other persons who may have had such cases but declined to discuss them, and, if so, whether they differed systematically from the respondents. However, our higher average age of not medically attended cases is consistent with the pattern from SUSENAS (2014), where the proportion of febrile cases that self-treat increases with age.^15^

Fourth, recall bias is always a concern in health expenditure studies and can lead to underestimates of numbers of visits, hospitalizations, other services, and their associated costs. However, this study minimized this concern for hospitalized and ambulatory cases because we had administrative records showing the actual dates of service and used a calendar during interviews.

Fifth, although we examined the relationship between lost time and lost income, we were not able to quantify all economic losses. Nevertheless, our descriptive results provided illustrations of time loss that did not result in measured income loss. For example, one mother described a cascade of actions to cope with her child’s illness. She devoted her full attention to caring for the sick child while her husband continued his paid employment. During the illness, an auntie came to the house and filled in for other household needs, such as cooking and caring for other children. During that period, the auntie had to forgo her usual activity as a home-based craftswoman making buttons from coconut shells. Based on follow-up communication, we inferred that she grossed about $1 per day from selling the buttons, but could not determine what she paid for inputs. The example demonstrates that this study’s reliance on lost income did not capture the full economic loss of a dengue episode, and, if the auntie were not living nearby, the income loss could have been much greater.

Sixth, our study design treated dengue as an acute episode and did not probe for possible long-term effects of persistent dengue. Recent research in other countries has shown that fatigue and other symptoms may persist for months beyond the acute phase.^31–33^ A literature-based assessment of the health burden from a dengue episode found median DALY burdens of 0.012 for the acute phase, 0.019 for the persistent phase, and 0.031 overall.^7^ Thus, because the persistent phase could last for months compared with days for the acute phase, the overall burden was 2.6 times that of the acute phase alone. However, research on agricultural workers showed that a partially debilitating illness did not necessarily lead to income loss as those affected worked harder to maintain their income.^34^

In conclusion, we believe that this study advances the literature on cost and burden of dengue generally, and in Indonesia specifically, in several ways. It found that dengue costs were 73% higher than previously estimated. The study offers several methodological advances. The most important is the empirically based inclusion of not medically attended (self-treated) dengue. In addition, it breaks down the costs of nonfatal dengue cases, showing that households bear about half the cost, whereas government and healthcare institutions bear the remainder. These results provide a robust empirical base for economic assessment of dengue technologies for the most populous country in Southeast Asia.

## Supplemental information, tables, and figures

Supplemental materials
