# Supplementary material for: Cost of Dengue Illness in Indonesia across Hospital, Ambulatory, and not Medically Attended Settings
Source: Am J Trop Med Hyg. 2020 Sep 8;103(5):2029–39. doi: 10.4269/ajtmh.19-0855 (PMC7646801; doi:10.4269/ajtmh.19-0855)
Supplement: Supplementary file 1 [file tpmd190855.SD1.pdf]

Supplementary Information

Cost of dengue illness in Indonesia across hospital, ambulatory and non-medical settings

Nandyan N. Wilastonegoro, Dinar D. Kharisma, Ida S. Laksono, Yara A. Halasa-Rappel, Oliver J. Brady, Donald S. Shepard

Contents

S1: Questionnaire S1. Adult questionnaire in English

S2: Questionnaire S2. Adult questionnaire in Bahasa Indonesia

S3: Questionnaire S3. Pediatric questionnaire (patients under age 18) in English

S4: Questionnaire S4. Pediatric questionnaire (patients under age 18) in Bahasa Indonesia

S5: Table S5. Studies on cost of a dengue episode in the literature by country

S6: Table S6. Studies on cost of a dengue episode in the literature by region

S7: Table S7. Dengue economic burden by type of case and source of financing (in millions of 2017 US$)

S8: Figure S8. Histogram of direct medical cost per hospitalized case in Yogyakarta, Indonesia (n=20)

S9: Figure S9. Histogram of non-medical cost per hospitalized case in Yogyakarta, Indonesia (n=20)

S10: Figure S10. Histogram of indirect cost per hospitalized case in Yogyakarta, Indonesia (n=20)

S11: Figure S11. Histogram of total cost per hospitalized case in Yogyakarta, Indonesia (n=20)

Supplementary Information

Cost of dengue illness in Indonesia across hospital, ambulatory and non-medical settings

Questionnaire Contents

S1: Questionnaire S1. Adult questionnaire in English

S2: Questionnaire S2. Adult questionnaire in Bahasa Indonesia

S3: Questionnaire S3. Pediatric questionnaire (patients under age 18) in English

S4: Questionnaire S4. Pediatric questionnaire (patients under age 18) in Bahasa Indonesia

Supplementary Information

Cost of dengue illness in Indonesia across hospital, ambulatory and non-medical settings

S1: Questionnaire S1. Adult questionnaire in English

Adult Confirmed Dengue Patient Questionnaire (Age 18+)

Patient name ______________
Patient study ID ______________
Date of interview ______________

Interviewer

Q0. *The name of the site the patient was recruited from. (Choose one)*

1 Hospital ____________
2 Health center ____________
3 Outside professional health system through (name of community health worker) ________________________

Q00. *Interviewee*

1 Patient him/herself
2 Patient proxy (go to Child questionnaire)

***READ**:* Now we are going to talk about your illness period. I will give you a calendar that will help you answer the following questions.
Show calendar flashcard (form 1) to the patient and continue to use through Q15. Write "today" in the box for today's date.

Q1. When did you start feeling ill?
Write "Illness started" on that date in the calendar.

__/__/____	dd/mm/yyyy
01/01/2097	Don't Know
01/01/2098	Refuse to Answer

Q2. Have you had fever during this illness episode that started in [date in Q1]?

1	Yes	
0	No	***Skip to Q6***
8	Refuse to Answer	***Skip to Q6***

Q.3 When did your fever start?
Write "fever started" on that date in the calendar,

__/__/____	dd/mm/yyyy
01/01/2097	Don't Know
01/01/2098	Refuse to Answer

Q.4 Did your fever end?

1	Yes	
0	No	***Skip to Q6***
8	Refuse to Answer	***Skip to Q6***

Q.5 When did your fever end?
Write "fever ended" on that date in the calendar,

__/__/____	dd/mm/yyyy
01/01/2097	Don't Know
01/01/2098	Refuse to Answer

Q6. What other symptoms did you have?

1 Myalgia
2 Nausea
3 Headache
4 Others

Q6a Other Symptoms ____
____
____

Q7.	Over your entire illness, on which day did you feel worst?<br>*Write "worst day" on that date in the calendar,*	__/__/____<br>01/01/2097<br>01/01/2098	dd/mm/yyyy<br>Don't Know<br>Refuse to Answer	
Q8.	At the time of this interview, are you still feeling ill?	1<br>0	Yes<br>No	***Skip to Q11***
Q9.	Have you recovered completely from this illness period?	1<br>0<br>8	Yes<br>No<br>Refused to Answer	 ***Skip to Q11***<br>***Skip to Q11***
Q10.	When did you recover completely from the illness episode?<br>*Write "recovered completely" on that date in the calendar,*	__/__/____<br>01/01/2097<br>01/01/2098	dd/mm/yyyy<br>Don't Know<br>Refuse to Answer	

Q11. How soon after you started feeling ill did you first receive care by a medical care provider? (Choose one)

1	Within 24 hours
2	Between 24 and 48 hours
3	After 48 hours
7	Don't Know
8	Refused to Answer

Care received by patient from the day he/she started feeling ill until today

NOTE to interviewer: if you have forms from hospital please fill Q12 and 13 from the hospital form

Q12. Is the hospital where the patient was admitted a public, social-private, or private?

1 Public
2 Social-private
3 Private
7 Don't Know
8 Refuse to Answer

Q13. Indicate the type of hospital in which you spent the most number of nights?

1 University, tertiary or referral hospital
2 Other hospital
7 Don't Know
8 Refuse to Answer

Hospitalization

READ: In the next set of questions I would like to know whether you have spent one or more nights in a hospital.

Q14. Were you hospitalized during this illness episode?

1 Yes
0 No ***Skip to instruction before Q26***
8 Refused to answer ***Skip to instruction before Q26***

Q14a. When were your admitted to the hospital __/__/____ dd/mm/yyyy
Write "admission" on that date in the calendar, 01/01/2097 Don't Know
01/01/2098 Refuse to Answer

Q14b. During this hospitalization, for how many nights did you stay in the hospital? __ __ . __ __
98 Refuse to Answer

Q14c. When were your discharged from the hospital __/__/____ dd/mm/yyyy
Write "discharged" on that date in the calendar, 01/01/2097 Don't Know
01/01/2098 Refuse to Answer

READ: Now I would like to ask you some questions on the duration of your hospitalization, the out-of-pocket payments you made for treatment and other spending related to this hospitalization.

Q15. Can you please tell me which type of hospital ward-class did you stay at for the majority of your hospital stay?

1 VIP
2 Class 1
3 Class 2
4 Class 3

Q16. During your hospitalization, did you stay any night in an intensive care unit?

1	Yes	
0	No	***Skip to instruction before Q18***
8	Refuse to Answer	***Skip to instruction before Q18***

Q17. For how many nights did you stay in the intensive care unit? (if patient stayed in the intensive care unit for one night or less then mark "1".

__ __ . __ __

98 Refuse to A

READ: Now I will ask about the out-of-pocket payments you made in this facility.
Your out-of-pocket payments are the total amounts paid by you or your family for your treatment, whether or not you received reimbursement for all or part of this paid amount during this illness episode.

Q18. Please report your best estimate of the total out-of-pocket payment that you have paid up to today for services delivered in during your hospitalization? (Round all items to the nearest IDR). If there were no payments, please enter "0".
Please include both payments already made and those that are expected to be made for the services received.

__ __ __ __ __ __ __

7	Don't Know	***Skip to instruction before Q23***
8	Refuse to Answer	***Skip to instruction before Q23***

Q19. Can you break down these payments by type of service received during your stay in the hospital?

1	Yes	
0	No	***Skip to instruction before Q23***
7	Don't Know	***Skip to instruction before Q23***
8	Refuse to Answer	***Skip to instruction before Q23***

Q20. How much did your household pay out-of-pocket for consultation, admission and discharge fees?
If no spending put "0".

__ __ __ __ __ __ __ __

7	Don't Know
8	Refuse to Answer
9	Not Applicable

Q21. How much did your household pay out-of-pocket for diagnostic tests such as blood tests, dengue test, x-rays or sonogram?
If no spending put "0".

__ __ __ __ __ __ __ __

7	Don't Know
8	Refuse to Answer
9	Not Applicable

Q22. How much did your household pay out-of-pocket on all treatment such as medications, intravenous fluid (IVF), medical equipment (I/V sets) or blood bank.
If no spending put "0"

__ __ __ __ __ __ __ __

7	Don't Know
8	Refuse to Answer
9	Not Applicable

READ: Now I would like to ask you questions about other spending related to your illness during this illness episode from the day you started feeling ill [Response to Q1] until today, such as spending on transportation, meals or lodging. Include also any expenses by any other household member (person you live with), other family members or friend who accompanied you to the hospital for care.

Q23a. Can you please tell me the type of transportation you used to go to the hospital?

____

Note to interviewer: if patient used household own car or motorcycle then, ask about the distance from the patient's house to the hospital. If the patient doesn't know the answer then use google map to determine the distance.

Q23b. Please report your best estimate of your household out of pocket spending on transportation to the hospital, including ambulance.
If no spending put "0".
If family or owned vehicle, estimate mileage traveled (two ways travel) and use IDR 16,000 per Km as a proxy for the cost of driving. If family or patient owned Auto-rickshaw/own scooter use IDR 14,000 per Km. If no spending record "0.00".

__ __ __ __ __ __ __ __ __

7	Don't Know
8	Refuse to Answer
9	Not applicable

Q23c. Can you please tell me the type of transportation you used to get back from the hospital?

____

Note to interviewer: if transportation used was a car, ask about the distance from hospital to patient's house.

Q23d. Please report your best estimate of your household out of pocket spending on transportation from the hospital, including ambulance.

If no spending put "0".

If family or owned vehicle, estimate mileage traveled (two ways travel) and use IDR 16,000 per Km as a proxy for the cost of driving. If family or patient owned Auto-rickshaw/own scooter use IDR 14,000 per Km. If no spending record "0.00".

__ __ __ __ __ __ __ __ __

7 Don't Know

8 Refuse to Answer

9 Not Applicable

Q24. Please report best estimate of your household out-of-pocket spending on food or meals.

If no spending put "0" __ __ __ __ __ __ __ __ __

7 Don't Know

8 Refuse to Answer

9 Not Applicable

Q25. Please report best estimate of your household out-of-pocket spending on lodging in a non-health facility such as a hotel.

If no spending put "0" __ __ __ __ __ __ __ __ __

7 Don't Know

8 Refuse to Answer

9 Not Applicable

Ambulatory care

READ: These next questions are about any ambulatory health care sought or received since you started feeling ill on [Response to Q1] until today. Please, ***include*** care from all types of health facilities such as pharmacy, traditional healer, private clinic, Puskesma primary health center, midwife, hospital outpatient department and/or emergency room, and private lab.

Q26. Did you visit any ambulatory health facility during your latest dengue episode? (Show flash card Form 1).

1	Yes	
0	No	***Go to Q40***
8	Refuse to Answer	***Go to Q40***

READ: Think about each time you had contacted with one of those health facilities during the dengue episode that started in [date in Q1]. Then specify the type of care you received at each of the health facilities you visited (consultations, diagnostic tests and/or treatment) and try to remember the amounts your household, other family members or friend paid out of pocket in total or for each type of service received.

Q27. How many visits have you had to any of the following health facilities during this interview period? ***Show flashcard forms 1 (calendar) and 2 (List of healthcare providers)***

Visit	Number of visits
Pharmacy (01)	
Traditional healer (02)	
Private clinic (03)	
Puskesmas Primary health center (04)	
Midwife (05)	
Outpatient department in a hospital (06)	
Emergency room (07)	
Private lab (08)	
Satellite health center (09)	
Other (10)	
Specify others_______________	

Q28. Choose from the following the first *(second, third,..)* type of health facility you contracted during this interview period. Can you give the date of each visit? (*Show flashcard form 2 and form 1 (the calendar*) (Choose one for each visit)

Visit	First	Second	Third	Fourth	Fifth	Sixth
Date						
Type of facility						
Pharmacy (01)						
Traditional healer (02)						
Private clinic (03)						
Puskesmas Primary health center (04)						
Midwife (05)						
Outpatient department in a hospital (06)						
Emergency room (07)						
Private lab (08)						
Satellite health center (09)						
Other (10), Specify__________						
Don’t know (97)						

Refuse to answer (98)						

Read: What is the name of the facility you went to? (Fill in the space next to type of facility the patient chose)

Note for interviewer: A "Public" Facility is a health facility operated by any level of government, and a "social-private" facility is organization run by private non-profit organization, Private is an individual doctor or group of private doctors.

Q29. What type of facility did you visit, during your X visit? Was it public or non-public facility? (Choose one per visit)

Visit	Public (1)	Social-private (2)	Private (3)	Don't know (7)	Refuse to answer (8)
First					
Second					
Third					
Fourth					
Fifth					
Sixth					

Q30. What kind of service did you receive in this facility during your X visit? (Check all that apply)

Visit	Consultations	Diagnostic test such as x-ray, blood test	Treatment such as medications, intravenous fluid	Referral	Don't know (7)	Refuse to answer (8)
First						
Second						
Third						
Fourth						
Fifth						
Sixth						

READ: Now I would like to ask you about the out-of-pocket payments your household, other family members or friend, made in this facility.
Note for interviewer: *Whenever possible, cross check the amount with bills available from the health facility.*

Q31. Please report your best estimate of the total out-of-pocket payment for services received in this facility? Please include both payments already made and those that are expected to be made for the services received.
If there were no payments, please enter "0.00".

Visit	Amount, IDR	***If zero skip to instruction before Q36a***	Don't know (7)	***Skip to instruction before Q36a***	Refuse of answer (8)	***Skip to instruction before Q36a***
First						
Second						
Third						
Fourth						
Fifth						
Sixth						

Q32. Can you break down the total payments by type of service received?

Visit	Yes (1)	No (0)	***Skip to instruction before Q36a***	Refuse to answer (8)	***Skip to instruction before Q36a***	Not applicable (9)	***Skip to instruction before Q36a***
First							
Second							
Third							

Fourth							
Fifth							
Sixth							

Q33. Out-of-pocket payment on: Consultation. (*If no payment record "0.00")*

Visit	Amount, IDR	Don't know (7)	Refuse to answer (8)	Not applicable (9)
First				
Second				
Third				
Fourth				
Fifth				
Sixth				

Q34. Out-of-pocket payment on: diagnostic tests or blood bank such as blood test, dengue test, x-rays, sonogram... *If no payment record "0.00".*

Visit	Amount, IDR	Don't know (7)	Refuse to answer (8)	Not applicable (9)
First				
Second				
Third				
Fourth				
Fifth				
Sixth				

Q35. Out-of-pocket payment on: treatment such as medications, medical equipment (intravenous sets). *If no payment record "0.00".*

Visit	Amount, IDR	Don't know (7)	Refuse to answer (8)	Not applicable (9)
First				
Second				
Third				
Fourth				
Fifth				
Sixth				

READ: Now I would like to ask you questions about other spending your household, other family members or friend, made specific for this facility during this illness episode such as spending on transportation, meals and lodging. Include also any expenses by any other household member who accompanied you to this first (*second, third,..*) facility for care.

Q36a. Can you please tell me the type of transportation you used to go to the [TYPE of health facility]?

Note to interviewer: if transportation used was household own car or motorcycle , ask about the distance from the patient's house to the [health facility].

Visit	Transportation type	Distance, KM
First		
Second		
Third		
Fourth		
Fifth		
Sixth		

Q36b. Please report the best estimate of out-of-pocket spending on transportation to the [TYPE of health facility]

If family or owned vehicle, estimate mileage traveled (two ways travel) and use IDR 16,000 per Km as a proxy for the cost of driving. If family or patient owned Auto-rickshaw/own scooter use IDR 14,000 per Km. If no spending record "0.00".

Visit	Amount, IDR	Don't know (7)	Refuse to answer (8)	Not applicable (9)
First				
Second				
Third				
Fourth				
Fifth				
Sixth				

Q36c. Can you please tell me the type of transportation you used to get back home from the [TYPE of health facility]?

Note to interviewer: if transportation used was a car, ask about the distance from [health facility] to patient's house.

Visit	Transportation type	Distance, KM
First		
Second		
Third		
Fourth		
Fifth		
Sixth		

Q36d. Please report the best estimate of out-of-pocket spending on transportation from the [Type health facility] to your home

If family or owned vehicle, estimate mileage traveled (two ways travel) and use IDR 16,000 per Km as a proxy for the cost of driving. If family or patient owned Auto-rickshaw/own scooter use IDR 14,000 per Km. If no spending record "0.00".

Visit	Amount, IDR	Don't know (7)	Refuse to answer (8)	Not applicable (9)
First				
Second				
Third				
Fourth				
Fifth				
Sixth				

Q37. Please report the best estimate of out-of-pocket spending on food or meals.

If not spending report "0.00"

Visit	Amount, IDR	Don't know (7)	Refuse to answer (8)	Not applicable (9)
First				
Second				
Third				
Fourth				
Fifth				
Sixth				

Q38. Please report the best estimate of out-of-pocket spending on lodging in a non-health facility such as a hotel.

If not spending report "0.00"

Visit	Amount, IDR	Don't know (7)	Refuse to answer (8)	Not applicable (9)
First				
Second				
Third				
Fourth				
Fifth				

Sixth				

Q39. Any other health facilities visited during this illness period from the day you stated feeling ill [Response to Q1] until today?

1 Yes ***Repeat the process starting with Q28 through Q39***
0 No

<u>Illness impact on household members, other family members who reside outside your household or friends during this illness episode</u>

READ: I would like to learn about how your recent dengue illness that occurred in [Date] have affected you and each of the members of your household**,** other family members who reside outside your household or friends.

READ: Let us talk about you first.

<u>The patient</u>

Q40. *Please record the sex of the patient without having to ask him/her.*
1 Male
2 Female

Q41. What is your current age? _____
999 Don't Know
998 Refuse to answer

Q41a. What is your date of birth (day/month and year)?
__/__/____ dd/mm/yyyy
01/01/2097 Don't Know
01/01/2098 Refuse to Answer

Q42. What is the highest level of education you have completed? (Choose one)
Show Flashcard-Form 3

01 Illiterate
02 Just literate but no schooling
03 Attended school for at least one year
04 Pre-school
05 Primary school
06 Middle school
07 High school
08 Diploma
09 Graduate
10 Post graduate (Masters, PhD)
11 Professional degree
97 Don't know
98 Refuse to answer
99 Not applicable

Q43. Are you currently studying?
1 Yes
0 No ***Skip to Q46***
8 Refused to answer ***Skip to Q46***

Q44. Have you lost any days of school due to your illness?

1 Yes
0 No ***Skip to Q46***
8 Refused to answer

Q45. How many days of school did you miss due to your illness?
Put "0" if none

______
97 Don't know
98 Refuse to answer
99 Not applicable

Q46. Are you working for pay?

1 Yes
0 No ***Skip to Q51a***
8 Refused to answer

Q47. Have you lost any days of work due to your illness?

1 Yes
0 No ***Skip to Q51a***
7 Don't know ***Skip to Q51a***
8 Refused to answer ***Skip to Q51a***

Q48. How many days of work did you lose due to your illness?
Put "0" if none

______
97 Don't know
98 Refuse to answer
99 Not applicable

Q49. Did you receive any paid sick leave for work days off, in which your employer or some agency paid your salary while you were ill?

1 Yes
0 No ***Skip to Q51a***
7 Don't know ***Skip to Q51a***
8 Refused to answer ***Skip to Q51a***
9 Not applicable ***Skip to Q51a***

Q50. How many paid sick days did you receive for this illness?
Put "0" if none

______
97 Don't know
98 Refuse to answer
99 Not applicable

Q51a. Have you lost any income due to your illness?

1 Yes
0 No ***Skip to Q52***
7 Don't know ***Skip to Q52***
8 Refused to answer ***Skip to Q52***

Q51b. What was the amount of income you lost? (Please give your best estimate)

____

7	Don't know
8	Refused to answer
9	Not applicable

Q52. Did you receive any monetary or non- monetary assistance from family members or the community for this illness episode?

1	Yes	
0	No	***Skip to Q55***
8	Refused to answer	***Skip to Q55***

Q53. What is your best estimate of the value of the non- monetary assistance?

True 0 if no non-financial assistance.

Put "0" if none _____

97	Don't know
98	Refuse to answer
99	Not applicable

Note to interviewer: If don't know, write the type of non-monetary assistance received by patient and patient's family

____

____

Q54. What is the total amount in IDR of the monetary assistance?

Put "0" if none _____

97	Don't know
98	Refuse to answer
99	Not applicable

Number of household members

Q55. How many members live in your household? (Other then yourself?)

______
00 Zero ***End interview***
98 Refuse to answer

.

Q56. How many of these household members have been affected by your illness directly during this illness episode?
READ: Household members are people who live with you and share meals. A household member is affected if he or she spent more time or money than usual caring for you or lost work, income, or school due to your illness.

______
00 Zero ***End interview***
98 Refuse to answer ***End Interview***

READ: Now I would like to ask you some questions about this/these [Response to Q56] member/s of your household who has/have been affected by your illness.

Care-givers

Q57. What is the relationship of this (first, second,...) affected household member to you? (Choose one)
Show flashcard form 4

Relationship	Household member							
	First member	Second member	Third member	Fourth member	Fifth member	Sixth member	Seventh member	Eighth member
Mother (01)								
Father (02)								
Husband (03)								
Wife (04)								
Housemate (05)								
Sister (06)								
Brother (07)								
Son (08)								
Daughter (09)								
Grandmother (10)								
Grandfather (11)								
Uncle (12)								
Aunt (13)								
House help (14								
Baby sitter (15)								
Other (16) Specify__________								
Refuse to answer (98)								

Q58. What is [Household member]'s age?

Household member	Age in years	Don't know (997)	Refuse to answer (998)
First member			
Second member			
Third member			

Fourth member			
Fifth member			
Sixth member			
Seventh member			
Eighth member			

Q58. What was the highest level of education that [household member] completed? (Choose one)
Show flashcard-form 3

Relationship	Household member							
	First member	Second member	Third member	Fourth member	Fifth member	Sixth member	Seventh member	Eighth member
Illiterate (01)								
Just literal but no schooling (02)								
Attended school for at least one year (03)								
Pre-school (04)								
Primary school (05)								
Middle school (06)								
High school (07)								
Diploma (08)								
Graduate (09)								
Post graduate (Masters, PhD) (10)								
Professional degree (11)								
Don't know (97)								
Refuse to answer (98)								
Not applicable (99)								

Q59. Is [Household member] currently studying?

Household member	Yes (Code:1)	No (Code:2)	***Skip to Q62***	Don't know (Code:7)	***Skip to Q62***	Refuse to answer (Code:8)	***Skip to Q62***
First member							
Second member							
Third member							
Fourth member							
Fifth member							
Sixth member							
Seventh member							
Eighth member							

Q60. Has [Household member] lost any days of school due to your illness?

Household member	Yes (Code:1)	No (Code:2)	***Skip to Q62***	Don't know (Code:7)	***Skip to Q62***	Refuse to answer (Code:8)	***Skip to Q62***
First member							
Second member							
Third member							
Fourth member							
Fifth member							
Sixth member							

Seventh member							
Eighth member							

Q61. How many days of school did [household member] missed due to your illness?
Record "0" if none

Household member	Days of schooling lost	Don't know (97)	Refuse to answer (98)
First member			
Second member			
Third member			
Fourth member			
Fifth member			
Sixth member			
Seventh member			
Eighth member			

Q62. Is [Household member] working for pay?

Household member	Yes (Code:1)	No (Code: 0)	***If no skip to Q67a***	Don't know (Code:7)	***Skip to Q67a***	Refuse to answer (Code:8)	***Skip to Q67a***
First member							
Second member							
Third member							
Fourth member							
Fifth member							
Sixth member							
Seventh member							
Eighth member							

Q63. Has [Household member] lost any days of work due to your illness?

Household member	Yes (Code:1)	No (Code: 0)	***Skip to Q67a***	Don't know (Code:7)	***Skip to Q67a***	Refuse to answer (Code:8)	***Skip to Q67a***
First member							
Second member							
Third member							
Fourth member							
Fifth member							
Sixth member							
Seventh member							
Eighth member							

Q64. How many days of work did [Household member] lose due to your illness?
Record "0" if none

Household member	Number of days	Don't know (Code:97)		Refuse to answer (Code:98)		Not applicable (Code:99)	
First member							
Second member							
Third member							
Fourth member							
Fifth member							
Sixth member							

Seventh member							
Eighth member							

Q65. Did [household member] receive any paid sick leave for work days off, in which the employer or some agency paid his/her salary for caring for you while you were ill?

Household member	Yes (Code: 1)	No (Code: 0)	***Skip to Q67a***	Don't know (Code:7)	***Skip to Q67a***	Refused to answer (Code:8)	***Skip to Q67a***	Not Applicable (Code:9)	***Skip to Q67a***
First member									
Second member									
Third member									
Fourth member									
Fifth member									
Sixth member									
Seventh member									
Eighth member									

Q66. How many paid sick days did [household member] receive?

Household member	Number of paid sick days	Don't know (97)	Refuse to answer (98)
First member			
Second member			
Third member			
Fourth member			
Fifth member			
Sixth member			
Seventh member			
Eighth member			

Q67a. Has [Household member] lost any income due to your illness?

Household member	Yes (Code:1)	No (Code: 0)	***Skip to Q68***	Don't know (Code:7)	***Skip to Q68***	Refuse to answer (Code:8)	***Skip to Q68***
First member							
Second member							
Third member							
Fourth member							
Fifth member							
Sixth member							
Seventh member							
Eighth member							

Q67b. What was the amount of income [household member] lost due to your illness? (Please give your best estimate). *If no payment record "0.00".*

Household member	Amount, IDR	Don't know (7)	Refuse to answer (8)	Not applicable (9)
First member				
Second member				
Third member				
Fourth member				
Fifth member				
Sixth member				

Seventh member				
Eighth member				

Q68. Of the number of sick days during this illness episode that started on [date of illness till patient date of recovery or date of interview], what was the total number of days of additional care that [household member] provided to you?
If none, enter 0

Household member	Number of days	***If "Zero" skip to Q70***	Don't know (Code:97)	***Skip to Q70***	Refused to answer (Code:98)	**Skip to Q70**	Not Applicable (Code:99)	***Skip to Q70***
First member								
Second member								
Third member								
Fourth member								
Fifth member								
Sixth member								
Seventh member								
Eighth member								

READ: In the questions below, think about the daily number of additional hours of care that you have received from [household member x] during your illness. Additional hours refer to any number of hours above those that [household member x] usually spends with you. In estimating the time, please include time spent at home, at the health facility and during transportation to and from the health facility

Q69. During these [Response to Q68] days, on average what was the average number of additional daily hours [household member x] spent caring for you?
If none, enter 0

Household member	Average additional hours of daily care	Don't know (Code:97)	Refused to answer (Code:98)	Not Applicable (Code:99)
First member				
Second member				
Third member				
Fourth member				
Fifth member				
Sixth member				
Seventh member				
Eighth member				

Q70. Did [household member x] have additional spending because of your illness such as transportation, meals or lodging expenditures other than the expenses previously mentioned for visiting health providers?

Household member	Yes (Code:1)	No (Code: 0)	***Skip to next household member***	Don't know (Code:7)	***Skip to next household member***	Refused to answer (Code:8)	***Skip to next household member***	Not Applicable (Code:9)	***Skip to next household member***
First member									
Second member									
Third member									
Fourth member									
Fifth member									
Sixth member									

Seventh member									
Eighth member									

Q71. For this question please report the best estimate of the total amount of additional spending over these [Number of days reported in Q68] days not already included in one of the previous questions
If no payment record "0.00".

Household member	Amount, IDR	Don't know (7)	Refuse to answer (8)	Not applicable (9)
First member				
Second member				
Third member				
Fourth member				
Fifth member				
Sixth member				
Seventh member				
Eighth member				

Check Q56, if the answer is more than 1 then do repeat questions 57 through 71 for all the household members listed. If Q56 is equal to 1, then read the sentence below and end interview.

READ: Thank you so much for your participation! If you have any questions regarding this interview or the results of this study please contact xxx at [Name of institute] from xx at xxx

Supplementary Information

Cost of dengue illness in Indonesia across hospital, ambulatory and non-medical settings

S2: Questionnaire S2. Adult questionnaire in Bahasa Indonesia

Kuesioner untuk Pasien Dengue Dewasa Terkonfirmasi (Usia 18+)

Nama Pasien ______________
Nomor Sampel ______________
Tanggal Wawancara ______________

Wawancara

Q0. Nama fasilitas kesehatan tempat pasien dirawat (pilih satu)

1 Rumah Sakit ____________
2 Puskesmas ____________
3 Dari luar fasilitas kesehatan, melalui kader a.n. ________________________

Q00. *Yang diwawancara*

1 Pasien sendiri
2 Wali pasien (ganti ke kuesioner wali)

Bacakan*:* Sekarang kita akan berbicara mengenai periode penyakit Anda. Dan saya akan menunjukkan kalender yang akan membantu Anda menjawab pertanyaan berikut.
Tunjukkan kalender (form 1)ke pasien dan lanjutkan hinga Q15. Tuliskan "hari ini" pada kotak untuk tanggal hari ini.

Q1. Kapan Anda mulai measa sakit?
Tuliskan "mulai sakit" pada tanggal di kalender

__/__/____ dd/mm/yyyy
01/01/2097 Tidak tahu
01/01/2098 Menolak menjawab

Q2. Apakah Anda mengalami demam [merespons Q. 1]?

1 Ya
0 Tidak ***Lanjut ke Q6***
8 Menolak menjawab ***Lanjut ke Q6***

Q.3 Kapan Anda mulai demam?
Tuliskan "mulai demam" pada tanggal di kalender

__/__/____ dd/mm/yyyy
01/01/2097 Tidak tahu
01/01/2098 Menolak menjawab

Q.4 Apakah demam Anda saat ini telah berakhir?

1 Ya
0 Tidak ***Lanjut ke Q6***
8 Menolak menjawab ***Lanjut ke Q6***

Q.5 Kapan demam Anda berakhir?
Tuliskan "demam berakhir" pada tanggal di kalender

__/__/____ dd/mm/yyyy
01/01/2097 Tidak tahu
01/01/2098 Menolak menjawab

Q6. Gejala apa yang Anda rasakan?

1 Nyeri otot
2 Mual
3 Sakit kepala
4 Lainnya

Q6a Gejala lainnya ____
____

Q7. Pada keseluruhan periode sakit, kapan Anda merasa itu terburuk?
Tuliskan "hari terburuk" pada tanggal

__/__/____	dd/mm/yyyy
01/01/2097	Tidak tahu
01/01/2098	Menolak menjawab

Q8. Pada saat interview ini, apakah Anda masih merasa sakit?

1	Ya	***Lanjut ke Q11***
0	Tidak	

Q9. Apakah Anda sudah pulih sepenuhnya dari episode sakit ini?

1	Ya	
0	Tidak	***Lanjut ke Q11***
8	Menolak menjawab	***Lanjut ke Q11***

Q10. Kapan Anda merasa pulih sepenuhnya pada episode sakit ini?
Tuliskan "pulih sepenuhnya" pada tanggal di kalender,

__/__/____	dd/mm/yyyy
01/01/2097	Tidak tahu
01/01/2098	Menolak menjawab

Q11. Berapa lama sejak Anda merasa sakit hingga memutuskan pergi untuk mendapat layanan kesehatan? (Pilih salah satu)

1	Dalam 24 jam
2	Antara 24 and 48 jam
3	Setelah 48 jam
7	Tidak tahu
8	Menolak menjawab

Perawatan yang diterima pasien dari hari merasa sakit hingga saat ini

Catatan pewawancara: Jika ada KDRS, Mohon mengisi Q12 dan 13 dari form Rumah Sakit

Q12. Apakah rumah sakit tempat Anda dirawat adalah milik pemerintah, swasta-sosial atau swasta?

1 Milik pemerintah
2 Swasta - sosial
3 Swasta
7 Tidak tahu
8 Menolak menjawab

Q13. Indikasikan tipe rumah sakit tempat Anda yang paling lama menjalani perawatan rawat inap?

1 Universitas, rumah sakit rujukan atau tersier
2 Rumah sakit lain
7 Tidak tahu
8 Menolak menjawab

Rawat Inap

BACAKAN: Pada pertanyaan selanjutnya saya hendak mencari tahu apakah Anda menghabiskan waktu 1 atau lebih hari di rumah sakit.

Q14. Apakah Anda dirawat inap selama episode sakit tersebut?

1	Ya	
0	Tidak	***Lanjut bacakan instruksi sebelum Q26***
8	Menolak menjawab	***Lanjut bacakan instruksi sebelum Q26***

Q14a. Kapan Anda dirawat di rumah sakit?
Tuliskan "masuk" pada tanggal kalender.

__/__/__	dd/mm/yy
01/01/2097	Tidak tahu
01/01/2098	Menolak menjawab

Q14b. Selama masa rawat inap, berapa malam yang Anda habiskan di rumah sakit? __ __ . __ __

98 Menolak menjawab

Q14c. Kapan Anda keluar dari rumah sakit?
Tuliskan "keluar" pada tanggal kalender.

__/__/__	dd/mm/yy
01/01/2097	Tidak tahu
01/01/2098	Menolak menjawab

BACAKAN: Sekarang saya akan menanyakan beberapa pertanyaan terkait durasi raat inap Anda, pengeluaran Anda selama perawatan dan pengeluaran lain terkait rawat inap tersebut.

Q15. Dapatkah Anda memberi tahu saya kelas bangsal rawatan Anda selama di rumah sakit?

1. VIP
2. Kelas 1
3. Kelas 2
4. Kelas 3

Q16. Selama masa rawat inap, apakah Anda sempat dirawat di intensive care unit (ICU)?

1	Ya	
0	Tidak	***Lanjut bacakan instruksi sebelum Q18***
8	Menolak menjawab	***Lanjut bacakan instruksi sebelum Q18***

Q17. Berapa hari Anda dirawat di ICU? (jika pasien dirawat di ICU selama 1 hari atau kurang, tuliskan 1)

__ __ . __ __

98 Menolak menjawab

BACAKAN: Sekarang saya akan menanyakan mengenai pengeluaran mandiri yang Anda bayar ke fasilitas kesehatan. Pengeluaran mandiri adalah jumlah uang yang Anda atau keluarga Anda bayarkan untuk biaya perawatan Anda, baik itu Anda menerima penggantian atau tidak dari jumlah tersebut selama episode sakit Anda.

Q18. Berapa estimasi pengeluaran mandiri Anda untuk perawatan yang Anda terima selama rawat inap? (Dapat dibulatkan). Jika tidak ada pengeluaran, masukkan “0”.
Mohon dimasukkan baik pengeluaran yang telah dibayarkan dan pengeluaran yang akan terjadi

__ __ __ __ __ __ __

7 Tidak tahu ***Lanjut bacakan instruksi sebelum Q23***
8 Menolak menjawab ***Lanjut bacakan instruksi sebelum Q23***

Q19. Dapatkah Anda memilah milah pengeluaran Anda berdasar jenis layanan yang Anda dapatkan selama rawat inap?

1 Ya
0 Tidak ***Lanjut bacakan instruksi sebelum Q23***
7 Tidak tahu ***Lanjut bacakan instruksi sebelum Q23***
8 Menolak menjawab ***Lanjut bacakan instruksi sebelum Q23***

Q20. Berapa pengeluaran mandiri rumah tangga Anda yang dibayarkan untuk konsultasi, saat mulai hingga selesai rawat inap?
Jika tidak ada pengeluaran, masukkan "0". __ __ __ __ __ __ __ __

7 Tidak tahu
8 Menolak menjawab
9 Tidak dapat diaplikasikan

Q21. Berapa pengeluaran mandiri rumah tangga Anda yang dibayarkan untuk uji diagnostic seperti cek darah, tes dengue, rontgent atau sonogram?
Jika tidak ada pengeluaran, masukkan "0". __ __ __ __ __ __ __ __

7 Tidak tahu
8 Menolak menjawab
9 Tidak dapat diaplikasikan

Q22. Berapa pengeluaran mandiri rumah tangga Anda yang dibayarkan untuk pengobatan, cairan infus dan peralatan medis atau bank darah?
Jika tidak ada pengeluaran, masukkan "0". __ __ __ __ __ __ __ __

7 Tidak tahu
8 Menolak menjawab
9 Tidak dapat diaplikasikan

BACAKAN: Sekarang saya akan menanyakan mengenai pengeluaran Anda terkait episode penyakit dari awal hingga selesai merasa sakit [Merespons Q1], seperti untuk transportasi, makan, atau penginapan. Termasuk pengeluaran lain oleh anggota keluarga yang menemani masa rawat inap Anda, anggota keluarga lain dan kerabat.

Q23a. Apa moda transportasi yang Anda gunakan untuk menuju ke rumah sakit? Berapa jaraknya____ _________________________

Catatan pewawancara: jika menggunakan mobil atau motor pribadi tanyakan jaraknya, dan jika pasien tidak tahu maka gunakan google map

Q23b. Berapa estimasi pengeluaran mandiri keluarga Anda untuk transportasi menuju rumah sakit, termasuk ambulans? Jika tidak ada pengeluaran, masukkan "0".

Jika keluarga memiliki mobil, estimasikan jarak tempuh perjalanan (datang dan pergi) dan gunakan proxy Rp. 16.000 per km. Dan jika pasien menggunakan motor gunakan proxy Rp 14.000 per km. Jika tidak ada pengeluaran, catat "0.00".

__ __ __ __ __ __ __ __ __

7 Tidak tahu
8 Menolak menjawab
9 Tidak dapat diaplikasikan

Q23c. Apa moda transportasi yang Anda gunakan untuk pulang dari rumah sakit? Berapa jaraknya____ _________________________

Catatan pewawancara: jika menggunakan mobil atau motor pribadi tanyakan jaraknya, dan jika pasien tidak tahu maka gunakan google map

Q23d. Berapa estimasi pengeluaran mandiri keluarga Anda untuk transportasi dari rumah sakit, termasuk ambulans? Jika tidak ada pengeluaran, masukkan "0".

Jika keluarga memiliki mobil, estimasikan jarak tempuh perjalanan (datang dan pergi) dan gunakan proxy Rp. 16.000 per km. Dan jika pasien menggunakan motor gunakan proxy Rp 14.000 per km. Jika tidak ada pengeluaran, catat "0.00".

__ __ __ __ __ __ __ __ __

7 Tidak tahu
8 Menolak menjawab
9 Tidak dapat diaplikasikan

Q24. Berapa estimasi pengeluaran mandiri keluarga Anda untuk makan? Jika tidak ada pengeluaran, masukkan "0". __ __ __ __ __ __ __ __

7 Tidak tahu
8 Menolak menjawab
9 Tidak dapat diaplikasikan

Q25. Berapa estimasi pengeluaran mandiri keluarga Anda untuk penginapan di fasilitas non-kesehatan seperti hotel? Jika tidak ada pengeluaran, masukkan "0". __ __ __ __ __ __ __ __

7 Tidak tahu
8 Menolak menjawab
9 Tidak dapat diaplikasikan

Rawat Jalan

BACAKAN: Pertanyaan berikut ini akan terkait layanan rawat jalan sejak Anda mulai merasa hingga berakhirnya sakit [Merespons Q1]. Mohon **termasuk** perawatan dari berbagai jenis fasilitas kesehatan seperti apotek, pengobatan tradisional, klinik swasta, puskesmas, bidan, poli rawat jalan dan/atau unit gawat darurat, dan lab swasta

Q26. Apakah Anda mengunjungi fasilitas rawat jalan pada episode dengue Anda yang terakhir? (Tunjukkan form peraga 1).

1	Ya	
0	Tidak	***Lanjutkan ke Q40***
8	Menolak menjawab	***Lanjutkan ke Q40***

BACAKAN: Pikirkan tentang waktu yang Anda habiskan untuk akses fasilitas kesehatan berikut selama episode sakit dimulai **[tanggal pada Q1]**. Kemudian spesifikkan jenis perawatan yang Anda terima pada tiap fasilitas kesehatan yang Anda kunjungi (konsultasi, uji diagnostic, dan/atau pengobatan) dan cobalah mengingat kembali jumlah pengeluaran mandiri keluarga Anda, *anggota keluarga lain dan kerabat* pada tiap jenis layanan tersebut.

Q27. Berapa kali kunjungan pada fasilitas kesehatan berikut ini selama periode sakit Anda? ***Tunjukkan form 1 (kalender) dan 2 (daftar fasilitas kesehatan)***

Visit	Number of visits
Apotek (01)	
Pengobatan tradisional (02)	
Dokter praktek pribadi (03)	
Puskesmas (04)	
Bidan (05)	
Poli rawat jalan rumah sakit (06)	
Unit Gawat Darurat (UGD) (07)	
Lab swasta (08)	
Puskesmas pembantu (09)	
Lainnya (10)	
Sebutkan lainnya_______________	

Q28. Pilihlah dari berikut ini tipe fasilitas kesehatan yang Anda kunjungi selama periode sakit. Apakah Anda dapat memberi tanggal pada tiap kunjungan? (*Tunjukkan form 2 dan form 1, pilih salah satu pada tiap kunjungan)*

Kunjungan	Pertama	Kedua	Ketiga	Keempat	Kelima	Keenam
Tanggal						
Type of facility						
Apotek (01)						
Pengobatan tradisional (02)						
Dokter praktek pribadi (03)						
Puskesmas (04)						
Bidan (05)						
Poli rawat jalan rumah sakit (06)						
UGD (07)						
Lab swasta (08)						
Puskesmas pembantu (09)						
Lainnya (10),<br>sebutkan__________						
Tidak tahu (97)						
Menolak menjawab (98)						

BACAKAN: Sebutkan nama fasilitas kesehatan manakah yang Anda kunjungi? (Isikan kolom di samping tipe fasilitas kesehatan yang dipilih pasien)

Catatan untuk pewawancara: Fasilitas kesehatan "public" adalah fasilitas kesehatan yang dioperasikan oleh pemerintah dalam berbagai tingkatan, dan "swasta-sosial" adalah fasilitas kesehatan yang dioperasikan oleh organisasi sosial non-profit, "swasta" adalah dokter praktek pribadi atau klinik bersama.

Q29. Apa tipe fasilitas kesehatan yang Anda kunjungi, selama kunjungan kali ke-X Anda? Apakah fasilitas kesehatan public atau non-publik? (Pilih salah satu per kunjungan)

Kunjungan	Publik (1)	Swasta-sosial (2)	Swasta (3)	Tidak tahu (7)	Menolak menjawab (8)
Pertama					
Kedua					
Ketiga					
Keempat					
Kelima					
Keenam					

Q30. Jenis layanan apa yang Anda terima selama kunjungan kali ke-X Anda? (Cek yang diaplikasikan)

Kunjungan	Konsultasi	Uji diagnostic seperti rontgent, sampel darah	Pengobatan medis, infus	Rujukan	Tidak tahu (7)	Menolak menjawab (8)
Pertama						
Kedua						
Ketiga						
Keempat						
Kelima						
Keenam						

BACAKAN: Sekarang saya akan menanyakan mengenai pengeluaran mandiri keluarga Anda, *anggota keluarga lain dan kerabat* terhadap fasilitas berikut ini.
Catatan pewawancara: Bila memungkinkan, kroscek dengan jumlah tagihan yang tersedia dari fasilitas kesehatan.

Q31. Mohon sebutkan perkiraan terbaik Anda dari total pengeluaran mandiri terhadap layanan yang Anda terima dari fasilitas kesehatan? Mohon termasuk pengeluaran yang telah dilakukan dan tagihan yang akan datang.
Jika tidak ada pengluaran, mohon masukkan "0,00"

Kunjungan	Jumlah, IDR	***Jika 0 lompat ke instruksi sebelum Q36a***	Tidak tahu (7)	***Lompat ke instruksi sebelum Q36a***	Menolak menjawab (8)	***Lompat ke instruksi sebelum Q36a***
Pertama						
Kedua						
Ketiga						
Keempat						
Kelima						
Keenam						

Q32. Apakah Anda dapat mengurai jumlah pembayaran berdasar tipe layanan yang diterima?

Kunjungan	Ya (1)	Tidak (0)	***Lompat ke instruksi sebelum Q36a***	Menolak menjawab (8)	***Lompat ke instruksi sebelum Q36a***	Tidak dapat diterapkan (9)	***Lompat ke instruksi sebelum Q36a***
Pertama							
Kedua							
Ketiga							
Keempat							
Kelima							
Keenam							

Q33. Pengeluaran mandiri pada: konsultasi. (*Jika tidak ada pembayaran tuliskan "0,00"*)

Visit	Jumlah, IDR	Tidak tahu (7)	Menolak menjawab (8)	Tidak dapat diterapkan (9)
Pertama				
Kedua				
Ketiga				
Keempat				
Kelima				
Keenam				

Q34. Pengeluaran mandiri pada: transfuse darah atau uji diagnostic seperti rontgent, sampel darah, sonogram… (*Jika tidak ada pembayaran tuliskan "0,00"*)

Kunjungan	Jumlah, IDR	Tidak tahu (7)	Menolak menjawab (8)	Tidak dapat diterapkan (9)
Pertama				
Kedua				
Ketiga				
Keempat				
Kelima				
Keenam				

Q35. Pengeluaran mandiri pada: pengobatan, set peralatan medis. (*Jika tidak ada pembayaran tuliskan "0,00"*)

Kunjungan	Jumlah, IDR	Tidak tahu (7)	Menolak menjawab (8)	Tidak dapat diterapkan (9)
Pertama				
Kedua				
Ketiga				
Keempat				
Kelima				
Keenam				

BACAKAN: Sekarang saya akan menanyakan terkait pengeluaran keluarga Anda spesifik untuk fasilitas kesehatan selama periode sakit seperti untuk transportasi, makan/minum, dan penginapan. Termasuk pengeluaran anggota keluarga Anda, *anggota keluarga lain dan kerabat* yang menemani selama kunjungan pertama (kedua, ketiga,…).

Q36a. Apakah moda transportasi yang Anda gunakan untuk menuju fasilitas kesehatan?

Catatan pewawancara: jika menggunakan mobil atau motor pribadi, tanyakan jaraknya ke fasilitas kesehatan tersebut

Kunjungan	Moda Transportasi	Jarak (km)
Pertama		
Kedua		
Ketiga		
Keempat		
Kelima		
Keenam		

Q36b. Mohon sebutkan perkiraan terbaik Anda terkait pengeluaran mandiri untuk transportasi menuju fasilitas kesehatan)
Jika keluarga memiliki kendaraan, perkirakan jarak tempuh perjalanan (pulang-pergi) dan gunakan IDR 16,000 per Km sebagai acuan dengan mobil dan IDR 14,000 per Km bila dengan motor. Jika tidak ada pengeluaran tuliskan "0,00".

Kunjungan	Jumlah, IDR	Tidak tahu (7)	Menolak menjawab (8)	Tidak dapat diterapkan (9)
Pertama				
Kedua				
Ketiga				
Keempat				
Kelima				
Keenam				

Q36c. Apakah moda transportasi yang Anda gunakan dari fasilitas kesehatan?

Catatan pewawancara: jika menggunakan mobil atau motor pribadi, tanyakan jaraknya ke fasilitas kesehatan tersebut

Kunjungan	Moda Transportasi	Jarak (km)
Pertama		
Kedua		
Ketiga		
Keempat		
Kelima		
Keenam		

Q36d. Mohon sebutkan perkiraan terbaik Anda terkait pengeluaran mandiri untuk transportasi dari fasilitas kesehatan)
Jika keluarga memiliki kendaraan, perkirakan jarak tempuh perjalanan (pulang-pergi) dan gunakan IDR 16,000 per Km sebagai acuan dengan mobil dan IDR 14,000 per Km bila dengan motor. Jika tidak ada pengeluaran tuliskan "0,00".

Kunjungan	Jumlah, IDR	Tidak tahu (7)	Menolak menjawab (8)	Tidak dapat diterapkan (9)
Pertama				
Kedua				
Ketiga				
Keempat				
Kelima				
Keenam				

Q37. Mohon sebutkan perkiraan terbaik Anda terkait pengeluaran mandiri untuk makan/minum.
Jika tidak ada pengeluaran tuliskan "0,00".

Kunjungan	Jumlah, IDR	Tidak tahu (7)	Menolak menjawab (8)	Tidak dapat diterapkan (9)
Pertama				
Kedua				
Ketiga				
Keempat				
Kelima				
Keenam				

Q38. Mohon sebutkan perkiraan terbaik Anda terkait pengeluaran mandiri untuk penginapan seperti hotel.
Jika tidak ada pengeluaran tuliskan "0,00".

Kunjungan	Jumlah, IDR	Tidak tahu (7)	Menolak menjawab (8)	Tidak dapat diterapkan (9)
Pertama				
Kedua				
Ketiga				
Keempat				
Kelima				
Keenam				

Q39. Apakah ada fasilitas kesehatan lain yang Anda kunjungi selama periode sakit dimulai dari saat Anda mulai merasa sakit? [Respons terhadap Q1]?

1 Ya ***Ulangi proses dari Q28 hingga Q39***
0 Tidak

<u>Dampak penyakit pada anggota keluarga,</u> anggota keluarga lain dan kerabat <u>selama periode sakit sejak awal hingga selesai [Respons terhadap Q1]</u>

BACAKAN: Saya akan mempelajari bagaimana sakit DBD Anda terakhir yang dimulai sejak [tanggal] yang mempengaruhi Anda dan keluarga Anda, anggota keluarga lain dan kerabat.

BACAKAN: Mari kita bicara tentang Anda terlebih dahulu.

<u>Pasien</u>

Q40. *Mohon tuliskan jenis kelamin pasien tanpa menanyakan kepadanya.*
1 Laki-laki
2 Perempuan

Q41. Berapa usia Anda saat ini? _____
999 Tidak tahu
998 Menolak menjawab

Q41a. Kapan tanggal lahir Anda (hari/bulan/tahun)?
__/__/____ dd/mm/yyyy
01/01/2097 Tidak tahu
01/01/2098 Menolak menjawab

Q42. Apa tingkat pendidikan terakhir yang Anda selesaikan? (Pilih salah satu)
Tunjukkan Form 3

01	Tidak dapat baca-tulis
02	Mampu baca-tulis namun tidak sekolah
03	Sekolah tapi hanya satu tahun
04	Taman kanak - kanak
05	Sekolah Dasar
06	Sekolah Menengah Pertama
07	Sekolah Menengah Atas
08	Diploma
09	Universitas, Sarjana
10	Universitas, Pasca Sarjana (Magister, Doktoral)
11	Ijazah profesi
97	Tidak tahu
98	Menolak menjawab
99	Tidak dapat diaplikasikan

Q43. Apakah Anda saat ini sedang menempuh pendidikan?

1	Ya	
0	Tidak	***Lanjut ke Q46***
8	Menolak menjawab	***Lanjut ke Q46***

Q44. Apakah Anda kehilangan waktu sekolah Anda selama sakit?

1	Ya	
0	Tidak	***Lanjut ke Q46***
8	Menolak menjawab	

Q45. Berapa hari sekolah Anda yang hilang karena sakit?
Tuliskan “0” jika tidak ada

_____

97	Tidak tahu
98	Menolak menjawab
99	Tidak dapat diterapkan

Q46. Apakah Anda bekerja dan dibayar?

1	Ya	
0	Tidak	***Lanjut ke Q51a***
8	Menolak menjawab	

Q47. Apakah Anda kehilangan hari kerja selama sakit?
Jika pasien tidak kehilangan hari kerja, mohon konfirmasi apakah kehilangan pendapatan.

1	Ya	
0	Tidak	***Lanjut ke Q51a***
7	Tidak tahu	***Lanjut ke Q51a***
8	Menolak menjawab	***Lanjut ke Q51a***

Q48. Berapa hari kerja Anda yang hilang selama sakit?
Tuliskan “0” jika tidak ada

_____

97	Tidak tahu
98	Menolak menjawab
99	Tidak dapat diterapkan

Q49. Apakah Anda menerima kompensasi (tidak potong gaji) selama Anda sakit, apakah itu tempat kerja Anda atau pihak lain yang menanggung selama Anda sakit?

1	Ya	
0	Tidak	***Lanjut ke Q51a***
7	Tidak tahu	***Lanjut ke Q51a***
8	Menolak menjawab	***Lanjut ke Q51a***
9	Tidak dapat diterapkan	***Lanjut ke Q51a***

Q50. Berapa hari gaji yang Anda terima selama sakit?
Tuliskan "0" jika tidak ada _____

97	Tidak tahu
98	Menolak menjawab
99	Tidak dapat diterapkan

Q51a. Apakah Anda kehilangan pendapatan selama sakit?

1	Ya	
0	Tidak	***lanjut ke Q52***
7	Tidak tahu	***lanjut ke Q52***
8	Menolak menjawab	***lanjut ke Q52***

Q51b. Berapa pengeluaran Anda yang hilang? (Berikan perkiraan terbaik Anda)

____

7	Tidak tahu
8	Menolak menjawab
9	Tidak dapat diterapkan

Q52. Apakah Anda menerima bantuan keuangan atau non-keuangan dari keluarga atau komunitas selama Anda sakit?

1	Ya	
0	Tidak	***Lanjut ke Q55***
8	Menolak menjawab	***Lanjut ke Q55***

Q53. Berapa perkiraan terbaik Anda nilai bantuan non-keuangan yang Anda dapatkan?
Benar 0 jika tidak ada bantuan non-keuangan.
Tuliskan "0" jika tidak ada _____

97	Tidak tahu
98	Menolak menjawab
99	Tidak dapat diterapkan

Catatan pewawancara: Jika tidak tahu, tuliskan jenis bantuan non-keuangan yang diterima pasien dan keluarga ____

____

Q54. Berapakah total bantuan keuangan yang Anda dapatkan dalam IDR?
Tuliskan "0" jika tidak ada _____

97	Tidak tahu
98	Menolak menjawab
99	Tidak dapat diterapkan

Jumlah Anggota Keluarga

Q55. Berapa jumlah anggota keluarga selain Anda?

_____

00	Nol	***Akhiri wawancara***
98	Menolak menjawab	

.

Q56. Berapa anggota keluarga yang terdampak sakit Anda secara langsung selama periode sakit Anda?
BACAKAN: Anggota keluarga adalah mereka yang tinggal bersama Anda dan berbagi makanan. Anggota keluarga terdampak jika dia menghabiskan uang dan waktu lebih dari jumlah biasanya karena merawat Anda atau kehilangan waktu kerja, pendapatan, sekolah karena sakit Anda.

_____

00	Nol	***Akhiri wawancara***
98	Menolak menjawab	***Akhiri wawancara***

BACAKAN: Sekarang saya akan menanyakan Anda terkait anggota keluarga Anda yang terdampak penyakit Anda. [Respons terhadap Q56].

Mereka yang merawat

Q57. Apa hubungan Anda dengan anggota keluarga (pertama, kedua,…) Anda? (pilih salah satu)
Tunjukkan form 4

Hubungan	Anggota Keluarga							
	Anggota pertama	Anggota kedua	Anggota ketiga	Anggota keempat	Anggota kelima	Anggota keenam	Anggota ketujuh	Anggota kedelapan
Ibu (01)								
Ayah (02)								
Suami (03)								
Istri (04)								
Teman serumah (05)								
Saudara perempuan (06)								
Saudara laki-laki (07)								
Anak laki-laki (08)								
Anak perempuan (09)								
Nenek (10)								
Kakek (11)								
Paman (12)								
Bibi (13)								
Pembantu (14)								
Pengasuh (15)								
Lainnya (16) sebutkan__________								
Menolak menjawab (98)								

Q58. Berapa usia anggota keluarga Anda?

Anggota keluarga	Usia dalam tahun	Tidak tahu (997)	Menolak menjawab (998)
Anggota pertama			
Anggota kedua			
Anggota ketiga			
Anggota keempat			
Anggota kelima			
Anggota keenam			
Anggota ketujuh			
Anggota kedelapan			

Q59. Apa tingkat pendidikan terakhir anggota keluarga Anda? (pilih salah satu)
Tunjukkan form 3

Hubungan	Anggota Keluarga							
	Anggota pertama	Anggota kedua	Anggota ketiga	Anggota keempat	Anggota kelima	Anggota keenam	Anggota ketujuh	Anggota kedelapan
Tidak dapat baca-tulis (01)								
Mampu baca-tulis namun tidak sekolah (02)								
Sekolah tapi hanya satu tahun (03)								
Taman kanak - kanak (04)								
Sekolah Dasar (05)								
Sekolah Menengah Pertama (06)								
Sekolah Menengah Atas (07)								
Diploma (08)								
Universitas, Sarjana (09)								
Universitas, Pasca Sarjana (Magister, Doktoral) (10)								
Ijazah profesi (11)								
Tidak tahu (97)								
Menolak menjawab (98)								
Tidak dapat diaplikasikan (99)								

Q60. Apakah anggota keluarga Anda saat ini sedang menempuh pendidikan?

Anggota keluarga	Ya (kode:1)	Tidak (Kode:2)	***Lanjut ke Q62***	Tidak tahu (Kode:7)	***Lanjut ke Q62***	Menolak menjawab (Kode:8)	***Lanjut ke Q62***
Anggota pertama							
Anggota kedua							
Anggota ketiga							
Anggota keempat							
Anggota kelima							
Anggota keenam							
Anggota ketujuh							
Anggota kedelapan							

Q61. Apakah anggota keluarga Anda kehilangan hari sekolah karena penyakit Anda?

Anggota keluarga	Ya (kode:1)	Tidak (Kode:2)	***Lanjut ke Q62***	Tidak tahu (Kode:7)	***Lanjut ke Q62***	Menolak menjawab (Kode:8)	***Lanjut ke Q62***
Anggota pertama							
Anggota kedua							
Anggota ketiga							
Anggota keempat							
Anggota kelima							
Anggota keenam							
Anggota ketujuh							
Anggota kedelapan							

Q62. Berapa hari sekolah anggota keluarga yang hilang karena penyakit Anda?
Tuliskan "0" jika tidak ada

Anggota keluarga	Jumlah hari yang hilang	Tidak tahu (97)	Menolak menjawab (98)
Anggota pertama			
Anggota kedua			
Anggota ketiga			
Anggota keempat			
Anggota kelima			
Anggota keenam			
Anggota ketujuh			
Anggota kedelapan			

Q63. Apakah anggota keluarga Anda bekerja dan dibayar?

Anggota keluarga	Ya (kode:1)	Tidak (Kode:2)	***Lanjut ke Q67a***	Tidak tahu (Kode:7)	***Lanjut ke Q67a***	Menolak menjawab (Kode:8)	***Lanjut ke Q67a***
Anggota pertama							
Anggota kedua							
Anggota ketiga							
Anggota keempat							
Anggota kelima							
Anggota keenam							
Anggota ketujuh							
Anggota kedelapan							

Q64. Apakah anggota keluarga Anda kehilangan hari kerja karena penyakit Anda?

Anggota keluarga	Ya (kode:1)	Tidak (Kode:2)	***Lanjut ke Q67a***	Tidak tahu (Kode:7)	***Lanjut ke Q67a***	Menolak menjawab (Kode:8)	***Lanjut ke Q67***
Anggota pertama							
Anggota kedua							
Anggota ketiga							
Anggota keempat							
Anggota kelima							
Anggota keenam							
Anggota ketujuh							
Anggota kedelapan							

Q65. Berapa hari kerja anggota keluarga yang hilang karena penyakit Anda?
Tuliskan "0" jika tidak ada

Anggota keluarga	Jumlah hari	Tidak tahu (Kode:97)	***Lanjut ke Q68***	Menolak menjawab (Kode:98)	***Lanjut ke Q68***	Tidak dapat diterapkan (Kode:99)	***Lanjut ke Q68***
Anggota pertama							
Anggota kedua							
Anggota ketiga							
Anggota keempat							
Anggota kelima							
Anggota keenam							
Anggota ketujuh							

Anggota kedelapan							

Q66. Apakah anggota keluarga menerima kompensasi atas absennya mereka dari pekerjaan ketika merawat Anda sakit? (dari tempat kerja atau pihak yang menanggung)

Anggota keluarga	Ya (Kode: 1)	Tidak (Kode: 0)	***Lanjut ke Q67a***	Tidak tahu (Kode:7)	***Lanjut ke Q67a***	Menolak menjawab (Kode:8)	**Lanjut ke Q67a**	Tidak dapat diterapkan (Kode:9)	***Lanjut ke Q67a***
Anggota pertama									
Anggota kedua									
Anggota ketiga									
Anggota keempat									
Anggota kelima									
Anggota keenam									
Anggota ketujuh									
Anggota kedelapan									

Q67. Berapa hari kerja yang didapat ke anggota keluarga Anda?

Anggota keluarga	Jumlah hari yang dibayarkan	Tidak tahu (97)	Menolak menjawab (98)
Anggota pertama			
Anggota kedua			
Anggota ketiga			
Anggota keempat			
Anggota kelima			
Anggota keenam			
Anggota ketujuh			
Anggota kedelapan			

Q67a. Apakah anggota keluarga Anda kehilangan pendapatan karena penyakit Anda?

Anggota keluarga	Ya (kode:1)	Tidak (Kode:2)	***Lanjut ke Q68***	Tidak tahu (Kode:7)	***Lanjut ke Q68***	Menolak menjawab (Kode:8)	***Lanjut ke Q68***
Anggota pertama							
Anggota kedua							
Anggota ketiga							
Anggota keempat							
Anggota kelima							
Anggota keenam							
Anggota ketujuh							
Anggota kedelapan							

Q67b. Berapa pendapatan anggota keluarga Anda yang hilang? (Mohon berikan perkiraan terbaik).
Jika tidak ada tuliskan "0,00".

Anggota keluarga	Jumlah, IDR	Tidak tahu (7)	Menolak menjawab (8)	Tidak dapat diterapkan (9)
Anggota pertama				
Anggota kedua				
Anggota ketiga				

Anggota keempat				
Anggota kelima				
Anggota keenam				
Anggota ketujuh				
Anggota kedelapan				

Q68. Dari jumlah hari sakit selama episode sakit Anda yang dimulai sejak sakit hingga saat pemulihan (tanggal wawancara), berapa jumlah hari tambahan yang dihabiskan anggota keluarga untuk perawatan lanjutan Anda?
Jika tidak ada, masukkan 0

Anggota keluarga	Jumlah hari	***Jika “Nol” Lanjut ke Q70***	Tidak tahu (Kode:97)	***Lanjut ke Q70***	Menolak menjawab (Kode:98)	**Lanjut ke Q70**	Tidak dapat diterapkan (Kode:99)	***Lanjut ke Q70***
Anggota pertama								
Anggota kedua								
Anggota ketiga								
Anggota keempat								
Anggota kelima								
Anggota keenam								
Anggota ketujuh								
Anggota kedelapan								

BACAKAN: Dari pertanyaan berikut, pikirkan tentang jumlah tambahan jam per hari yang Anda dapatkan dari anggota keluarga. Tambahan jam merujuk pada jumlah jam diluar biasanya anggota keluarga menghabiskan waktu bersama Anda. Dalam memperkirakan waktu, mohon masukkan waktu di rumah, pada fasilitas kesehatan dan selama perjalanan menuju dan dari fasilitas kesehatan.

Q69. Selama periode ini, berapa rata – rata jumlah tambahan jam anggota keluarga untuk merawat Anda? [Respons terhadap Q68]
Jika tidak ada, masukkan 0

Anggota keluarga	Rata-rata tambahan jam untuk perawatan harian	Tidak tahu (Kode:97)	Menolak menjawab (Kode:98)	Tidak dapat diterapkan (Kode:99)
Anggota pertama				
Anggota kedua				
Anggota ketiga				
Anggota keempat				
Anggota kelima				
Anggota keenam				
Anggota ketujuh				
Anggota kedelapan				

Q70. Apakah ada pengeluaran transportasi, makan/minum dan penginapan tambahan dari yang telah disebutkan sebelumnya untuk mengunjungi fasilitas kesehatan?

Anggota keluarga	Ya (Kode:1)	No (Kode: 0)	***Lanjut ke anggota keluarga lainnya***	Tidak tahu (Kode:7)	***Lanjut ke anggota keluarga lainnya***	Menolak menjawab (Kode:8)	***Lanjut ke anggota keluarga lainnya***	Tidak dapat diterapkan (Kode:9)	***Lanjut ke anggota keluarga lainnya***
Anggota pertama									
Anggota kedua									
Anggota ketiga									
Anggota keempat									
Anggota kelima									
Anggota keenam									
Anggota ketujuh									
Anggota kedelapan									

Q71. Untuk pertanyaan berikut ini, berikan perkiraan terbaik terhadap jumlah total tambahan pengeluaran dari tambahan hari untuk merawat Anda yang belum termasuk yang disebutkan pada pertanyaan sebelumnya? [Jumlah hari yang dilaporkan pada Q68]
Jika tidak ada pengeluaran, tuliskan "0,00".

Anggota keluarga	Amount, IDR	Tidak tahu (7)	Menolak menjawab (8)	Tidak dapat diterapkan (9)
Anggota pertama				
Anggota kedua				
Anggota ketiga				
Anggota keempat				
Anggota kelima				
Anggota keenam				
Anggota ketujuh				
Anggota kedelapan				

Cek Q56, jika jawabannya lebih dari 1, maka ulangi Q57-71 untuk semua anggota keluarga terdaftar. Jika Q56 sama dengan 1, maka bacakan kalimat berikut untuk mengakhiri wawancara.

BACAKAN: Terima kasih atas partisipasi Anda! Jika ada pertanyaan lebih lanjut terkait wawancara atau hasil penelitian ini, Anda dapat menghubungi saya, dr. Nandyan N. Wilastonegoro, MScIH di nomor 0813-2884-3888 atau 0274-5306089 di Fakultas Kedokteran, Kesehatan Masyarakat dan Keperawatan, Universitas Gadjah Mada (FKKMK UGM).

Supplementary Information

Cost of dengue illness in Indonesia across hospital, ambulatory and non-medical settings

S3: Questionnaire S3. Pediatric questionnaire (patients under age 18) in English

Child confirmed Dengue Patient Questionnaire (Age ≤ 17)

Name of child patient ______________
Patient study ID ______________
Name of the interviewee (proxy) ______________
Date of interview ______________

Interviewer

Q0. *The name of the site the patient was recruited from. (Choose one)*

1 Hospital_____________________
2 Health Center________________
3 Outside professional health system through (name of community health worker) _______________________

Q00. *Interviewee*

1 Patient him/herself
2 Patient's proxy

READ: Now we are going to talk about [Child's name] illness period. I will give you a calendar that will help you answer the following questions.
Show calendar flashcard (form 1) to the proxy for the following questions and continue to use through Q39. Write "today" in the box for today's date.

Q1. When did [Child's name] start feeling ill?
Write "Illness started" on that date in the calendar.

__/__/____ dd/mm/yyyy
01/01/2097 Don't Know
01/01/2098 Refuse to Answer

Q2. Did [Child name] have fever during this illness episode that started in [date in Q1]?

1 Yes
0 No ***Skip to Q6***
8 Refuse to Answer ***Skip to Q6***

Q.3 When did [Child's name]'s fever start?
Write "fever started" on that date in the calendar,

__/__/____ dd/mm/yyyy
01/01/2097 Don't Know
01/01/2098 Refuse to Answer

Q.4 Did [Child's name]'s fever end?

1 Yes
0 No ***Skip to Q6***
8 Refuse to Answer ***Skip to Q6***

Q.5 When did [Child's name]'s fever end?
Write "fever ended" on that date in the calendar,

__/__/____ dd/mm/yyyy
01/01/2097 Don't Know
01/01/2098 Refuse to Answer

Q6. What other symptoms did [Child name] have?

1 Myalgia
2 Nausea
3 Headache

4 Others

Q6a Other Symptoms ____
____
____

Q7. Over [Child's name]'s entire illness, on which day did [Child's name] feel worst?
Write "worst day" on that date in the calendar,

__/__/____	dd/mm/yyyy
01/01/2097	Don't Know
01/01/2098	Refuse to Answer

Q8. At the time of this interview, is [Child's name] still feeling ill?

1	Yes	***Skip to Q11***
0	No	

Q9. Have [Child's name] recovered completely from this illness period?

1	Yes	
0	No	***Skip to Q11***
8	Refused to Answer	***Skip to Q11***

Q10. When did [Child's name] fully recover from the illness episode?
Write "recovered completely" on that date in the calendar,

__/__/____	dd/mm/yyyy
01/01/2097	Don't Know
01/01/2098	Refuse to Answer

Q11. How soon after [Child's name] started to feel ill did he/she first receive care by a medical care provider? (Choose one)

1	Within 24 hours
2	Between 24 and 48 hours
3	After 48 hours
7	Don't Know
8	Refused to Answer

Care received by child from the day he/she started feeling ill until today

NOTE to interviewer: if you have forms from hospital please fill Q12 and 13 from the hospital form

Q12. Is the hospital where the patient was admitted a public, social-private, or private?

1 Public
2 Social-private
3 Private
7 Don't Know
8 Refuse to Answer

Q13. Indicate the type of hospital in which you spent the most number of nights?

1 University, tertiary or referral hospital
2 Other hospital
7 Don't Know
8 Refuse to Answer

Hospitalization

Q14. Were [child name] hospitalized during this interview period?

1 Yes
0 No ***Skip to instruction before Q26***
8 Refused to answer ***Skip to instruction before Q26***

Q14a. When were [child's name] admitted to the hospital

Write "admission" on that date in the calendar,

__/__/____ dd/mm/yyyy
01/01/2097 Don't Know
01/01/2098 Refuse to Answer

Q14b. During this hospitalization, for how many nights did [child name] stay in the hospital?

__ __ . __ __
98 Refuse to Answer

Q14c. When were [child's name] discharged the hospital

Write "discharged" on that date in the calendar,

__/__/____ dd/mm/yyyy
01/01/2097 Don't Know
01/01/2098 Refuse to Answer

READ: Now I would like to ask you some questions on the duration of [child name's] hospitalization, the out-of-pocket payments you made for treatment and other spending related to this hospitalization.

Q15. Can you please tell me which type of hospital ward/class did [child's name] stay at for the majority of his/her hospital stay?

1 VIP
2 Class 1
3 Class 2
4 Class 3

Q16. During [child name] hospitalization, did [child name] stay any night in an intensive care unit?

1	Yes	
0	No	***Skip to instruction before Q18***
8	Refuse to Answer	***Skip to instruction before Q18***

Q17. For how many nights did [child name] stay in the intensive care unit? (if patient stayed in the intensive care unit for one night or less then mark "1".

__ __ . __ __

98 Refuse to Answer

READ: Now I will ask about the out-of-pocket payments [child name]'s family made in this facility.
The household out-of-pocket payments are the total amounts paid by you or your family for your treatment, whether or not you received reimbursement for all or part of this paid amount during this interview period.

Q18. Please report your best estimate of the total out-of-pocket payment that your household have paid up to today for services delivered in during [child name] hospitalization? (Round all items to the nearest IDR). If there were no payments, please enter "0".
Please include both payments already made and those that are expected to be made for the services received.

__ __ __ __ __ __ __

7	Don't Know	***Skip to instruction before Q23***
8	Refuse to Answer	***Skip to instruction before Q23***

Q19. Can you break down these payments by type of service received during [child name] stay in the hospital?

1	Yes	
0	No	***Skip to instruction before Q23***
7	Don't Know	***Skip to instruction before Q23***
8	Refuse to Answer	***Skip to instruction before Q23***

Q20. How much did your household pay out-of-pocket for consultation, admission and discharge fees?
If no spending put "0". __ __ __ __ __ __ __ __

7	Don't Know
8	Refuse to Answer
9	Not Applicable

Q21. How much did your household pay out-of-pocket for diagnostic tests such as blood tests, dengue test, x-rays or sonogram?
If no spending put "0". __ __ __ __ __ __ __ __

7	Don't Know
8	Refuse to Answer
9	Not Applicable

Q22. How much did your household pay out-of-pocket on all treatment such as medications, intravenous fluid (IVF), medical equipment (I/V sets) or blood bank.
If no spending put "0" __ __ __ __ __ __ __ __

7	Don't Know
8	Refuse to Answer
9	Not Applicable

READ: Now I would like to ask you questions about other spending related to your illness during this interview period from the day you started feeling ill [Response to Q1] until today, such as spending on transportation, meals or lodging. Include also any expenses by any other household member (person you live with), other family members or friend who accompanied you to the hospital for care.

Q23a. Can you please tell me the type of transportation [child's name] used to go to the hospital?

____

Note to interviewer: if transportation used was a car, ask about the distance from the patient's house to the hospital. If the patient doesn't know the answer then use google map to determine the distance.

Q23b. Please report your best estimate of your household out of pocket spending on transportation to the hospital, including ambulance.

If no spending put "0".

If family or owned vehicle, estimate mileage traveled (two ways travel) and use IDR 16,000 per Km as a proxy for the cost of driving. If family or patient owned Auto-rickshaw/own scooter use IDR 14,000 per Km. If no spending record "0.00".

— — — — — — — — — —

7	Don't Know
8	Refuse to Answer
9	Not Applicable

Q23c. Can you please tell me the type of transportation [child's name] used to get back from the hospital?

____

Note to interviewer: if transportation used was a car, ask about the distance from hospital to patient's house.

Q23d. Please report your best estimate of your household out of pocket spending on transportation from the hospital, including ambulance.

If no spending put "0".

If family or owned vehicle, estimate mileage traveled (two ways travel) and use IDR 16,000 per Km as a proxy for the cost of driving. If family or patient owned Auto-rickshaw/own scooter use IDR 14,000 per Km. If no spending record "0.00".

__ __ __ __ __ __ __ __ __

7 Don't Know

8 Refuse to Answer

9 Not Applicable

Q24. Please report best estimate of your household out-of-pocket spending on food or meals.

If no spending put "0" __ __ __ __ __ __ __ __ __

7 Don't Know

8 Refuse to Answer

9 Not Applicable

Q25. Please report best estimate of your household out-of-pocket spending on lodging in a non-health facility such as a hotel.

If no spending put "0" __ __ __ __ __ __ __ __ __

7 Don't Know

8 Refuse to Answer

9 Not Applicable

Ambulatory care

READ: These next questions are about any ambulatory health care sought or received since [child name] started feeling ill on [Response to Q1] until today. Please, ***include*** care from all types of health facilities such as pharmacy, traditional healer, private clinic, Puskesma primary health center, midwife, hospital outpatient department and/or emergency room, and private lab.

Q26. Did [child name] visit any ambulatory health facility during his/her latest dengue episode? (Show flash card Form 1).

1 Yes

0 No ***Go to Q40***

8 Refuse to Answer ***Go to Q40***

READ: Think about each time [child name] had contacted with one of those health facilities during the dengue episode that started in [date in Q1]. Then specify the type of care [child name] received at each of the health facilities he/she visited (consultations, diagnostic tests and/or treatment) and try to remember the amounts your household, other family members or friends paid out of pocket in total or for each type of service received.

Q27. How many visits did [child name] had to any of the following health facilities during this interview period? ***Show flashcard forms 1 (calendar) and 2 (List of healthcare providers)***

Visit	Number of visits
Pharmacy (01)	
Traditional healer (02)	
Private clinic (03)	

Puskesmas Primary health center (04)	
Midwife (05)	
Outpatient department in a hospital (06)	
Emergency room (07)	
Private lab (08)	
Satellite health center (09)	
Other (10)	
Specify others______________	

Q28. Choose from the following the first *(second, third,..)* type of health facility [child name] contracted during this interview period. Can you give the date of each visit? (*Show flashcard form 2 and form 1 (the calendar*) (Choose one for each visit)

Visit	First	Second	Third	Fourth	Fifth	Sixth
Date						
Type of facility						
Pharmacy (01)						
Traditional healer (02)						
Private clinic (03)						
Puskesmas Primary health center (04)						
Midwife (05)						
Outpatient department in a hospital (06)						
Emergency room (07)						
Private lab (08)						
Satellite health center (09)						
Other (10), Specify_________						
Don’t know (97)						
Refuse to answer (98)						

Read: What is the name of the facility [child name] went to? (Fill in the space next to type of facility the patient chose)
Note for interviewer: A “Public” Facility is a health facility operated by any level of government, and a “social-private” facility is organization run by private non-profit organization, Private is an individual doctor or group of private doctors.

Q29. What type of facility did [child name] visit, during his/her X visit? Was it public or non-public facility? (Choose one per visit)

Visit	Public (1)	Social-private (2)	Private (3)	Don’t know (7)	Refuse to answer (8)
First					
Second					
Third					
Fourth					
Fifth					
Sixth					

Q30. What kind of service did [child name] receive in this facility during his/her X visit? (Check all that apply)

Visit	Consultations	Diagnostic test such as x-ray, blood test	Treatment such as medications, intravenous fluid	Referral	Don’t know (7)	Refuse to answer (8)
First						

Second						
Third						
Fourth						
Fifth						
Sixth						

READ: Now I would like to ask you about the out-of-pocket payments your household, other family members or friend made in this facility.
Note for interviewer: *Whenever possible, cross check the amount with bills available from the health facility.*

Q31. Please report your best estimate of the total out-of-pocket payment for services received in this facility? Please include both payments already made and those that are expected to be made for the services received.
If there were no payments, please enter "0.00".

Visit	Amount, IDR	***If zero skip to instruction before Q36a***	Don't know (7)	***Skip to instruction before Q36a***	Refuse of answer (8)	***Skip to instruction before Q36a***
First						
Second						
Third						
Fourth						
Fifth						
Sixth						

Q32. Can you break down the total payments by type of service received?

Visit	Yes (1)	No (0)	***Skip to instruction before Q36a***	Refuse to answer (8)	***Skip to instruction before Q36a***	Not applicable (9)	***Skip to instruction before Q36a***
First							
Second							
Third							
Fourth							
Fifth							
Sixth							

Q33. Out-of-pocket payment on: Consultation. (*If no payment record "0.00")*

Visit	Amount, IDR	Don't know (7)	Refuse to answer (8)	Not applicable (9)
First				
Second				
Third				
Fourth				
Fifth				
Sixth				

Q34. Out-of-pocket payment on: diagnostic tests or blood bank such as blood test, dengue test, x-rays, sonogram...
If no payment record "0.00".

Visit	Amount, IDR	Don't know (7)	Refuse to answer (8)	Not applicable (9)
First				
Second				
Third				
Fourth				
Fifth				

Sixth				

Q35. Out-of-pocket payment on: treatment such as medications, medical equipment (intravenous sets).
If no payment record "0.00".

Visit	Amount, IDR	Don't know (7)	Refuse to answer (8)	Not applicable (9)
First				
Second				
Third				
Fourth				
Fifth				
Sixth				

READ: Now I would like to ask you questions about other spending your household, other family members or friend, made specific for this facility during this illness episode such as spending on transportation, meals and lodging. Include also any expenses by any other household member who accompanied you to this first (*second, third, ...*) facility for care.

Q36a. Can you please tell me the type of transportation [child's name] used to go to the [TYPE of health facility]?
Note to interviewer: if transportation used was a car, ask about the distance from the patient's house to the [health facility].

Visit	Transportation type	Distance, KM
First		
Second		
Third		
Fourth		
Fifth		
Sixth		

Q36b. Please report the best estimate of out-of-pocket spending on transportation to the [Type of health facility]

If family or owned vehicle, estimate mileage traveled (two ways travel) and use IDR 16,000 per Km as a proxy for the cost of driving. If family or patient owned Auto-rickshaw/own scooter use IDR 14,000 per Km. If no spending record "0.00".

Visit	Amount, IDR	Don't know (7)	Refuse to answer (8)	Not applicable (9)
First				
Second				
Third				
Fourth				
Fifth				
Sixth				

Q36c. Can you please tell me the type of transportation [child's name] used to get back home from the [TYPE of health facility]?

Note to interviewer: if transportation used was a car, ask about the distance from [health facility] to patient's house.

Visit	Transportation type	Distance, KM
First		
Second		
Third		
Fourth		
Fifth		

Sixth		

Q36d. Please report the best estimate of out-of-pocket spending on transportation from the [Type health facility].

If family or owned vehicle, estimate mileage traveled (two ways travel) and use IDR 16,000 per Km as a proxy for the cost of driving. If family or patient owned Auto-rickshaw/own scooter use IDR 14,000 per Km. If no spending record "0.00".

Visit	Amount, IDR	Don't know (7)	Refuse to answer (8)	Not applicable (9)
First				
Second				
Third				
Fourth				
Fifth				
Sixth				

Q37. Please report the best estimate of out-of-pocket spending on food or meals.
If not spending report "0.00"

Visit	Amount, IDR	Don't know (7)	Refuse to answer (8)	Not applicable (9)
First				
Second				
Third				
Fourth				
Fifth				
Sixth				

Q38. Please report the best estimate of out-of-pocket spending on lodging in a non-health facility such as a hotel.
If not spending report "0.00"

Visit	Amount, IDR	Don't know (7)	Refuse to answer (8)	Not applicable (9)
First				
Second				
Third				
Fourth				
Fifth				
Sixth				

Q39. Any other health facilities visited during this illness period from the day you stated feeling ill [Response to Q1] until today?

1 Yes ***Repeat the process starting with Q28 through Q39***
0 No

Illness impact on household members, other family members who reside outside your household or friends during this illness episode

READ: I would like to learn about how [Child's name]'s illness affected you and each of the members of your household**,** other family members who reside outside your household or friends. Household members are people who live with [Child's name]. A household member was affected if he or she spent more time or money than usual caring for the child or lost work, income, or school due to his/her illness.

READ: Let us talk about [Child's name] first.

The Child

Q40. What is the sex of [Child's name]?

1 Male
2 Female

Q41. How old (in years) was your child at his/her last birthday?

_____
999 Don't Know
998 Refuse to answer

Q41a. What is [Child's name] date of birth (day/month and year)?

__/__/____	dd/mm/yyyy
01/01/2097	Don't Know
01/01/2098	Refuse to Answer

Q42. What is the highest level of education you have completed? (Choose one)
Show Flashcard-Form 3

01	Illiterate
02	Just literate but no schooling
03	Attended school for at least one year
04	Pre-school
05	Primary school
06	Middle school
07	High school
08	Diploma
09	Graduate
10	Post graduate (Masters, PhD)
11	Professional degree
97	Don't know
98	Refuse to answer
99	Not applicable

Q43. Is [Child's name] currently studying?

1	Yes	
0	No	***Skip to Q46***
8	Refused to answer	***Skip to Q46***

Q44. Has [Child's name] lost any days of school due to his/her illness?

1	Yes	
0	No	***Skip to Q46***
8	Refused to answer	***Skip to Q46***

Q45. How many days of school did [Child's name] miss due to his/her illness?
Put "0" if none

_____	
97	Don't know
98	Refuse to answer
99	Not applicable

Q46. Is [Child's name] working for pay?

1	Yes	
0	No	***Skip to Q52***
8	Refused to answer	***Skip to Q52***

Q47. Have [Child's name] lost any days of work or income due to his/her illness?
If the patient did not lose any days of work please confirm that he/she did not lose any income.

1 Yes
0 No ***Skip to Q52***
7 Don't know ***Skip to Q52***
8 Refused to answer ***Skip to Q52***

Q48. How many days of work did [Child's name] lose due to his/her illness?
Put "0" if none

_____
97 Don't know
98 Refuse to answer
99 Not applicable

Q49. What was the amount of income lost? (Please give your best estimate)

____
7 Don't know
8 Refused to answer
9 Not applicable

Q50. Did [Child's name] receive any paid sick leave for work days off, in which his/her employer or some agency paid his/her salary while he/she was ill?

1 Yes
0 No ***Skip to Q52***
7 Don't know ***Skip to Q52***
8 Refused to answer ***Skip to Q52***
9 Not applicable ***Skip to Q52***

Q51. How many paid sick days did [Child's name] receive for this illness?
Put "0" if none

_____
97 Don't know
98 Refuse to answer
99 Not applicable

Q52. Did you receive any monetary or non- monetary assistance from family members or the community for this illness episode?

1 Yes
0 No ***Skip to Q55***
8 Refused to answer ***Skip to Q55***

Q53. What is your best estimate of the value of the non- monetary assistance?
True 0 if no non-financial assistance.
Put "0" if none

_____
97 Don't know
98 Refuse to answer
99 Not applicable

Note to interviewer: If don't know, write the type of non-monetary assistance received by patient and patient's family
____
____

Q54. What is the total amount in IDR of the monetary assistance?
Put “0” if none

_____
97 Don’t know
98 Refuse to answer
99 Not applicable

Number of household members

Q55. How many members live in [Child’s name]’s household? (other then you and [Child’s name])?

_____
98 Refuse to answer

Q56. How many of these household members have been affected by [Child’s name]’s illness directly during this illness episode?

READ: A household member is affected if he or she spent more time or money than usual caring for the child or lost work, income, or school due to his/her illness.

_____
00 Zero
98 Refuse to answer

READ: Now I would like to ask you some questions about you and this/these [Response to Q63] member/s of [Child’s name]’s HOUSEHOLD who have been affected by his/her illness.

Care-givers

READ: first I would like to get some information about you.

Q57. What was your age at your last birthday?

____
998 Refuse to answer

Q58. *What is the sex of the proxy?* (*It is not necessary to ask this question aloud)*

0 Male
1 Female

Q59. What is the highest level of education you have completed? (Choose one)
Show Flashcard-Form 3

01 Illiterate
02 Just literate but no schooling
03 Attended school for at least one year
04 Pre-school
05 Primary school
06 Middle school
07 High school
08 Diploma
09 Graduate
10 Post graduate (Masters, PhD)
11 Professional degree
97 Don’t know
98 Refuse to answer
99 Not applicable

Q60. Are you attending school and studying now?

1	Yes	
0	No	***Skip to Q63***
8	Refuse to answer	***Skip to Q63***

Q61. Have you lost any days of school due to [Child's name]'s illness?

1	Yes	
0	No	***Skip to Q63***
8	Refuse to answer	***Skip to Q63***

Q62. How many days of school did you miss due to [Child's name]'s illness?
Put "0" if none

____

97	Don't know
98	Refuse to answer

Q63. Are you working for pay?

1	Yes	
0	No	***Skip to Q69***
8	Refuse to answer	***Skip to Q69***

Q64. Did you lose any days of work due to [Child's name]'s illness?

1	Yes	
0	No	***Skip to Q68a***
8	Refuse to answer	***Skip to Q68a***

Q65. How many days of work did you lose due to [Child's name]'s illness?

____

97	Don't know
98	Refuse to answer
99	Not Applicable

Q66. Did you receive any paid sick leave for work days off, in which your employer or some agency paid your salary while taking care of [Child's name] during his/her illness/?

1	Yes	
0	No	***Skip to Q68a***
7	Don't know	***Skip to Q68a***
8	Refused to answer	***Skip to Q68a***
9	Not applicable	***Skip to Q68a***

Q67. How many paid sick days did you receive for this illness?
Put "0" if none

_____

97	Don't know
98	Refuse to answer
99	Not applicable

Q68a. Did you lose any income due to [Child's name]'s illness?

1	Yes	
0	No	***Skip to Q69***
8	Refuse to answer	***Skip to Q69***

Q68b. What was the amount of income you lost? (Please give your best estimate)

____

7	Don't know
8	Refused to answer
9	Not applicable

Q69. Of the number of sick days during this interview period, what was the total number of days of additional care that you provided to [Child's name]?

____

00	Zero	***Skip to Q71***
97	Don't know	***Skip to Q71***
98	Refuse to answer	***Skip to Q71***
99	Not applicable	***Skip to Q71***

READ: In the questions below, think about the daily number of additional hours of care that [Child's name] have received from you during his/her illness. Additional hours refer to any number of hours above those that you usually spend with [Child's name]. In estimating the time, please include time spent at home, at the health facility and during transportation to and from the health facility

Q70. During these [Response to Q69] days, on average what was the total daily number of additional hours you spent caring for [Child's name]?
If none, enter "0"

____

97	Don't know
98	Refuse to answer
99	Not applicable

Q71. Did you have additional spending because of [Child's name]'s illness such as transportation, meals and lodging other than the expenses previously mentioned for visiting health providers

1	Yes	
0	No	***Skip to instruction before Q73***
7	Don't know	***Skip to instruction before Q73***
8	Refuse to answer	***Skip to instruction before Q73***

Q72. For this question please report the best estimate of the total amount of additional spending over these [Response to Q69] days not already included in one of the previous questions
If no payment record "0.00"

IDR____________________

7	Don't know
8	Refuse to answer

If Q56 is equal to 1 then end interview

Read: Think about the second (third, fourth…) household member who has been affected by [Child's name]'s illness during this interview period

Q73. What is the relationship of this (second, third,..) affected household member to [Child's name]? (Choose one)
Show flashcard form 4

Relationship	Household member						
	Second member	Third member	Fourth member	Fifth member	Sixth member	Seventh member	Eighth member
Mother (01)							
Father (02)							
Husband (03)							

Wife (04)							
Housemate (05)							
Sister (06)							
Brother (07)							
Son (08)							
Daughter (09)							
Grandmother (10)							
Grandfather (11)							
Uncle (12)							
Aunt (13)							
House help (14							
Baby sitter (15)							
Other (16) Specify__________							
Refuse to answer (98)							

Q74. What is [Household member]'s age?

Household member	Age in years	Don't know (997)	Refuse to answer (998)
Second member			
Third member			
Fourth member			
Fifth member			
Sixth member			
Seventh member			
Eighth member			

Q75. What was the highest level of education that [household member] completed? (Choose one)
Show flashcard-form 3

Relationship	Household member					
	Second member	Third member	Fourth member	Fifth member	Seventh member	Eighth member
Illiterate (01)						
Just literal but no schooling (02)						
Attended school for at least one year (03)						
Pre-school (04)						
Primary school (05)						
Middle school (06)						
High school (07)						
Diploma (08)						
Graduate (09)						
Post graduate (Masters, PhD) (10)						
Professional degree (11)						
Don't know (97)						
Refuse to answer (98)						
Not applicable (99)						

Q76. Is [Household member] currently studying?

Household member	Yes (Code:1)	No (Code:2)	***Skip to Q79***	Don't know (Code:7)	***Skip to Q79***	Refuse to answer (Code:8)	***Skip to Q79***
Second member							

Third member							
Fourth member							
Fifth member							
Sixth member							
Seventh member							
Eighth member							

Q77. Has [Household member] lost any days of school due to [Child's name]'s illness?

Household member	Yes (Code:1)	No (Code:2)	***Skip to Q79***	Don't know (Code:7)	***Skip to Q79***	Refuse to answer (Code:8)	***Skip to Q79***
Second member							
Third member							
Fourth member							
Fifth member							
Sixth member							
Seventh member							
Eighth member							

Q78. How many days of school did [household member] missed due to [Child's name]'s illness?
Record "0" if none

Household member	Days of schooling lost	Don't know (97)	Refuse to answer (98)
Second member			
Third member			
Fourth member			
Fifth member			
Sixth member			
Seventh member			
Eighth member			

Q79. Is [Household member] working for pay?

Household member	Yes (Code:1)	No (Code: 0)	***Skip to Q84a***	Don't know (Code:7)	***Skip to Q84a***	Refuse to answer (Code:8)	***Skip to Q84a***
Second member							
Third member							
Fourth member							
Fifth member							
Sixth member							
Seventh member							
Eighth member							

Q80. Has [Household member] lost any days of work due to [Child's name] illness?

Household member	Yes (Code:1)	No (Code: 0)	***Skip to Q84a***	Don't know (Code:7)	***Skip to Q84a***	Refuse to answer (Code:8)	***Skip to Q84a***
Second member							
Third member							
Fourth member							
Fifth member							
Sixth member							
Seventh member							

Eighth member							

Q81. How many days of work did [Household member] lose due to [Child's name] illness?
Record "0" if none

Household member	Number of days	Don't know (Code:97)		Refuse to answer (Code:98)		Not applicable (Code:99)	
Second member							
Third member							
Fourth member							
Fifth member							
Sixth member							
Seventh member							
Eighth member							

Q82. Did [household member] receive any paid sick leave for work days off, in which the employer or some agency paid his/her salary for caring for [Child's name] while you were ill?

Household member	Yes (Code: 1)	***If Zero skip to Q84a***	Don't know (Code:7)	***Skip to Q84a***	Refused to answer (Code:8)	***Skip to Q84a***	Not Applicable (Code:9)	***Skip to Q84a***
Second member								
Third member								
Fourth member								
Fifth member								
Sixth member								
Seventh member								
Eighth member								
Ninth member								

Q83. How many paid sick days did [household member] receive?

Household member	Number of paid sick days	Don't know (97)	Refuse to answer (98)
Second member			
Third member			
Fourth member			
Fifth member			
Sixth member			
Seventh member			
Eighth member			

Q84a. Has [Household member] lost any income due to [Child's name] illness?

Household member	Yes (Code:1)	No (Code: 0)	***Skip to Q85***	Don't know (Code:7)	***Skip to Q85***	Refuse to answer (Code:8)	***Skip to Q85***
Second member							
Third member							
Fourth member							
Fifth member							
Sixth member							
Seventh member							
Eighth member							

Q84b. What was the amount of income [household member] lost? (Please give your best estimate).
If no payment record "0.00".

Household member	Amount, IDR	Don't know (7)	Refuse to answer (8)	Not applicable (9)
Second member				
Third member				
Fourth member				
Fifth member				
Sixth member				
Seventh member				
Eighth member				
Ninth member				

Q85. Of the number of sick days during this illness episode that started on [date of illness till patient date of recovery or date of interview], what was the total number of days of additional care that [household member] provided [Child's name]?

Household member	Number of days	***If Zero skip to Q87***	Don't know (Code:97)	***Skip to Q87***	Refused to answer (Code:98)	***Skip to Q87***	Not Applicable (Code:99)	***Skip to Q87***
Second member								
Third member								
Fourth member								
Fifth member								
Sixth member								
Seventh member								
Eighth member								

READ: In the questions below, think about the daily number of additional hours of care that [Child's name] has received from [household member x] during his/her illness. Additional hours refer to any number of hours above those that [household member x] usually spends with [Child's name}. In estimating the time, please include time spent at home, at the health facility and during transportation to and from the health facility

Q86. During these [Response to Q90] days, on average what was the total daily number of additional hours [household member x] spent caring for [Child's name]?
If none, enter "0"

Household member	Average additional hours of daily care	Don't know (Code: 97)	Refuse to answer (Code: 98)	Not applicable (Code: 99)
Second member				
Third member				
Fourth member				
Fifth member				
Sixth member				
Seventh member				
Eighth member				

Q87. Did [household member x] have additional spending because of [Child's name]'s illness such as transportation, meals and lodging other than the expenses previously mentioned for visiting health providers?

Household member	Yes (Code:1)	No (Code: 0)	***Skip to next household member***	Don't know (Code:7)	***Skip to next household member***	Refused to answer (Code:8)	**Skip to next household member**	Not Applicable (Code:9)	***Skip to next household member***

Second member									
Third member									
Fourth member									
Fifth member									
Sixth member									
Seventh member									
Eighth member									

Q88. For this question please report the best estimate of the total amount of additional spending over these [Response to Q85] days not already included in one of the previous questions
If no payment record "0.00"

Household member	Amount, IDR	Don't know (7)	Refuse to answer (8)	Not applicable (9)
Second member				
Third member				
Fourth member				
Fifth member				
Sixth member				
Seventh member				
Eighth member				

Check 56, if the answer is more than 1 then do repeat Qs 73 through 88 for all the household members listed. If Q56 is equal to 2, then read the sentence below and end interview

READ: Thank you so much for your participation! If you have any questions regarding this interview or the results of this study please contact xxx at [Name of institute] from xx at xxx

Supplementary Information

Cost of dengue illness in Indonesia across hospital, ambulatory and non-medical settings

S4: Questionnaire S4. Pediatric questionnaire (patients under age 18) in Bahasa Indonesia

Kuesioner untuk Pasien Dengue Anak Terkonfirmasi (Usia ≤ 17)

Nama Pasien ______________
Nomor Sampel ______________
Nama orang tua/yang diwawancara ______________
Tanggal Wawancara ______________

Wawancara

Q0. *Nama fasilitas kesehatan tempat pasien dirawat. (Pilih salah satu)*

1 Rumah sakit______________________
2 Puskesmas_________________

Q00. *Yang diwawancara*

1 Pasien
2 Wali pasien

BACAKAN: Sekarang kita akan berbicara mengenai periode penyakit [Nama anak]. Dan saya akan menunjukkan kalender yang akan membantu Anda menjawab pertanyaan berikut.
Tunjukkan kalender (form 1) ke wali dan lanjutkan hinga Q39. Tuliskan "hari ini" pada kotak untuk tanggal hari ini.

Q1. Kapan [Nama anak] mulai sakit? __/__/____ dd/mm/yyyy
Tuliskan "mulai demam" pada tanggal di kalender.
01/01/2097 Tidak tahu
01/01/2098 Menolak menjawab

Q2. Apakah [Nama anak] saat mengalami demam? [Respons terhadap Q1]

1 Ya
0 Tidak ***Lanjut ke Q6***
8 Menolak menjawab ***Lanjut ke Q6***

Q.3 Kapan [nama anak] mulai demam? __/__/____ dd/mm/yyyy
Tuliskan "mulai demam" pada tanggal di kalender
01/01/2097 Tidak tahu
01/01/2098 Menolak menjawab

Q.4 Apakah demam [Nama anak] saat ini telah berakhir?

1 Ya
0 No ***Lanjut ke Q6***
8 Menolak menjawab ***Lanjut ke Q6***

Q.5 Kapan demam [nama anak] berakhir? __/__/____ dd/mm/yyyy
Tuliskan "demam berakhir" pada tanggal di kalender
01/01/2097 Tidak tahu
01/01/2098 Menolak menjawab

Q6. Gejala apa yang [nama anak] rasakan?

1 Nyeri otot
2 Mual
3 Sakit kepala
4 Lainnya

Q6a Gejala lainnya ____
____
____

Q7. Pada keseluruhan periode sakit, kapan [nama anak] merasa itu terburuk?

Tuliskan "hari terburuk" pada tanggal,

__/__/____	dd/mm/yyyy
01/01/2097	Tidak tahu
01/01/2098	Menolak menjawab

Q8. Pada saat interview ini, apakah [nama anak] masih merasa sakit?

1	Ya	***Lanjut ke Q11***
0	Tidak	

Q9. Apakah [nama anak] sudah pulih sepenuhnya dari episode sakit ini?

1	Ya	
0	No	***Lanjut ke Q11***
8	Menolak menjawab	***Lanjut ke Q11***

Q10. Kapan [nama anak] merasa pulih sepenuhnya pada episode sakit ini?

Tuliskan "pulih sepenuhnya" pada tanggal di kalender,

__/__/____	dd/mm/yyyy
01/01/2097	Tidak tahu
01/01/2098	Menolak menjawab

Q11. Berapa lama sejak [nama anak] merasa sakit hingga memutuskan pergi untuk mendapat layanan kesehatan? (Pilih salah satu)

1	Dalam 24 jam
2	Antara 24 and 48 jam
3	Setelah 48 jam
7	Tidak tahu
8	Menolak menjawab

Perawatan yang diterima pasien dari hari merasa sakit hingga saat ini

Jika punya form KDRS, ***Mohon mengisi Q12 dan 13 dari form Rumah Sakit***

Q12. Apakah rumah sakit tempat [nama anak] dirawat adalah milik pemerintah, swasta-sosial atau swasta?

1	Milik pemerintah
2	Swasta - sosial
3	Swasta
7	Tidak tahu
8	Menolak menjawab

Q13. Indikasikan tipe rumah sakit tempat [nama anak] yang paling lama menjalani perawatan rawat inap?

1	Universitas, rumah sakit rujukan atau tersier
2	Rumah sakit lain
7	Tidak tahu
8	Menolak menjawab

Rawat Inap

BACAKAN: Pada pertanyaan selanjutnya saya hendak mencari tahu apakah [nama anak] menghabiskan waktu 1 atau lebih hari di rumah sakit.

Q14. Apakah [nama anak] dirawat inap selama episode sakit tersebut?

1	Ya	
0	Tidak	***Lanjut bacakan instruksi sebelum Q26***
8	Menolak menjawab	***Lanjut bacakan instruksi sebelum Q26***

Q14a. Kapan [nama anak] mulai dirawat rumah sakit?
Tuliskan "masuk" pada tanggal di kalender

___/___/___	dd/mm/yy
01/01/2097	Tidak tahu
01/01/2098	Menolak menjawab

Q14b. Selama masa rawat inap, berapa malam [nama anak] menghabiskan waktu di rumah sakit? __ __ . __ __

98 Menolak menjawab

Q14c. Kapan [nama anak] keluar dari rumah sakit?
Tuliskan "keluar" pada tanggal di kalender

___/___/___	dd/mm/yy
01/01/2097	Tidak tahu
01/01/2098	Menolak menjawab

BACAKAN: Sekarang saya akan menanyakan beberapa pertanyaan terkait durasi raat inap [nama anak], pengeluaran [nama anak] selama perawatan dan pengeluaran lain terkait rawat inap tersebut.

Q15. Dapatkah Anda memberi tahu saya kelas bangsal rawatan Anda selama di rumah sakit?

1. VIP
2. Kelas 1
3. Kelas 2
4. Kelas 3

Q16. Selama masa rawat inap, apakah [nama anak] sempat dirawat di intensive care unit (ICU)?

1 Ya
0 Tidak ***Lanjut bacakan instruksi sebelum Q18***
8 Menolak menjawab ***Lanjut bacakan instruksi sebelum Q18***

Q17. Berapa hari [nama anak] dirawat di ICU? (jika pasien dirawat di ICU selama 1 hari atau kurang, tuliskan 1)

__ __ . __ __
98 Menolak menjawab

BACAKAN: Sekarang saya akan menanyakan mengenai pengeluaran mandiri yang [nama anak] bayar ke fasilitas kesehatan. Pengeluaran mandiri adalah jumlah uang yang [nama anak] atau keluarga [nama anak] bayarkan untuk biaya perawatan [nama anak], baik itu [nama anak] menerima penggantian atau tidak dari jumlah tersebut selama episode sakit [nama anak].

Q18. Berapa estimasi pengeluaran mandiri [nama anak] untuk perawatan yang [nama anak] terima selama rawat inap? (Dapat dibulatkan). Jika tidak ada pengeluaran, masukkan “0”.
Mohon dimasukkan baik pengeluaran yang telah dibayarkan dan pengeluaran yang akan terjadi

__ __ __ __ __ __ __
7 Tidak tahu ***Lanjut bacakan instruksi sebelum Q23***
8 Menolak menjawab ***Lanjut bacakan instruksi sebelum Q23***

Q19. Dapatkah [nama anak] memilah milah pengeluaran [nama anak] berdasar jenis layanan yang [nama anak] dapatkan selama rawat inap?

1 Ya
0 Tidak ***Lanjut bacakan instruksi sebelum Q23***
7 Tidak tahu ***Lanjut bacakan instruksi sebelum Q23***
8 Menolak menjawab ***Lanjut bacakan instruksi sebelum Q23***

Q20. Berapa pengeluaran mandiri rumah tangga [nama anak] yang dibayarkan untuk konsultasi, saat mulai hingga selesai rawat inap?
Jika tidak ada pengeluaran, masukkan "0".

__ __ __ __ __ __ __ __
7 Tidak tahu
8 Menolak menjawab
9 Tidak dapat diaplikasikan

Q21. Berapa pengeluaran mandiri rumah tangga [nama anak] yang dibayarkan untuk uji diagnostik seperti cek darah, tes dengue, rontgent atau sonogram?

Jika tidak ada pengeluaran, masukkan "0". __ __ __ __ __ __ __ __

7 Tidak tahu

8 Menolak menjawab

9 Tidak dapat diaplikasikan

Q22. Berapa pengeluaran mandiri rumah tangga [nama anak] yang dibayarkan untuk pengobatan, cairan infus dan peralatan medis atau bank darah?

Jika tidak ada pengeluaran, masukkan "0". __ __ __ __ __ __ __ __

7 Tidak tahu

8 Menolak menjawab

9 Tidak dapat diaplikasikan

BACAKAN: Sekarang saya akan menanyakan mengenai pengeluaran [nama anak] terkait episode penyakit dari awal hingga selesai merasa sakit [Merespons Q1], seperti untuk transportasi, makan, atau penginapan. Termasuk pengeluaran lain oleh anggota keluarga yang menemani masa rawat inap [nama anak].

Q23a. Apa moda transportasi yang [nama anak] gunakan untuk menuju ke rumah sakit? ________________________

Catatan pewawancara: jika transportasi yang digunakan adalah mobil, tanyakan jarak rumah pasien ke rumah sakit. Jika pasien tidak tahu jawabannya, maka gunakan google map untuk menentukan jaraknya.

Q23b. Berapa estimasi pengeluaran mandiri keluarga Anda untuk transportasi menuju rumah sakit), termasuk ambulans?

Jika tidak ada pengeluaran, masukkan "0".

Jika keluarga memiliki mobil, estimasikan jarak tempuh perjalanan (datang dan pergi) dan gunakan proxy Rp. 16.000 per km. Dan jika pasien menggunakan motor gunakan proxy Rp 14.000 per km. Jika tidak ada pengeluaran, catat "0.00".

__ __ __ __ __ __ __ __ __

7 Tidak tahu

8 Menolak menjawab

9 Tidak dapat diaplikasikan

Q23c. Apa moda transportasi yang [nama anak] gunakan untuk menuju ke rumah sakit? ________________________

Q23d. Berapa estimasi pengeluaran mandiri keluarga Anda untuk transportasi dari rumah sakit), termasuk ambulans?

Jika tidak ada pengeluaran, masukkan "0".

Jika keluarga memiliki mobil, estimasikan jarak tempuh perjalanan (datang dan pergi) dan gunakan proxy Rp. 16.000 per km. Dan jika pasien menggunakan motor gunakan proxy Rp 14.000 per km. Jika tidak ada pengeluaran, catat "0.00".

__ __ __ __ __ __ __ __ __

7 Tidak tahu

8 Menolak menjawab

9 Tidak dapat diaplikasikan

Q24. Berapa estimasi pengeluaran mandiri keluarga [nama anak] untuk makan?
Jika tidak ada pengeluaran, masukkan "0". __ __ __ __ __ __ __ __

7 Tidak tahu
8 Menolak menjawab
9 Tidak dapat diaplikasikan

Q25. Berapa estimasi pengeluaran mandiri keluarga [nama anak] untuk penginapan di fasilitas non-kesehatan seperti hotel?
Jika tidak ada pengeluaran, masukkan "0". __ __ __ __ __ __ __ __

7 Tidak tahu
8 Menolak menjawab
9 Tidak dapat diaplikasikan

Rawat Jalan

BACAKAN: Pertanyaan berikut ini akan terkait layanan rawat jalan sejak [nama anak] mulai merasa hingga berakhirnya sakit [Merespons Q1]. Mohon **termasuk** perawatan dari berbagai jenis fasilitas kesehatan seperti apotek, pengobatan tradisional, klinik swasta, puskesmas, bidan, poli rawat jalan dan/atau unit gawat darurat, dan lab swasta

Q26. Apakah [nama anak] mengunjungi fasilitas rawat jalan pada episode dengue [nama anak] yang terakhir? (Tunjukkan form peraga 1).

1 Ya
0 Tidak ***Lanjutkan ke Q40***
8 Menolak menjawab ***Lanjutkan ke Q40***

BACAKAN: Pikirkan tentang waktu yang [nama anak] habiskan untuk akses fasilitas kesehatan berikut selama episode sakit dimulai **[tanggal pada Q1]**. Kemudian spesifikkan jenis perawatan yang [nama anak] terima pada tiap fasilitas kesehatan yang [nama anak] kunjungi (konsultasi, uji diagnostik, dan/atau pengobatan) dan cobalah mengingat kembali jumlah pengeluaran mandiri keluarga [nama anak] pada tiap jenis layanan tersebut.

Q27. Berapa kali kunjungan pada fasilitas kesehatan berikut ini selama periode sakit [nama anak]?
Tunjukkan form 1 (kalender) dan 2 (daftar fasilitas kesehatan)

Visit	Number of visits
Apotek (01)	
Pengobatan tradisional (02)	
Dokter praktek pribadi (03)	
Puskesmas (04)	
Bidan (05)	
Poli rawat jalan rumah sakit (06)	
Unit Gawat Darurat (UGD) (07)	
Lab swasta (08)	
Puskesmas pembantu (09)	
Lainnya (10)	
Sebutkan lainnya_______________	

Q28. Pilihlah dari berikut ini tipe fasilitas kesehatan yang [nama anak] kunjungi selama periode sakit. Apakah [nama anak] dapat memberi tanggal pada tiap kunjungan? (*Tunjukkan form 2 dan form 1, pilih salah satu pada tiap kunjungan)*

Kunjungan	Pertama	Kedua	Ketiga	Keempat	Kelima	Keenam
Tanggal						
Type of facility						
Apotek (01)						
Pengobatan tradisional (02)						
Dokter praktek pribadi (03)						
Puskesmas (04)						
Bidan (05)						
Poli rawat jalan rumah sakit (06)						
UGD (07)						
Lab swasta (08)						
Puskesmas pembantu (09)						
Lainnya (10), sebutkan__________						
Tidak tahu (97)						
Menolak menjawab (98)						

BACAKAN: Sebutkan nama fasilitas kesehatan manakah yang [nama anak] kunjungi? (Isikan kolom di samping tipe fasilitas kesehatan yang dipilih pasien)
Catatan untuk pewawancara: Fasilitas kesehatan "public" adalah fasilitas kesehatan yang dioperasikan oleh pemerintah dalam berbagai tingkatan, dan "swasta-sosial" adalah fasilitas kesehatan yang dioperasikan oleh organisasi sosial non-profit, "swasta" adalah dokter praktek pribadi atau klinik bersama.

Q29. Apa tipe fasilitas kesehatan yang [nama anak] kunjungi, selama kunjungan kali ke-X [nama anak]? Apakah fasilitas kesehatan public atau non-publik? (Pilih salah satu per kunjungan)

Kunjungan	Publik (1)	Swasta-sosial (2)	Swasta (3)	Tidak tahu (7)	Menolak menjawab (8)
Pertama					
Kedua					
Ketiga					
Keempat					
Kelima					
Keenam					

Q30. Jenis layanan apaa yang [nama anak] terima selama kunjungan kali ke-X [nama anak]? (Cek yang diaplikasikan)

Kunjungan	Konsultasi	Uji diagnostic seperti rontgent, sampel darah	Pengobatan medis, infus	Rujukan	Tidak tahu (7)	Menolak menjawab (8)
Pertama						
Kedua						
Ketiga						
Keempat						
Kelima						
Keenam						

BACAKAN: Sekarang saya akan menanyakan mengenai pengeluaran mandiri keluarga [nama anak] terhadap fasilitas berikut ini.
Catatan pewawancara: *Bila memungkinkan, kroscek dengan jumlah tagihan yang tersedia dari fasilitas kesehatan.*

Q31. Mohon sebutkan perkiraan terbaik [nama anak] dari total pengeluaran mandiri terhadap layanan yang [nama anak] terima dari fasilitas kesehatan? Mohon termasuk pengeluaran yang telah dilakukan dan tagihan yang akan datang. Jika tidak ada pengluaran, mohon masukkan "0,00"

Kunjungan	Jumlah, IDR	***Jika 0 lompat ke instruksi sebelum Q36a***	Tidak tahu (7)	***Lompat ke instruksi sebelum Q36a***	Menolak menjawab (8)	***Lompat ke instruksi sebelum Q36a***
Pertama						
Kedua						
Ketiga						
Keempat						
Kelima						
Keenam						

Q32. Apakah [nama anak] dapat mengurai jumlah pembayaran berdasar tipe layanan yang diterima?

Kunjungan	Ya (1)	Tidak (0)	***Lompat ke instruksi sebelum Q36a***	Menolak menjawab (8)	***Lompat ke instruksi sebelum Q36a***	Tidak dapat diterapkan (9)	***Lompat ke instruksi sebelum Q36a***
Pertama							
Kedua							
Ketiga							
Keempat							
Kelima							
Keenam							

Q33. Pengeluaran mandiri pada: konsultasi. (*Jika tidak ada pembayaran tuliskan "0,00")*

Visit	Jumlah, IDR	Tidak tahu (7)	Menolak menjawab (8)	Tidak dapat diterapkan (9)
Pertama				
Kedua				
Ketiga				
Keempat				
Kelima				
Keenam				

Q34. Pengeluaran mandiri pada: transfusi darah atau uji diagnostic seperti rontgent, sampel darah, sonogram… (*Jika tidak ada pembayaran tuliskan "0,00")*

Kunjungan	Jumlah, IDR	Tidak tahu (7)	Menolak menjawab (8)	Tidak dapat diterapkan (9)
Pertama				
Kedua				
Ketiga				
Keempat				
Kelima				
Keenam				

Q35. Pengeluaran mandiri pada: pengobatan, set peralatan medis.
(*Jika tidak ada pembayaran tuliskan “0,00”)*

Kunjungan	Jumlah, IDR	Tidak tahu (7)	Menolak menjawab (8)	Tidak dapat diterapkan (9)
Pertama				
Kedua				
Ketiga				
Keempat				
Kelima				
Keenam				

BACAKAN: Sekarang saya akan menanyakan terkait pengeluaran keluarga [nama anak] spesifik untuk fasilitas kesehatan selama periode sakit seperti untuk transportasi, makan/minum, dan penginapan. Termasuk pengeluaran anggota keluarga [nama anak] yang menemani selama kunjungan pertama (kedua, ketiga,…).

Q36a. Apakah moda transportasi yang [nama anak] gunakan untuk menuju fasilitas kesehatan?
Catatan pewawancara: jika transportasi yang digunakan adalah mobil, tanyakan jarak dari rumah pasien ke fasilitas kesehatan

Kunjungan	Moda Transportasi	Jarak (km)
Pertama		
Kedua		
Ketiga		
Keempat		
Kelima		
Keenam		

Q36b. Mohon sebutkan perkiraan terbaik [nama anak] terkait pengeluaran mandiri untuk transportasi menuju fasilitas kesehatan)
Jika keluarga memiliki kendaraan, perkirakan jarak tempuh perjalanan (pulang-pergi) dan gunakan IDR 16,000 per Km sebagai acuan dengan mobil dan IDR 14,000 per Km bila dengan motor. Jika tidak ada pengeluaran tuliskan “0,00”.

Kunjungan	Jumlah, IDR	Tidak tahu (7)	Menolak menjawab (8)	Tidak dapat diterapkan (9)
Pertama				
Kedua				
Ketiga				
Keempat				
Kelima				
Keenam				

Q36c. Apakah moda transportasi yang [nama anak] gunakan dari fasilitas kesehatan?
Catatan pewawancara: jika transportasi yang digunakan adalah mobil, tanyakan jarak dari fasilitas kesehatan pasien ke rumah

Kunjungan	Moda Transportasi	Jarak (km)
Pertama		
Kedua		
Ketiga		
Keempat		
Kelima		
Keenam		

Q36b. Mohon sebutkan perkiraan terbaik [nama anak] terkait pengeluaran mandiri untuk transportasi dari fasilitas kesehatan)
Jika keluarga memiliki kendaraan, perkirakan jarak tempuh perjalanan (pulang-pergi) dan gunakan IDR 16,000 per Km sebagai acuan dengan mobil dan IDR 14,000 per Km bila dengan motor. Jika tidak ada pengeluaran tuliskan "0,00".

Kunjungan	Jumlah, IDR	Tidak tahu (7)	Menolak menjawab (8)	Tidak dapat diterapkan (9)
Pertama				
Kedua				
Ketiga				
Keempat				
Kelima				
Keenam				

Q36c. Apakah moda transportasi yang [nama anak] gunakan dari fasilitas kesehatan?
Catatan pewawancara: jika transportasi yang digunakan adalah mobil, tanyakan jarak dari fasilitas kesehatan ke rumah pasien

Kunjungan	Moda Transportasi	Jarak (km)
Pertama		
Kedua		
Ketiga		
Keempat		
Kelima		
Keenam		

Q37. Mohon sebutkan perkiraan terbaik [nama anak] terkait pengeluaran mandiri untuk makan/minum.
Jika tidak ada pengeluaran tuliskan "0,00".

Kunjungan	Jumlah, IDR	Tidak tahu (7)	Menolak menjawab (8)	Tidak dapat diterapkan (9)
Pertama				
Kedua				
Ketiga				
Keempat				
Kelima				
Keenam				

Q38. Mohon sebutkan perkiraan terbaik [nama anak] terkait pengeluaran mandiri untuk penginapan seperti hotel.
Jika tidak ada pengeluaran tuliskan "0,00".

Kunjungan	Jumlah, IDR	Tidak tahu (7)	Menolak menjawab (8)	Tidak dapat diterapkan (9)
Pertama				
Kedua				
Ketiga				
Keempat				
Kelima				
Keenam				

Q39. Apakah ada fasilitas kesehatan lain yang [nama anak] kunjungi selama periode sakit dimulai dari saat [nama anak] mulai merasa sakit? [Respons terhadap Q1]?

1 Ya ***Ulangi proses dari Q28 hingga Q39***
0 Tidak

Dampak penyakit pada anggota keluarga selama periode sakit

BACAKAN: Saya ingin mempelajari bagaimana dampak penyakit [nama anak] mempenaruhi [nama anak] dan tiap angota keluarga. Anggota keluarga terdampak jika dia mengeluarkan waktu dan uang melebihi biasanya untuk merawat anak atau hilangnya waktu kerja, pendapatan, dan waktu sekolah karena sakit tersebut.

BACAKAN: Mari kita bicara tentanng [Nama anak] terlebih dahulu.

Pasien

Q40. *Mohon tuliskan jenis kelamin pasien tanpa menanyakan kepadanya.*

1 Laki-laki
2 Perempuan

Q41. Berapa usia [nama anak] saat ini? _____

999 Tidak tahu
998 Menolak menjawab

Q41a. Kapan tanggal lahir [nama anak] (hari/bulan/tahun)?

__/__/____ dd/mm/yyyy
01/01/2097 Tidak tahu
01/01/2098 Menolak menjawab

Q42. Apa tingkat pendidikan terakhir yang [nama anak] selesaikan? (Pilih salah satu)
Tunjukkan Form 3

01 Tidak dapat baca-tulis
02 Mampu baca-tulis namun tidak sekolah
03 Sekolah tapi hanya satu tahun
04 Taman kanak - kanak
05 Sekolah Dasar
06 Sekolah Menengah Pertama
07 Sekolah Menengah Atas
08 Diploma
09 Universitas, Sarjana
10 Universitas, Pasca Sarjana (Magister, Doktoral)
11 Ijazah profesi
97 Tidak tahu
98 Menolak menjawab
99 Tidak dapat diaplikasikan

Q43. Apakah [nama anak] saat ini sedang menempuh pendidikan?

1 Ya
0 Tidak ***Lanjut ke Q46***
8 Menolak menjawab ***Lanjut ke Q46***

Q44. Apakah [nama anak] kehilangan waktu sekolah [nama anak] selama sakit?

1 Ya
0 Tidak ***Lanjut ke Q46***
8 Menolak menjawab

Q45. Berapa hari sekolah [nama anak] yang hilang karena sakit?
Tuliskan “0” jika tidak ada

_____

97 Tidak tahu
98 Menolak menjawab
99 Tidak dapat diterapkan

Q46. Apakah [nama anak] bekerja dan dibayar?

1 Ya
0 Tidak ***Lanjut ke Q52***
8 Menolak menjawab

Q47. Apakah [nama anak] kehilangan hari kerja dan pendapatan selama sakit?
Jika pasien tidak kehilangan hari kerja, mohon konfirmasi apakah kehilangan pendapatan.

1 Ya
0 Tidak ***Lanjut ke Q52***
7 Tidak tahu ***Lanjut ke Q52***
8 Menolak menjawab ***Lanjut ke Q52***

Q48. Berapa hari kerja [nama anak] yang hilang selama sakit?
Tuliskan “0” jika tidak ada

_____

97 Tidak tahu
98 Menolak menjawab
99 Tidak dapat diterapkan

Q49. Berapa pengeluaran [nama anak] yang hilang? (Berikan perkiraan terbaik [nama anak])

____

7 Tidak tahu
8 Menolak menjawab
9 Tidak dapat diterapkan

Q50. Apakah [nama anak] menerima kompensasi pendapatan selama [nama anak] sakit, apakah itu tempat kerja [nama anak] atau pihak lain yang menanggung selama [nama anak] sakit?

1 Ya
0 Tidak ***Lanjut ke Q52***
7 Tidak tahu ***Lanjut ke Q52***
8 Menolak menjawab ***Lanjut ke Q52***
9 Tidak dapat diterapkan ***Lanjut ke Q52***

Q51. Berapa hari gaji yang [nama anak] terima selama sakit?
Tuliskan “0” jika tidak ada

_____

97 Tidak tahu
98 Menolak menjawab
99 Tidak dapat diterapkan

Q52. Apakah Anda menerima bantuan keuangan atau non-keuangan dari keluarga atau komunitas selama [nama anak] sakit?

1 Ya
0 Tidak ***Lanjut ke Q55***
8 Menolak menjawab ***Lanjut ke Q55***

Q53. Berapa perkiraan terbaik Anda nilai bantuan non-keuangan yang didapatkan?
Benar 0 jika tidak ada bantuan non-keuangan.
Tuliskan “0” jika tidak ada

_____

97 Tidak tahu
98 Menolak menjawab
99 Tidak dapat diterapkan

Catatan pewawancara: *Jika tidak tahu, tuliskan tipe bantuan non-keuangan yang diterima pasien dan keluarga pasien*

____

____

Q54. Berapakah total bantuan keuangan yang didapatkan dalam IDR?
Tuliskan “0” jika tidak ada

_____

97 Tidak tahu
98 Menolak menjawab
99 Tidak dapat diterapkan

Jumlah Anggota Keluarga

Q55. Berapa jumlah anggota keluarga yang tinggal bersama? (selain Anda dan [nama anak])

_____

00 Nol
98 Menolak menjawab

.

Q56. Berapa anggota keluarga yang terdampak sakit [nama anak] secara langsung selama periode sakit [nama anak]?
BACAKAN: Anggota keluarga adalah mereka yang tinggal bersama Anda dan berbagi makanan. Anggota keluarga terdampak jika dia menghabiskan uang dan waktu lebih dari jumlah biasanya karena merawat Anda atau kehilangan waktu kerja, pendapatan, sekolah karena sakit [nama anak].

_____

00 Nol
98 Menolak menjawab

BACAKAN: Sekarang saya akan menanyakan Anda mengenai diri Anda dan anggota keluarga yang terdampak dari penyakit {nama anak].

Yang merawat

BACAKAN: Pertama saya akan menanyakan informasi tentang Anda.

Q57. Berapa umur Anda saat ini?

____

998 Menolak menjawab

Q58. Apa jenis kelamin yang diwawancara*?* (*tidak perlu ditanyakan, cukup diisi)*

0 Laki-laki
1 Perempuan

Q59. Apa tingkat pendidikan terakhir yang Anda selesaikan? (Pilih salah satu)
Tunjukkan Form 3

01	Tidak dapat baca-tulis
02	Mampu baca-tulis namun tidak sekolah
03	Sekolah tapi hanya satu tahun
04	Taman kanak - kanak
05	Sekolah Dasar
06	Sekolah Menengah Pertama
07	Sekolah Menengah Atas
08	Diploma
09	Universitas, Sarjana
10	Universitas, Pasca Sarjana (Magister, Doktoral)
11	Ijazah profesi
97	Tidak tahu
98	Menolak menjawab
99	Tidak dapat diterapkan

Q60. Apakah saat ini Anda tengah menempuh pendidikan?

1	Ya
0	Tidak
8	Menolak menjawab

Q61. Apakah Anda kehilangan waktu sekolah Anda selama [nama anak] sakit?

1	Ya	
0	Tidak	***Lanjut ke Q63***
8	Menolak menjawab	***Lanjut ke Q63***

Q62. Berapa hari sekolah Anda yang hilang karena sakit [nama anak]?
Tuliskan "0" jika tidak ada

_____

97	Tidak tahu
98	Menolak menjawab

Q63. Apakah Anda bekerja dan dibayar?

1	Ya	
0	Tidak	***Lanjut ke Q69***
8	Menolak menjawab	***Lanjut ke Q69***

Q64. Apakah Anda kehilangan hari kerja selama [nama anak] sakit?

1	Ya	
0	Tidak	***Lanjut ke Q69***
8	Menolak menjawab	***Lanjut ke Q69***

Q65. Berapa hari kerja Anda yang hilang selama sakitnya [nama anak]?

____

97	Tidak tahu
98	Menolak menjawab
99	Tidak dapat diterapkan

Q66. Apakah Anda menerima kompensasi pendapatan selama Anda sakit, apakah itu tempat kerja Anda atau pihak lain yang menanggung selama [nama anak] sakit?

1	Ya	
0	Tidak	***Lanjut ke Q68a***
7	Tidak tahu	***Lanjut ke Q68a***
8	Menolak menjawab	***Lanjut ke Q68a***
9	Tidak dapat diterapkan	***Lanjut ke Q68a***

Q67. Berapa hari gaji yang Anda terima selama periode sakit [nama anak]?
Tuliskan “0” jika tidak ada

_____

97	Tidak tahu
98	Menolak menjawab
99	Tidak dapat diterapkan

Q68a. Apakah Anda kehilangan pendapatan selama [nama anak] sakit?

1	Ya	
0	Tidak	***Lanjut ke Q69***
8	Menolak menjawab	***Lanjut ke Q69***

Q68b. Berapa pendapatan Anda yang hilang? (Berikan perkiraan terbaik Anda)

____

7	Tidak tahu
8	Menolak menjawab
9	Tidak dapat diterapkan

Q69. Dari jumlah hari sakit selama periode sakit, berapa jumlah tambahan hari yang Anda berikan ke [Nama anak]?

____

00	Nol	***Lanjut ke Q71***
97	Tidak tahu	***Lanjut ke Q71***
98	Menolak menjawab	***Lanjut ke Q71***
99	Tidak dapat diterapkan	***Lanjut ke Q71***

BACAKAN: Dari pertanyaan berikut, pikirkan tentang jumlah tambahan jam per hari yang [nama anak] dapatkan dari anggota keluarga. Tambahan jam merujuk pada jumlah jam diluar biasanya anggota keluarga menghabiskan waktu bersama [nama anak]. Dalam memperkirakan waktu, mohon masukkan waktu di rumah, pada fasilitas kesehatan dan selama perjalanan menuju dan dari fasilitas kesehatan.

Q70. Selama periode ini [Respons terhadap Q69], berapa rata – rata jumlah tambahan jam Anda untuk merawat [nama anak]?
Jika tidak ada, masukkan 0

____

97	Tidak tahu
98	Menolak menjawab
99	Tidak dapat diterapkan

Q71. Apakah ada pengeluaran transportasi, makan/minum dan penginapan tambahan dari yang telah disebutkan sebelumnya untuk mengunjungi fasilitas kesehatan?

1	Ya	
0	No	***Lanjut ke instruksi sebelum Q73***
7	Tidak tahu	***Lanjut ke instruksi sebelum Q73***
8	Menolak menjawab	***Lanjut ke instruksi sebelum Q73***

Q72. Untuk pertanyaan berikut ini, berikan perkiraan terbaik terhadap jumlah total tambahan pengeluaran dari tambahan hari untuk merawat nama anak] yang belum termasuk yang disebutkan pada pertanyaan sebelumnya? *Jika tidak ada pengeluaran, tuliskan "0,00"*

IDR____________________
7 Tidak tahu
8 Menolak menjawab

Jika Q56 sama dengan 1, maka akhiri wawancara

BACAKAN: Pikirkan mengenai anggota keluarga kedua (ketiga, keempat…) yang terdampak penyakit [Nama anak] selama periode sakit

Q73. Apa hubungan anggota keluarga berikut (pertama, kedua,…) dengan [nama anak]? (pilih salah satu)
Tunjukkan form 4

Hubungan	Anggota Keluarga						
	Anggota kedua	Anggota ketiga	Anggota keempat	Anggota kelima	Anggota keenam	Anggota ketujuh	Anggota kedelapan
Ibu (01)							
Ayah (02)							
Suami (03)							
Istri (04)							
Teman serumah (05)							
Saudara perempuan (06)							
Saudara laki-laki (07)							
Anak laki-laki (08)							
Anak perempuan (09)							
Nenek (10)							
Kakek (11)							
Paman (12)							
Bibi (13)							
Pembantu (14)							
Pengasuh (15)							
Lainnya (16) sebutkan_________							
Menolak menjawab (98)							
Tidak dapat diaplikasikan (99)							

Q74. Berapa usia anggota keluarga?

Anggota keluarga	Usia dalam tahun	Tidak tahu (997)	Menolak menjawab (998)
Anggota kedua			
Anggota ketiga			
Anggota keempat			
Anggota kelima			
Anggota keenam			
Anggota ketujuh			
Anggota kedelapan			

Q75. Apa tingkat pendidikan terakhir anggota keluarga? (pilih salah satu)
Tunjukkan form 3

Hubungan	Anggota Keluarga						
	Anggota kedua	Anggota ketiga	Anggota keempat	Anggota kelima	Anggota keenam	Anggota ketujuh	Anggota kedelapan
Tidak dapat baca-tulis (01)							
Mampu baca-tulis namun tidak sekolah (02)							
Sekolah tapi hanya satu tahun (03)							
Taman kanak - kanak (04)							
Sekolah Dasar (05)							
Sekolah Menengah Pertama (06)							
Sekolah Menengah Atas (07)							
Diploma (08)							
Universitas, Sarjana (09)							
Universitas, Pasca Sarjana (Magister, Doktoral) (10)							
Ijazah profesi (11)							
Tidak tahu (97)							
Menolak menjawab (98)							

Q76.Apakah ada anggota keluarga saat ini sedang menempuh pendidikan?

Anggota keluarga	Ya (kode:1)	Tidak (Kode:2)	***Lanjut ke Q79***	Tidak tahu (Kode:7)	***Lanjut ke Q79***	Menolak menjawab (Kode:8)	***Lanjut ke Q79***
Anggota kedua							
Anggota ketiga							
Anggota keempat							
Anggota kelima							
Anggota keenam							
Anggota ketujuh							
Anggota kedelapan							

Q77. Apakah ada anggota keluarga kehilangan hari sekolah karena penyakit [nama anak]?

Anggota keluarga	Ya (kode:1)	Tidak (Kode:2)	***Lanjut ke Q79***	Tidak tahu (Kode:7)	***Lanjut ke Q79***	Menolak menjawab (Kode:8)	***Lanjut ke Q79***
Anggota kedua							
Anggota ketiga							
Anggota keempat							
Anggota kelima							
Anggota keenam							
Anggota ketujuh							
Anggota kedelapan							

Q78. Berapa hari sekolah anggota keluarga yang hilang karena penyakit [nama anak]?
Tuliskan "0" jika tidak ada

Anggota keluarga	Jumlah hari yang hilang	Tidak tahu (97)	Menolak menjawab (98)
Anggota kedua			
Anggota ketiga			
Anggota keempat			
Anggota kelima			
Anggota keenam			
Anggota ketujuh			
Anggota kedelapan			

Q79. Apakah anggota keluarga bekerja dan dibayar?

Anggota keluarga	Ya (kode:1)	Tidak (Kode:2)	***Lanjut ke Q84a***	Tidak tahu (Kode:7)	***Lanjut ke Q84a***	Menolak menjawab (Kode:8)	***Lanjut ke Q84a***
Anggota kedua							
Anggota ketiga							
Anggota keempat							
Anggota kelima							
Anggota keenam							
Anggota ketujuh							
Anggota kedelapan							

Q80. Apakah ada anggota keluarga kehilangan hari kerja karena penyakit [nama anak]?

Anggota keluarga	Ya (kode:1)	Tidak (Kode:2)	***Lanjut ke Q84a***	Tidak tahu (Kode:7)	***Lanjut ke Q84a***	Menolak menjawab (Kode:8)	***Lanjut ke Q84a***
Anggota kedua							
Anggota ketiga							
Anggota keempat							
Anggota kelima							
Anggota keenam							
Anggota ketujuh							
Anggota kedelapan							

Q81. Berapa hari kerja anggota keluarga yang hilang karena penyakit [nama anak]?
Tuliskan "0" jika tidak ada

Anggota keluarga	Jumlah hari	Tidak tahu (Kode:97)	***Lanjut ke Q85***	Menolak menjawab (Kode:98)	***Lanjut ke Q85***	Tidak dapat diterapkan (Kode:99)	***Lanjut ke Q85***
Anggota kedua							
Anggota ketiga							
Anggota keempat							
Anggota kelima							
Anggota keenam							
Anggota ketujuh							
Anggota kedelapan							

Q82. Apakah anggota keluarga menerima kompensasi atas absennya mereka dari pekerjaan ketika merawat [nama anak] sakit? (dari tempat kerja atau pihak lain yang menanggung)

Anggota keluarga	Ya (Kode: 1)	Tidak (Kode: 0)	***Lanjut ke Q84a***	Tidak tahu (Kode:7)	***Lanjut ke Q84a***	Menolak menjawab (Kode:8)	**Lanjut ke Q84a**	Tidak dapat diterapkan (Kode:9)	***Lanjut ke Q84a***
Anggota kedua									
Anggota ketiga									
Anggota keempat									
Anggota kelima									
Anggota keenam									
Anggota ketujuh									
Anggota kedelapan									

Q83. Berapa hari yang dibayarkan ke anggota keluarga?

Anggota keluarga	Jumlah hari yang dibayarkan	Tidak tahu (97)	Menolak menjawab (98)
Anggota kedua			
Anggota ketiga			
Anggota keempat			
Anggota kelima			
Anggota keenam			
Anggota ketujuh			
Anggota kedelapan			

Q84a. Apakah anggota keluarga Anda kehilangan pendapatan karena penyakit [nama anak]?

Anggota keluarga	Ya (kode:1)	Tidak (Kode:2)	***Lanjut ke Q85***	Tidak tahu (Kode:7)	***Lanjut ke Q85***	Menolak menjawab (Kode:8)	***Lanjut ke Q85***
Anggota kedua							
Anggota ketiga							
Anggota keempat							
Anggota kelima							
Anggota keenam							
Anggota ketujuh							
Anggota kedelapan							

Q84b. Berapa pendapatan anggota keluarga yang hilang? (Mohon berikan perkiraan terbaik).
Jika tidak ada tuliskan "0,00".

Anggota keluarga	Jumlah, IDR	Tidak tahu (7)	Menolak menjawab (8)	Tidak dapat diterapkan (9)
Anggota kedua				
Anggota ketiga				
Anggota keempat				
Anggota kelima				
Anggota keenam				
Anggota ketujuh				
Anggota kedelapan				

Q85. Dari jumlah hari sakit selama episode sakit [nama anak] yang dimulai sejak sakit hingga saat pemulihan (tanggal wawancara), berapa jumlah hari tambahan yang dihabiskan anggota keluarga untuk perawatan lanjutan [nama anak]?
Jika tidak ada, masukkan 0

Anggota keluarga	Jumlah hari	***Jika "Nol" Lanjut ke Q87***	Tidak tahu (Kode:97)	***Lanjut ke Q97***	Menolak menjawab (Kode:98)	**Lanjut ke Q87**	Tidak dapat diterapkan (Kode:99)	***Lanjut ke Q87***
Anggota kedua								
Anggota ketiga								
Anggota keempat								
Anggota kelima								
Anggota keenam								
Anggota ketujuh								
Anggota kedelapan								

BACAKAN: Dari pertanyaan berikut, pikirkan tentang jumlah tambahan jam per hari yang Anda dapatkan dari anggota keluarga. Tambahan jam merujuk pada jumlah jam diluar biasanya anggota keluarga menghabiskan waktu bersama Anda. Dalam memperkirakan waktu, mohon masukkan waktu di rumah, pada fasilitas kesehatan dan selama perjalanan menuju dan dari fasilitas kesehatan.

Q86. Selama periode ini, berapa rata – rata jumlah tambahan jam anggota keluarga untuk merawat [nama anak]? [Respons terhadap Q68]
Jika tidak ada, masukkan 0

Anggota keluarga	Rata-rata tambahan jam untuk perawatan harian	Tidak tahu (Kode:97)	Menolak menjawab (Kode:98)	Tidak dapat diterapkan (Kode:99)
Anggota kedua				
Anggota ketiga				
Anggota keempat				
Anggota kelima				
Anggota keenam				
Anggota ketujuh				
Anggota kedelapan				

Q87. Apakah ada pengeluaran transportasi, makan/minum dan penginapan tambahan dari yang telah disebutkan sebelumnya untuk mengunjungi fasilitas kesehatan?

Anggota keluarga	Ya (Kode:1)	No (Kode: 0)	***Lanjut ke anggota keluarga lainnya***	Tidak tahu (Kode:7)	***Lanjut ke anggota keluarga lainnya***	Menolak menjawab (Kode:8)	***Lanjut ke anggota keluarga lainnya***	Tidak dapat diterapkan (Kode:9)	***Lanjut ke anggota keluarga lainnya***
Anggota kedua									
Anggota ketiga									
Anggota keempat									
Anggota kelima									
Anggota keenam									
Anggota ketujuh									
Anggota kedelapan									

Q88. Untuk pertanyaan berikut ini, berikan perkiraan terbaik terhadap jumlah total tambahan pengeluaran dari tambahan hari untuk merawat Anda yang belum termasuk yang disebutkan pada pertanyaan sebelumnya? [Jumlah hari yang dilaporkan pada Q68]

Jika tidak ada pengeluaran, tuliskan "0,00".

Anggota keluarga	Amount, IDR	Tidak tahu (7)	Menolak menjawab (8)	Tidak dapat diterapkan (9)
Anggota kedua				
Anggota ketiga				
Anggota keempat				
Anggota kelima				
Anggota keenam				
Anggota ketujuh				
Anggota kedelapan				

Cek Q56, jika jawabannya lebih dari 1, maka ulangi Q57-71 untuk semua anggota keluarga terdaftar. Jika Q56 sama dengan 1, maka bacakan kalimat berikut untuk mengakhiri wawancara.

BACAKAN: Terima kasih atas partisipasi Anda! Jika ada pertanyaan lebih lanjut terkait wawancara atau hasil penelitian ini, Anda dapat menghubungi saya, dr. Nandyan N. Wilastonegoro, MScIH di nomor 0813-2884-3888 atau 0274-5306089 di Fakultas Kedokteran, Kesehatan Masyarakat dan Keperawatan, Universitas Gadjah Mada (FKKMK UGM).

Supplementary Information

Cost of dengue illness in Indonesia across hospital, ambulatory and non-medical settings

Table Contents

S5: Table S5. Studies on cost of a dengue episode in the literature by country

S6: Table S6. Studies on cost of a dengue episode in the literature by region

S7: Table S7. Dengue economic burden by type of case and source of financing (in millions of 2017 US$)

Table S5. Studies on cost of a dengue episode in the literature by country

Study and publication year	Location and data year	Hospital cases		Ambulatory cases	
		Original	Adjusted	Original	Adjusted
		Asian studies			
Vo et al.[1]	Vietnam, 2012	48.10	111.29	NA	NA
Edillo et al.[2]	Philippines, 2012	1,048.73	1,245.81	259.18	307.88
Shepard et al.[3]	Malaysia, 2010	433.23	151.10	210.14	73.29
Tran et al. [4]	Vietnam, 2017	110.10	254.74	36.10	83.53
Nadjib et al., 2019 [5]	Indonesia, 2015	458.14	472.83	59.29	61.19
Hariharan et al. [6]	India, 2013	276.10	643.02	14.25	33.19
Harving & Rönsholt [7]	Vietnam, 2005	61.36	344.78	NA	NA
Tozan et al.[8]	Thailand, 2015	157.68	97.76	NA	NA
Average		NA		NA	
		Latin American studies			
Armien et al. [9]	Panama, 2005	1,065	778.95	332	242.83
Undurraga et al., 2015[10]	Mexico, 2012	240.04	87.15	65.53	23.79
Halasa et al., 2012[11]	Puerto Rico, 2010	5,497.00	1,150.08	1,236	258.60
Martelli et al., 2015[12]	Brazil, 2013	618.33	171.95	181.83	50.56
		Other regions			
Suaya et al., 2009[13]	Brazil, 2005	676.00	601.27	291.00	258.83
	El Salvador, 2005	457.00	668.50	88.00	128.73
	Guatemala, 2005	418.00	736.18	88.00	154.99

Study and publication year	Location and data year	Hospital cases		Ambulatory cases	
		Original	Adjusted	Original	Adjusted
	Panama, 2005	1,065.00	778.95	332.00	242.83
	Venezuela, 2005	672.00	452.05	168.00	121.12
	Cambodia, 2005	115.00	885.00	NA	NA
	Malaysia, 2005	947.00	634.92	317.00	212.53
	Thailand, 2005	573.00	727.03	NA	NA
Shepard et al., 2013[3]	Bhutan, 2010	207.30	372.51	62.30	111.95
	Brunei, 2010	2,481.00	263.75	809.70	86.08
	Cambodia, 2010	116.08	724.99	23.46	110.73
	Timor Leste, 2010	66.00	83.15	19.20	24.19
	Indonesia, 2010	289.10	406.12	87.60	123.06
	Laos, 2010	107.20	1,023.41	31.60	310.10
	Malaysia, 2010	863.21	368.61	422.20	180.29
	Myanmar, 2010	81.50	335.48	23.90	98.38
	Philippines, 2010	212.20	304.12	63.70	91.29
	Singapore, 2010	3008.47	1,268.28	1268.28	237.78
	Thailand, 2010	634.87	490.71	158.72	122.68
	Viet Nam, 2010	76.41	216.39	31.46	89.09

Notes: Nadjib et al. [5] examined cost across three sites from Indonesia: Yogyakarta, Bali and Jakarta. We extracted the costs by site and by setting from their Table 2 to calculate the arithmetic averages of for both hospital and ambulatory services. Tozen et al. [8] examined cost based on diagnosis of DF and DHF per adult and pediatric case. Similarly, we used data from their Table 5 to calculate the arithmetic average cost of the diagnosis and age group. Martelli et al. [12] examined cost across six sites from Brazil both public and private sectors: Goiania, Belo Horizonte, Rio de Janeiro, Teresina, Recife, and Belem. We used data in their Table 3 to calculate the arithmetic average of the three sites for both hospital and ambulatory services. NA denotes not applicable.

Table S6. Studies on cost of a dengue episode in the literature by region

Study and publication year	Location and data year	Hospital cases		Ambulatory cases	
		Original	Adjusted*	Original	Adjusted*
Shepard et al., 2016[14]	Global average, 2013	389.00	126.92	106	14.36
	Central Europe, eastern Europe, Central Asia, 2013	287.00	99.87	64	22.27
	High-income, 2013	3809.00	320.07	678	56.97
	Latin America and the Caribbean, 2013	1360.00	477.97	160	56.23
	North Africa and the Middle East, 2013	333.00	135.54	75	30.54
	South Asia, 2013	260.00	634.62	35	85.43
	Southeast Asia, east Asia, and Oceania, 2013	447	NA	117	NA
	Sub-Saharan Africa, 2013	206	425.14	44	90.81

Shepard et al., 2011[15]	North America, 2010	11,340.50	827.25	1,477.50	107.78
	Latin America and The Caribbean, 2010	2,126.33	946.04	590.43	262.69

* Adjusted numbers are scaled to the ratio of the per capita GNI of the region or country compared to that in Indonesia.

Table S7. Dengue economic burden by type of case and source of financing (in millions of 2017 US$)

**Location**	**Household resources**	**Family/friend contribution**	**JKN**	**Other**	**Total**
			Non-fatal cases only		
	140.07	75.45	95.03	69.13	379.69
National	(33.87-740.36)	(21.57-274.91)	(26.84-365.98)	(18.81-297.07)	(101.09-1,678.32)
Percentage	37%	20%	25%	18%	100%
Yogyakarta	0.51	0.30	0.39	0.28	1.47
City	(0.09-20.06)	(0.07--2.42)	(0.08-4.74)	(0.06-5.73)	(0.30-32.95)
	34%	20%	26%	19%	100%
			All cases (including fatal)		
	441.65	75.45	95.03	69.13	681.26
	(165.06-	(21.57-274.91	(26.84-365.98)	(18.81-297.07)	(232.28-2,371.56)
National	1433.60)				
Percentage	65%	11%	14%	10%	100%

Supplementary Information

Cost of dengue illness in Indonesia across hospital, ambulatory and non-medical settings

Figure Contents

S8: Figure S8. Histogram of direct medical cost per hospitalized case in Yogyakarta, Indonesia (n=20)

S9: Figure S9. Histogram of non-medical cost per hospitalized case in Yogyakarta, Indonesia (n=20)

S10: Figure S10. Histogram of indirect cost per hospitalized case in Yogyakarta, Indonesia (n=20)

S11: Figure S11. Histogram of total cost per hospitalized case in Yogyakarta, Indonesia (n=20)

Figure S8. Histogram of direct medical cost per hospitalized case in Yogyakarta, Indonesia (n=20)

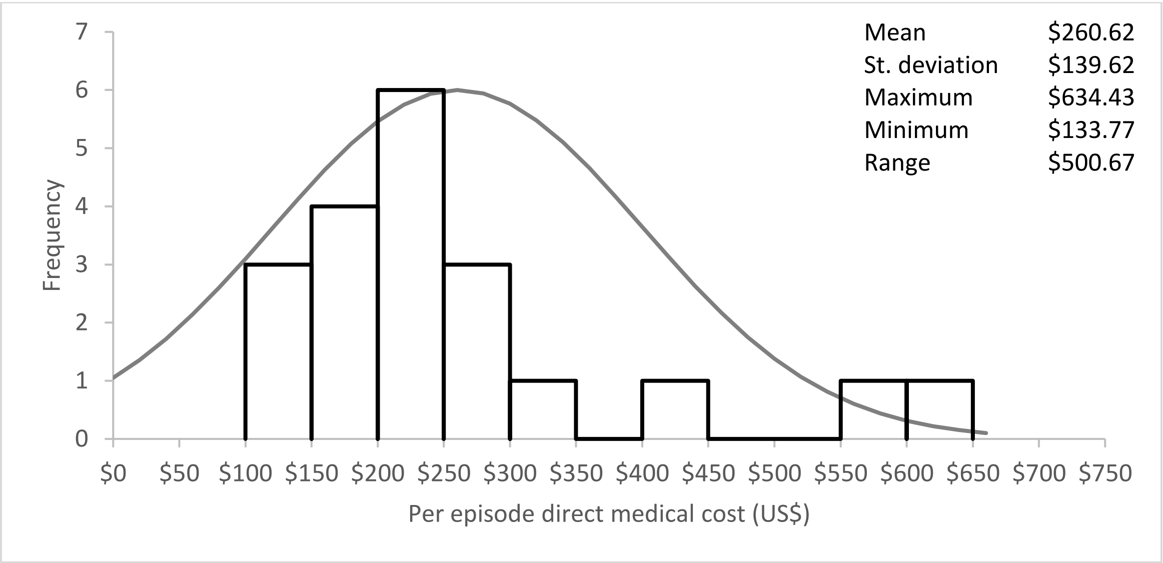

Notes: St. deviation denotes standard deviation; US$ denotes United States dollars

Figure S9. Histogram of non-medical cost per hospitalized case in Yogyakarta, Indonesia (n=20)

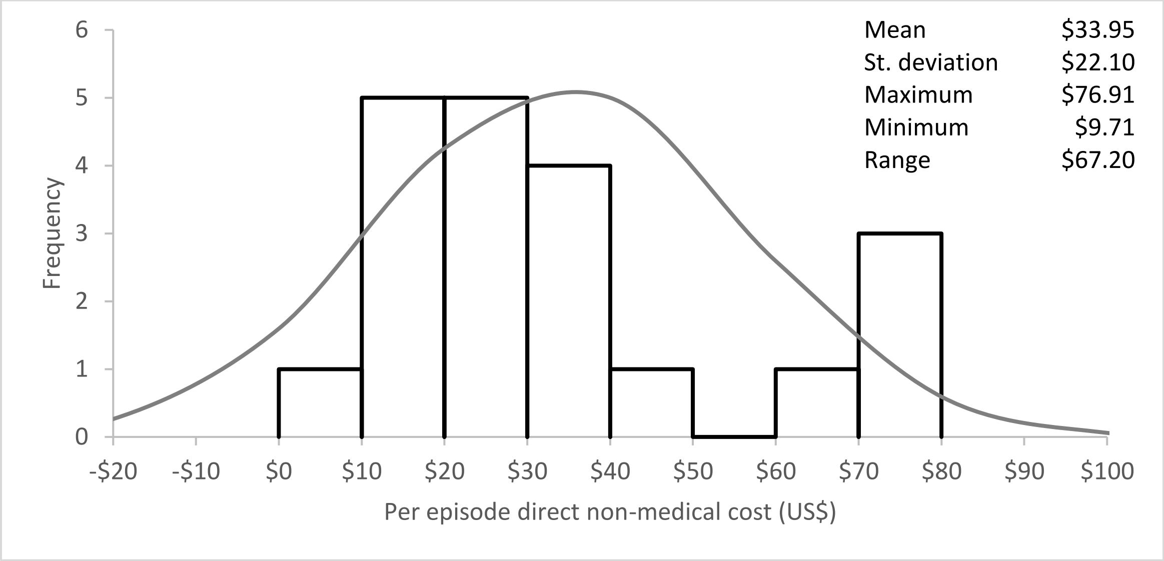

Notes: St. deviation denotes standard deviation; US$ denotes United States dollars

Figure S10. Histogram of indirect cost per hospitalized case in Yogyakarta, Indonesia (n=20)

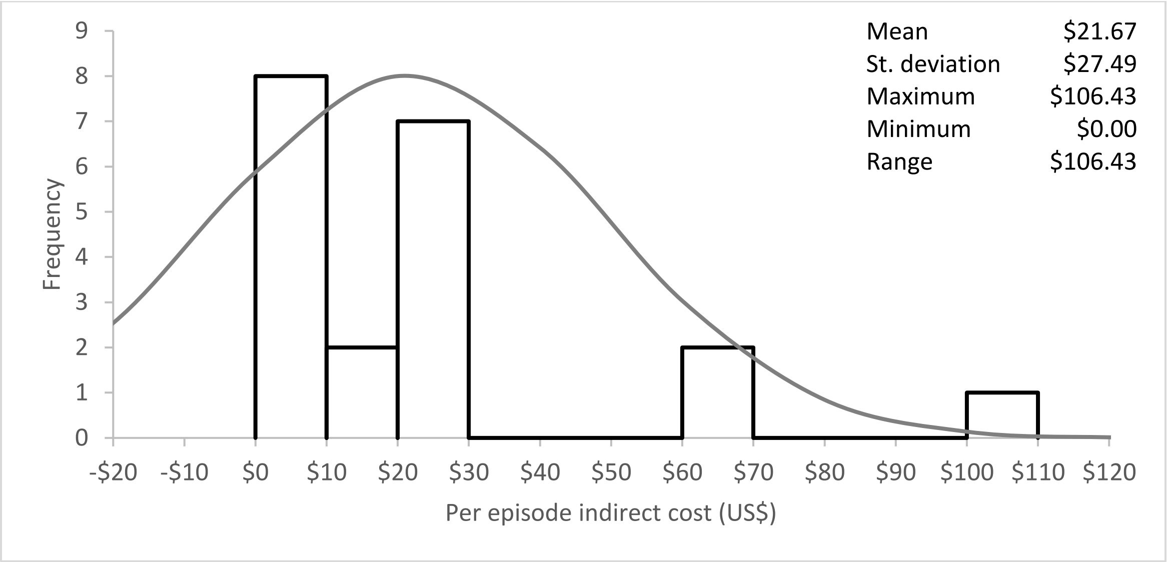

Notes: St. deviation denotes standard deviation; US$ denotes United States dollars

10 Figure S11. Histogram of total cost per hospitalized case in Yogyakarta, Indonesia (n=20)

11 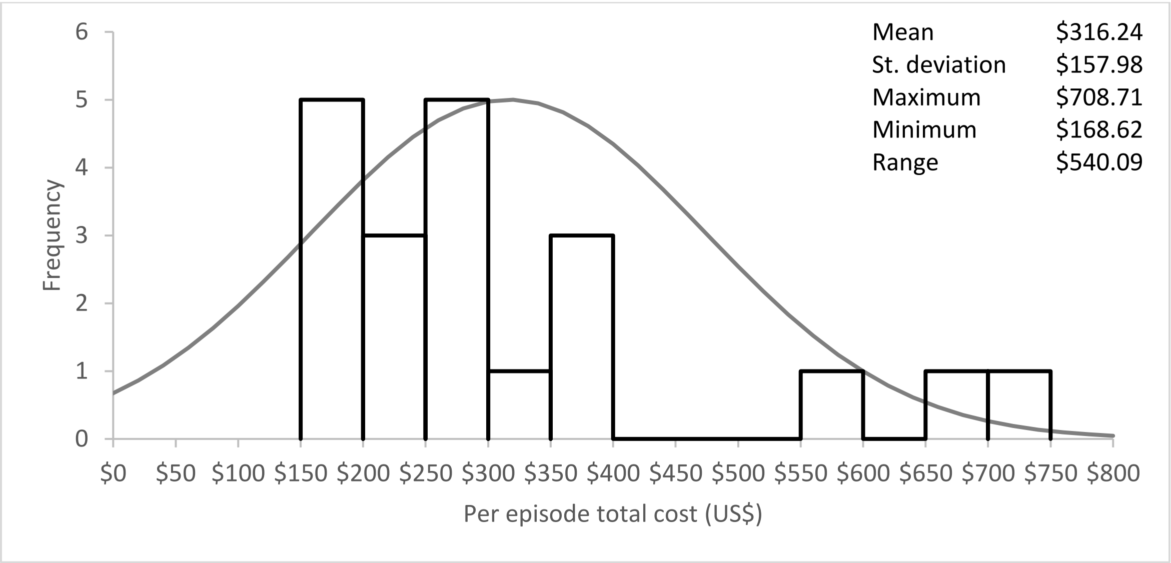

12 Notes: St. deviation denotes standard deviation; US$ denotes United States dollars
